# Blebbistatin Effects Expose Hidden Secrets in the Force-Generating Cycle of Actin and Myosin

**DOI:** 10.1016/j.bpj.2018.05.037

**Published:** 2018-07-17

**Authors:** Mohammad A. Rahman, Marko Ušaj, Dilson E. Rassier, Alf Månsson

**Affiliations:** 1 Department of Chemistry and Biomedical Sciences, Linnaeus University, Kalmar, Sweden; 2 Department of Kinesiology and Physical Education, McGill University, Montreal, Canada

## Abstract

Cyclic interactions between myosin II motors and actin filaments driven by ATP turnover underlie muscle contraction and have key roles in the motility of nonmuscle cells. A remaining enigma in the understanding of this interaction is the relationship between the force-generating structural change and the release of the ATP-hydrolysis product, inorganic phosphate (Pi), from the active site of myosin. Here, we use the small molecular compound blebbistatin to probe otherwise hidden states and transitions in this process. Different hypotheses for the Pi release mechanism are tested by interpreting experimental results from in vitro motility assays and isolated muscle fibers in terms of mechanokinetic actomyosin models. The data fit with ideas that actomyosin force generation is preceded by Pi release, which in turn is preceded by two serial transitions after/coincident with cross-bridge attachment. Blebbistatin changes the rate limitation of the cycle from the first to the second of these transitions, uncovering functional roles of an otherwise short-lived pre-power stroke state that has been implicated by structural data.

## Introduction

Muscle contraction results from cyclic interactions of myosin II motor domains with actin filaments driven by ATP turnover. This process underlies bodily movements powered by skeletal muscle, pumping of blood by the heart, and a range of homeostatic mechanisms governed by smooth muscle. Additionally, nonmuscle myosin II and actin have pivotal roles in cell motility and thereby in functions associated with the immune system, synaptic plasticity, and cell division (1). Therefore, disturbed myosin II function is central to a range of diseases (2, 3, 4) as well as to functional decline during aging (5). Such characteristics have motivated the development of myosin-active small molecular compounds for therapeutic use (2, 3, 6, 7, 8). However, the insights into diseases as well as the associated drug discovery processes are hampered by remaining enigmas in the fundamental understanding of the force-generating cross-bridge cycle between myosin II and actin. Particularly, the relationship between actomyosin cross-bridge formation, phosphate (Pi) release from the myosin active site, and the force-generating structural change is poorly understood with a range of conflicting views (9, 10, 11, 12, 13, 14, 15, 16, 17, 18, 19, 20, 21) (reviewed in (22, 23, 24)). Outstanding questions include but are not limited to the following. 1) Does Pi release occur before (11, 19, 20, 25) or after (12, 15, 17, 26) force generation? Are Pi release and force generation loosely coupled (14)? Are branched kinetic paths necessary to account for Pi effects (27)? 2) Which transition(s) is/are rate limiting for Pi release, force generation, and actomyosin ATPase (9, 13, 23, 28, 29)? 3) How does the fast force generation in response to rapid length changes (30) fit into the picture (11, 23, 31)? Naturally, these questions are of highest fundamental importance because the force-generating process and its relationship to rate-limiting transitions as well as to the kinetic step with the largest drop in free energy (Pi release) is at the core of the energy transduction process (19, 23, 32). Accordingly, several drugs and toxins (33, 34, 35, 36, 37, 38, 39, 40, 41, 42, 43, 44, 45) affect transitions and states in this phase of the cycle with substantial effects both on the actomyosin ATPase in solution, force generation, and shortening velocity. However, consistent with the limited insight into the process, it has been difficult to arrive at a full understanding of the mechanism of action of a range of myosin active substances (35, 38, 39, 46, 47).

**Figure 1:**
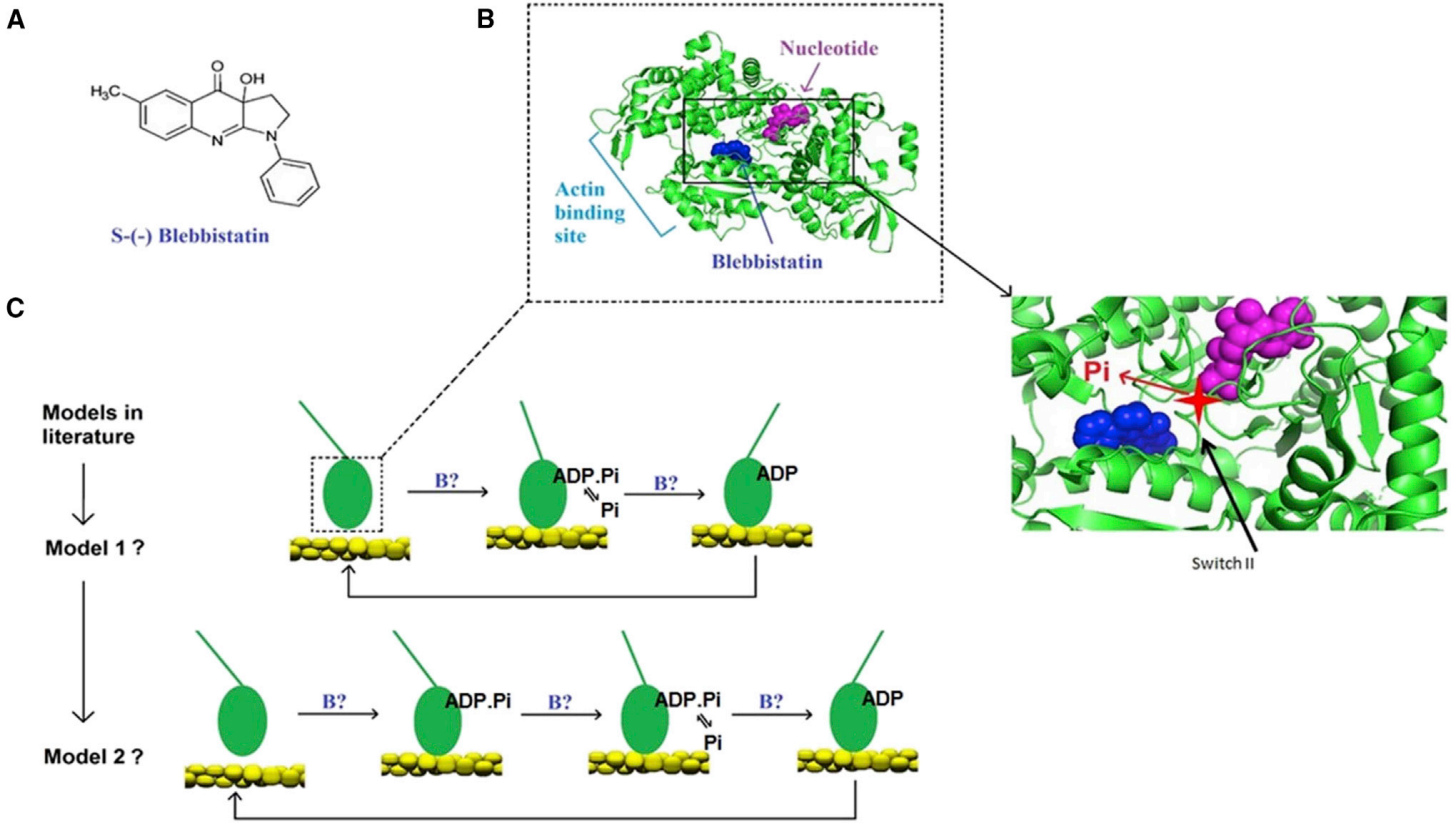
Blebbistatin probing the mechanism for phosphate release and force generation. (*A*) The molecular structure of S-(−) blebbistatin is shown. (*B*) The motor domain of dictyostelium myosin II (48) shows blebbistatin at its binding site and ADP at the active site. The further enlarged image to the right illustrates the proposed molecular model for phosphate release (inorganic phosphate molecule indicated by *star*). The figure was produced by PyMOL 2.0 using the deposited structure with Protein Data Bank: 1YV3 showing the myosin II motor domain with blebbistatin and ADP. Vanadate, magnesium and 1,2-ethanediol present in the deposited structure are excluded. (*C*) Two models (model 1 (29) and model 2 (19, 24)) were selected for final testing based on well-founded arguments from previous experimental and theoretical studies, as considered in detail in the Discussion. The schematic drawing illustrates the myosin motor domain either bound or not bound to actin (*double helical structure*) and with different lever arm (*straight lines*) positions. Hypothetical blebbistatin-affected transitions tested below are indicated by “B?.” The dashed rectangle encloses the myosin motor domain illustrated in molecular detail in (*B*). To see this figure in color, go online.

One small molecular compound that has been extensively studied is the myosin II selective inhibitor blebbistatin, a 1-phenyl-2-pyrrolidinone derivative (40, 41, 42, 43, 48) (Fig. 1 *A*). Because of its incompletely understood effects on Pi release, more detailed studies have the potential to expose hidden states and transitions that cannot be readily probed otherwise (cf. (33)). One advantage of using chemical substances instead of specific mutations for functional studies of this type is that it facilitates the use of a range of experimental preparations, from isolated single molecules to contractile cells investigated in situ. Substances of particular value in this context are those, such as blebbistatin, whose binding site on myosin is well characterized (Fig. 1 *B*). This compound and its derivatives (e.g., (49, 50)) are known to selectively inhibit the actin-activated ATPase of myosin II (40, 41, 42, 43), causing accumulation of cross-bridges in a pre-power-stroke state with ADP and inorganic phosphate (Pi) at the active site (47, 51). Whereas this mechanism of action appears deceivingly simple, the full picture is complex. Particularly, as outlined above, the affected part of the actomyosin cross-bridge cycle remains poorly understood despite intense investigations (9, 10, 11, 12, 13, 14, 15, 16, 17, 18, 19, 21). Second, a blebbistatin-induced decrease in the unloaded shortening velocity in muscle cells only occurs when the myosin regulatory light chains (RLCs) are phosphorylated, whereas the effect on isometric force is independent of phosphorylation (52). Finally, blebbistatin has also been found (44) to stabilize the start of the power-stroke actomyosin state without Pi at the active site.

Here, we performed in vitro motility assay experiments and mechanical studies of muscle fibers in the presence and absence of blebbistatin to allow testing of different mechanokinetic models for the actomyosin cross-bridge cycle (Fig. 1 *C*). These models connect the states and transitions found in biochemical and structural studies to contractile function. The simplest model tested is identical to that in (29). Whereas this model (model 1 in Fig. 1 *C*) accounts for a wide range of experimental data in the absence of blebbistatin, it failed to fully account for the blebbistatin effects. We therefore extended model 1 by introducing states and transitions similar to those proposed by Llinas et al. (19) based on structural and reverse genetics studies of several myosin classes. The expanded model (model 2 in Fig. 1 *C*) accounts for all our new experimental results and, to the best of our knowledge, also for experimental blebbistatin data reported elsewhere (see Discussion). Importantly, therefore, our results corroborate the functional significance of a sequence of events from cross-bridge attachment to phosphate release and force generation proposed recently (18, 19, 25). Furthermore, the results give a solid platform for addressing all the outstanding questions mentioned above as well as for analyzing the effects of other relevant drugs, toxins, and mutations.

## Materials and Methods

### Ethical statement

Rabbits were kept and sacrificed using procedures approved by the Regional Ethical Committee for Animal Experiments in Linköping, Sweden (reference number 73-14).

### In vitro motility assays

In vitro motility assays were performed as described previously using actin (53) and heavy meromyosin (HMM) (54) from fast rabbit muscle. Also, flow cells with silanized surfaces, incubation conditions, assay solutions, recording of motility assay data, and subsequent analysis were described earlier (55). The flow cells were incubated with HMM at 120 or 30 *μ*g/mL (Fig. S6) for 2–5 min. The assay solution had [MgATP] = 1 mM, an ionic strength of 130 mM, and contained 0.64% methylcellulose unless stated otherwise. Temperature was 27.6–30.5°C, and pH = 7.4. HMM with and without phosphorylated RLCs were prepared essentially as described in (56) and characterized using urea gels (57). Blebbistatin was either (±)-blebbistatin or S-(−) blebbistatin.

### Muscle fiber experiments

Experiments using skinned skeletal muscle fibers from fast rabbit muscle were performed as described previously (51). Fibers were activated in the presence or absence of S-(−) blebbistatin (*n* = 16). All experiments were performed at 5°C, and the initial sarcomere length was adjusted to ∼2.5 *μ*m (optimal length, *L*o) before fiber activation.

### Modeling

The mechanokinetic model was developed from that in (29) with the addition of all blebbistatin-bound states and two blebbistatin-free states (AM^∗^’DP and AM^∗^DP). The parameters that define the model for temperatures in the range of 25–30°C are given in Tables S1 and S2. These parameter values are essentially the same as those used previously (29) except for those related to the AM^∗^'DP and the AM^∗^DP states. Unless otherwise stated, the model predictions were derived by Monte Carlo simulations using the Gillespie algorithm (58) as described previously (29). In some cases (e.g., actin-activated solution ATPase and some control simulations; Figs. S1 and S11), predictions were obtained by solving differential equations in state probabilities (29). Because experiments on skinned muscle fibers were conducted at 5°C, we also performed simple analysis based on changes in key parameters to values corresponding to those at 5°C (Table S3).

More methodological details, including details about the modeling, are given in the Supporting Materials and Methods.

## Results

**Figure 2:**
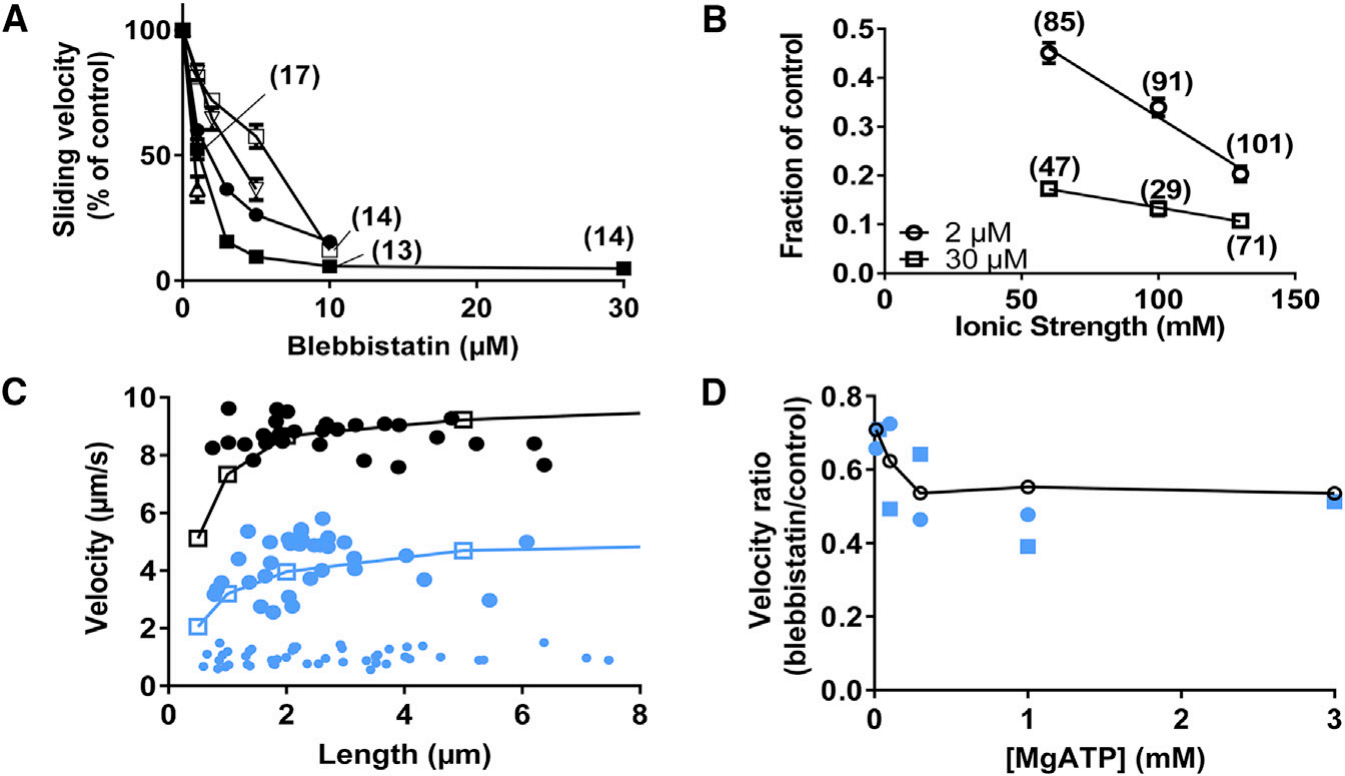
Effects of blebbistatin on sliding velocities in the in vitro motility assay. (*A*) Concentration-response curve for effect of blebbistatin on sliding velocity at 130 mM ionic strength (1 mM MgATP) is shown using HMM with dephosphorylated regulatory light chains (dP-HMM). Each data point represents two to five flow cells and more than 20 filaments unless indicated in parentheses. Data from three different blebbistatin batches with mixed enantiomers are illustrated by different symbols, and two different dP-HMM preparations are illustrated by filled and open symbols, respectively. Two experiments using pure S-(−) blebbistatin are illustrated by open triangles. The sliding velocity in the control solution (0 *μ*M blebbistatin) varied between 9.8 and 11.8 *μ*m/s in the different experiments. (*B*) Blebbistatin effects on sliding velocity at different ionic strengths (1 mM MgATP) are shown. Fractional velocity at either 2 *μ*M (*circles*) or 30 *μ*M (*squares*) blebbistatin is shown, where velocity data are normalized to the velocity at the respective ionic strength in the absence of blebbistatin (control). In the absence of blebbistatin, the mean velocity was constant with changes in ionic strengths to ± 3%. (*C*) Velocity versus filament length is shown in the absence of blebbistatin (*black*) or in the presence of 10 *μ*M blebbistatin (*small bright symbols*) or a blebbistatin concentration (5 *μ*M; *big bright symbols*) approximately reducing the velocity to half. Filled symbols show experimental data. Open symbols connected by lines show simulated data assuming three independent myosin-binding sites per 36 nm of the actin filament. The blebbistatin effect was modeled using a model in which k_P+_' was reduced from 1000 to 1.5 s^−1^, as illustrated in greater detail in Fig. 4 below (1 mM MgATP; ionic strength 130 mM). (*D*) Sliding velocity vs. [MgATP] in the presence of 1 *μ*M blebbistatin is shown as fraction of velocity in the absence of the blebbistatin (*bright symbols*). Data points from experiment with mixed blebbistatin enantiomer (*filled circles*) or pure S-(−) blebbistatin (*filled squares*) are shown. Different myosin preparations were used for these two experiments. The number of filaments for each data point was >20 in all cases. Open symbols connected by line represent blebbistatin effects modeled using a model in which k_P+_' was reduced from 1000 to 1.5 s^−1^. See further details in Fig. 4. Temperature was 27.6–30.5°C (constant to within 1.0°C in a given experiment). Error bars had 95% confidence intervals. To see this figure in color, go online.

Blebbistatin reduced the sliding velocity in the in vitro motility assay in a concentration-dependent manner (Fig. 2 *A*). The half-maximal inhibition occurred in the range of ∼1–5 *μ*M blebbistatin for three different batches of HMM (three different myosin preparations) and three different batches of blebbistatin with a mixture of (+) and (−) enantiomers. In two experiments, we also tested pure S-(−) blebbistatin, generally claimed to be the active enantiomer (40). The half-inhibitory concentration in this case was <1 *μ*M for one HMM batch and ∼3 *μ*M for the other (Fig. 2 *A*). The sliding velocity plateaued at a nonzero value at blebbistatin concentrations higher than 7–10 *μ*M. In tests below, we titrated the concentration of each specific blebbistatin batch to achieve ∼50% blebbistatin-induced reduction in velocity compared to the control. Two of the three HMM preparations in Fig. 2 had dephosphorylated RLCs (dephosphorylated (dP)-HMM), as verified by Urea gels (Fig. S2). The third HMM batch was prepared to give dP-HMM, but the phosphorylation status was not verified in a gel. Importantly, however, partial phosphorylation of the RLCs did not alter the blebbistatin effects (Figs. S2–S4).

Biochemical assays suggest that blebbistatin inhibits a transition that traps myosin heads in a weakly actin-attached state (41). A blebbistatin-induced reduction in force is a self-evident consequence of such a mechanism. However, a reduction in maximal sliding velocity is not because velocity is believed to be governed primarily by the cross-bridge detachment rate at the end of the power stroke (59, 60, 61). Nevertheless, one possibility to account for lowered velocity as a consequence of increased population of weakly bound states is if blebbistatin enhances actin affinity in these states, leading to increased friction upon relative sliding of actin and myosin (28, 62). If such a mechanism is at work, the effect of blebbistatin on sliding velocity would be increased by lowering ionic strength because the actin affinity of weak-binding states is approximately doubled for a 20 mM reduction in ionic strength (63). However, we found (Fig. 2 *B*) a reduced effect of blebbistatin on velocity when ionic strength was lowered in the range from 130 to 60 mM, arguing against the relevance of frictional forces. This idea is consistent with the lack of change in velocity when the ionic strength was varied in the range of 60–130 mM in the absence of blebbistatin (Fig. S5). The attenuation of the blebbistatin effect at reduced ionic strength is briefly considered in Supporting Materials and Methods.

One other possibility to account for blebbistatin-induced reduction in velocity on the basis of reduced attachment of cross-bridges into force-producing states is if the velocity reduction primarily reflects the behavior of the shortest actin filaments in the in vitro motility assay. Such filaments are propelled by a few myosin motors also in the absence of blebbistatin (29, 64), and a further reduction in this number by reduced attachment rate would severely reduce the propulsion speed. However, our results show blebbistatin effects on sliding velocity that are virtually independent of filament length (Fig. 2 *C*). There was no tendency for reduced effect of the compound even for the longest filaments observed. Similar results were found whether we used motility assays without (Fig. 2 *C*) or with (Fig. S4) blocking actin to largely eliminate the effects of rigor-like heads (cf. (55)). A lowered HMM density on the surface was achieved in one experiment by reduced HMM incubation concentration from 120 to 30 *μ*g/mL (Fig. S6). Under these conditions, the blebbistatin effect on velocity was slightly higher for the shortest filaments (1 *μ*m) compared to the longest filaments observed. However, overall the reduced HMM density did not enhance the blebbistatin effect when all filament lengths were considered. The findings argue further against ideas that lowering the cross-bridge attachment rate plays any appreciable role in the effect of blebbistatin on velocity.

For further insight into the mechanisms underlying the blebbistatin effects, we studied the relationship between [MgATP] and velocity because this relationship is influenced by kinetic parameters of the cross-bridge cycle as well as by the myosin step length (65). The main finding is that the blebbistatin effect on velocity was lower at the lowest MgATP concentrations (<0.1 mM) studied (Fig. 2 *D*). This effect was associated with reduced MgATP concentration (K_M_^v^) for half-maximal velocity in the presence of 1 *μ*M blebbistatin (Fig. S7). Biochemical kinetics suggests limited effects of blebbistatin on cross-bridge detachment rate at the end of the power stroke (41). We therefore conclude that blebbistatin causes accumulation of cross-bridges in a pre-power-stroke attached state that acts as a brake on cross-bridge sliding. Such a mechanism lowers K_M_^v^ because it reduces velocity particularly at high [MgATP] when there are very few braking cross-bridges in the MgATP-free Actin-Myosin (AM) (rigor) state. The latter cross-bridge state, on the other hand, has a dominating role in limiting velocity at low [MgATP], both in the presence and absence of blebbistatin, explaining the low effect of blebbistatin in this MgATP concentration range.

**Figure 3:**
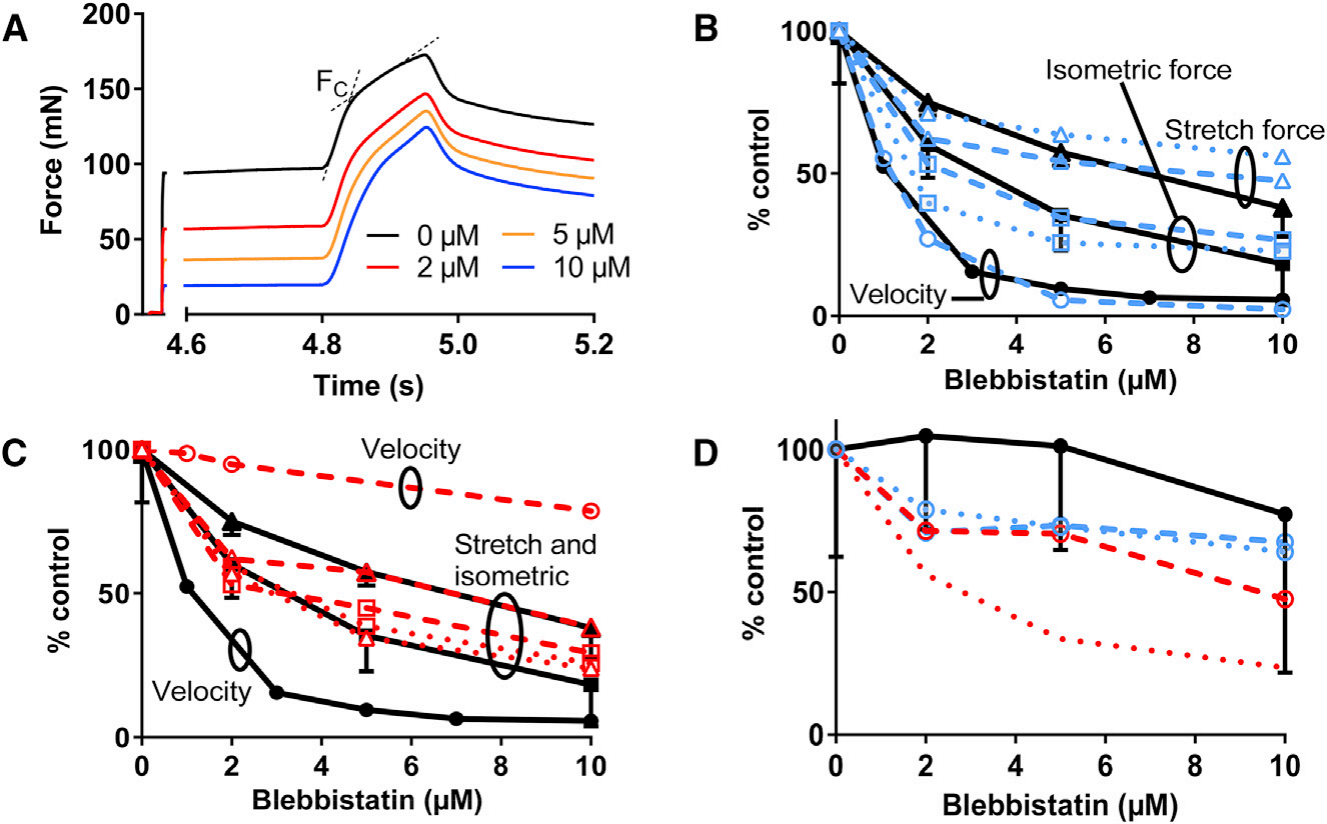
Effects of different concentrations of blebbistatin on contractile parameters in experiments and simulations. (*A*) Force records show the effects of blebbistatin on isometric force and the force response to stretch of fast skinned muscle fibers of rabbit psoas muscle. Stretch ramps at velocity of 2.5 fiber lengths/s (2500 nm/half-sarcomere/s) were imposed between times of 4.8 and 4.95 s during otherwise steady-state isometric contraction. The records were obtained in the presence of 0–10 *μ*M blebbistatin. The critical force during stretch, F_c_, was estimated as indicated by crossing dashed lines. (*B*) Concentration-effect curves for experiments (*filled symbols, full black lines*) were compared to the model (*blue*) assuming blebbistatin-induced reduction of rate constant (k_P+_') for the transition from the AM^∗^'DP to the AM^∗^DP state from 1000 to 1.5 s^−1^. Parameter values are either assumed to apply at 25–30°C (Tables S1 and S2, *open symbols, dashed lines*) or 5°C (Table S3, *dotted lines*). (*C*) Concentration-effect curves for experiments (*filled symbols, full lines*) were compared to the model assuming reduction of ΔG_on_ (Eq. S2) from 0.7 to −4 k_B_T (*red*). Other parameter values for 25–30°C (Tables S1 and S2, *open symbols, dashed lines*) or 5°C (Table S3, *dotted lines*) are shown. (*D*) Force enhancement during stretch (Fc-isometric force) is plotted versus blebbistatin concentration with color and symbol coding as in (*B*) and (*C*), i.e., lower data sets (*red*), model assuming reduction in *Δ*G_on_, and middle data sets (*blue*), model assuming reduction in k_P+_'. All data are normalized to control values. The experimental data for sliding velocity (*circles*) are from Fig. 1*A* (blebbistatin batch with highest activity). Error bars, 95% confidence interval. *N* = 16 muscle fibers are tested for each blebbistatin concentration. Blebbistatin affinity (1 *μ*M^−1^) in the simulations is shown according to measurements in (41) using rabbit fast muscle myosin. For absolute values of forces and velocity in simulations, see Figs. 2, 4, and S10 (high temperature).

We also investigated the effects of blebbistatin on maximal isometric force and on the resistance to stretch in lengthening contractions of skinned rabbit psoas muscle cells (Fig. 3 *A*). The experiments were performed at 5°C, a temperature with little blebbistatin-induced myosin head ordering on the thick filament backbone, similar to the effects of RLC phosphorylation (66). We found a concentration-dependent reduction in isometric force and force during stretch, expanding previous findings (51) with 5 *μ*M blebbistatin. The typical phases of the tension response to stretch during active contraction were maintained at all blebbistatin concentrations tested. These phases include a fast increase in force concomitant with half-sarcomere extension of a few nanometers, and a subsequent slow increase (67, 68, 69). The first phase is commonly attributed to the elastic behavior of the cross-bridges, and the second phase to changes in the occupational fraction of cross-bridges in the pre- and postpower-stroke states (51, 69, 70). The second phase may also reflect contribution of parallel elastic elements, e.g., those attributed to the stretching of titin (71). The transition between the two phases is marked by a change in slope of the force rise at a critical force (F_c_), commonly associated with the mechanical detachment of cross-bridges from actin.

In Fig. 3, *B* and *C* the sliding velocity, the isometric force, and F_c_ are all plotted against the blebbistatin concentration as fractions of the values in the absence of the compound. These data show that the relative magnitude of the blebbistatin-induced reduction varies in the following order: effect on velocity > effect on isometric force > effect on F_c_. The experimental data in Fig. 3 are shown together with simulation data referring to different conditions (see further below). The experimental results in Fig. 3, *A* and *D* show that the force enhancement during stretch was little changed by blebbistatin, more consistent with lower blebbistatin-induced reduction of the critical force during stretch than of the isometric force (Fig. 3, *B* and *C*). This result fits with the idea that actomyosin cross-bridges with blebbistatin contribute to the force response during stretch in our experiments rather than being parked on the thick filament backbone. This can be inferred by comparing to the effects of varied Ca^2+^ concentrations in which the force during stretch was approximately proportional to the isometric force (51). The results are consistent with a proportionally larger reduction in isometric force by blebbistatin than in the number of attached cross-bridges resisting lengthening.

### Model predictions of experimental results

An ideal model for actomyosin-based force generation should be as simple as possible while accounting for contractile phenomena both under normal physiological conditions and in the presence of mutations and/or modifying chemical compounds. A suitable model for initial analysis, as motivated in the Discussion, is that in (29) (Fig. S8). However, this model (29) could not predict the very substantial inhibiting effect of blebbistatin on actomyosin ATPase while simultaneously accounting for similar reduction in sliding velocity for short and long actin filaments in the in vitro motility assay. The blebbistatin-induced reduction of the ATPase activity in the model could only be achieved by lowering the rate constant for the transition between weakly and strongly bound actomyosin cross-bridge states (k_on_(x) in Fig. S8 *A*). However, this mechanism appreciably lowered the sliding velocity only for short and not for long actin filaments. The alternative mechanism, which shows that blebbistatin acts primarily by inhibiting the power stroke (“the Huxley and Simmons transition” (30) governed by K_HL_ in Fig. S8 *A*), does not explain the blebbistatin effect on the actomyosin ATPase. Neither the combination of the two mechanisms is successful in accounting for the blebbistatin effects (Fig. S8, *B* and *C*). Thus, in contrast to the experimental findings, an appreciable filament length dependence of the blebbistatin effect on velocity was predicted, and the K_ATPase_ for the actin-activated ATPase activity would be increased by blebbistatin (Fig. S8 *B*) rather than decreased, as found experimentally (41). This suggests that a more complex model is required.

**Figure 4:**
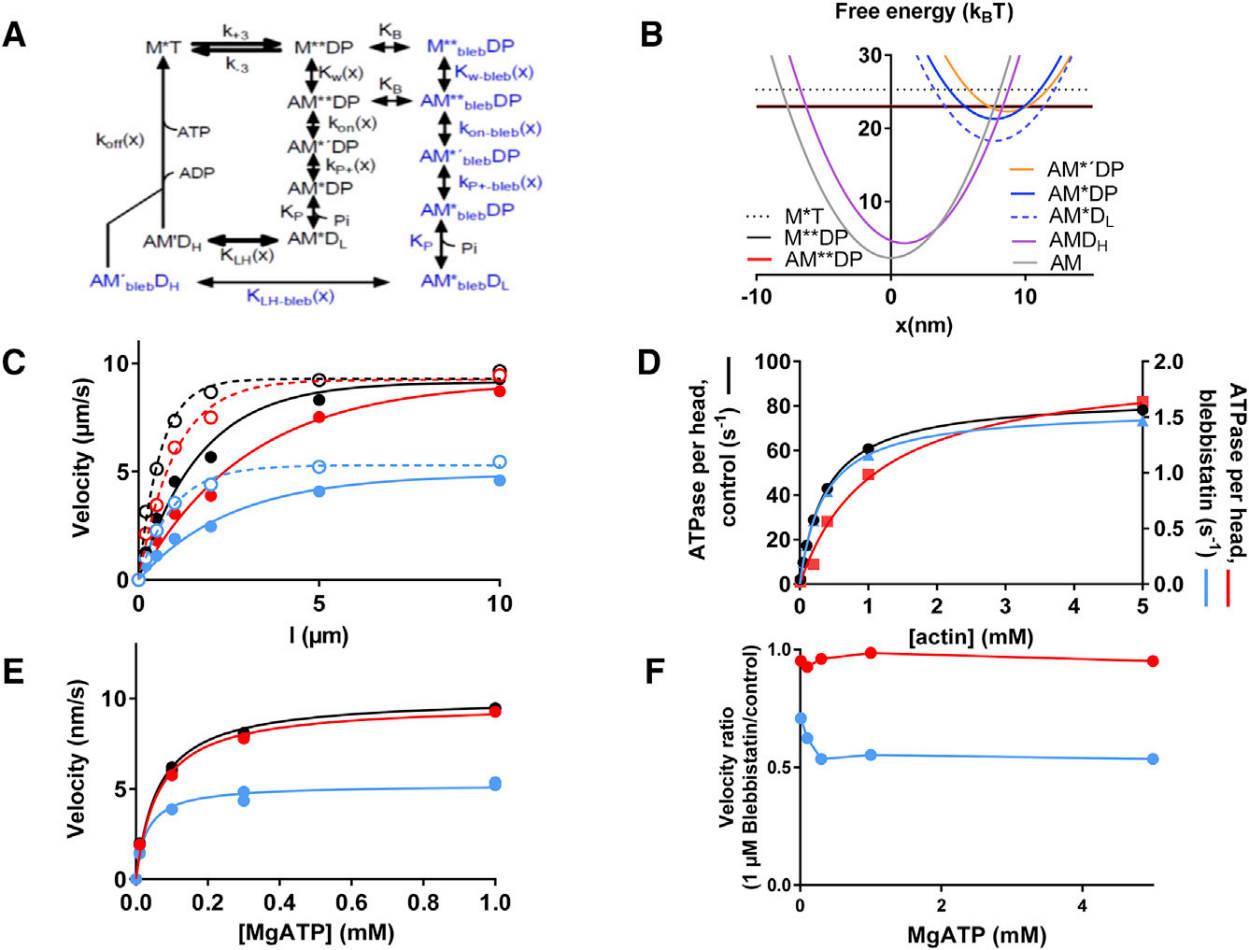
Modeling of blebbistatin effects. (*A*) Kinetic scheme of model states shows myosin (M) free or bound to actin (*A*) with ATP (T), ADP (D), and/or inorganic phosphate (P, Pi) at the active site. Several states exist also in blebbistatin-bound form (*blue*; subscript “bleb”). Rate constants or rate functions are indicated by the generic form k_i_(x) and equilibrium constant by K_i_. Only forward rates are indicated. (*B*) Free-energy diagrams illustrate the dependence on the variable *x* of the free energy of cross-bridges in the states without blebbistatin. Here, *x* is the distance between the nearest actin site and a reference point on the myosin filament with x = 0 nm at the free-energy minimum of the AM state. (*C*) Simulated velocity length plots in the in vitro motility assay with standard conditions (*black*) and 1 *μ*M blebbistatin (*colored circles*) are shown, assuming changes in parameter values corresponding to different hypotheses for blebbistatin effects. Simulations for 1 *μ*M blebbistatin assumed either one (*filled symbols* and *full lines*) or three (*open symbols* and *dashed lines*) myosin-binding sites for actin per 36 nm half-repeat of the actin filament. Conditions tested included the following: 1) ΔG_on_ reduced from 0.7 to −4 k_B_T (*red*; reduction in k_on_(x); Eq. S2) and 2) k_P+_' reduced from 1000 to 1.5 s^−1^ (*blue*; Eqs. S4 and S5). (*D*) Simulated relationships are shown between actomyosin ATPase rate and actin concentration in solution. Color coding corresponds to that in (*C*), but a saturating blebbistatin concentration is assumed when the simulations are performed with the compound believed to be present. Curves represent fits of hyperbolic functions to the data with Michaelis-Menten constants V_max_ = 84.2 ± 0.2 s^−1^ (mean ± 95% confidence interval) and K_ATPase_ = 0.386 ± 0.001 mM under control conditions (*black*; *left axis*) and V_max_ = 1.98 ± 0.20 s^−1^ and K_ATPase_ = 1.09 ± 0.33 mM (*red*; *right axis*) or V_Max_ = 1.57 ± 0.02 s^−1^ and K_ATPase_ = 0.355 ± 0.002 mM (*blue*; *right axis*). (*E*) Sliding velocity vs. [MgATP] is simulated using parameter values according to color coding in (*C*) and (*D*). Curves represent fits of hyperbolic functions to the data (including points at 5 mM MgATP; data not shown) with Michaelis-Menten constants V_Max_ = 10.1 ± 0.4 *μ*m/s and K_M_^v^ = 0.062 ± 0.015 mM under control conditions (*black*) and V_Max_ = 5.22 ± 0.34 *μ*m/s and K_M_^v^ = 0.031 ± 0.010 mM assuming reduced k_P+_' (*blue*) or k_on_(x) (*red*) in the presence of 1 *μ*M blebbistatin. Filaments are assumed to be 20 *μ*m long. Note the slightly low V_Max_ and the low K_M_^v^ values compared to the experimental data in Fig. S7 would be increased by assuming nonlinear cross-bridge elasticity (55), but this was not further pursued here. (*F*) Simulated sliding velocity vs. [MgATP] is shown in the presence of 1 *μ*M blebbistatin as a fraction of velocity in the absence of the blebbistatin using parameter values according to color coding in other panels.

The model in Fig. S8 (29) was therefore expanded (Figs. S9 and 4) by incorporating recent ideas for the mechanism of phosphate release (19). More specifically, the ideas of Llinas et al. (19) were integrated into the model (29) by addition (Fig. 4, *A* and *B*; schematic in Fig. 1 *C*) of 1) a weakly but stereospecifically bound pre-power-stroke state (AM^∗^'DP) and 2) a phosphate release state (AM^∗^DP) (Fig. 4 *A*). These states precede the main force-generating transition ((30); equilibrium constant K_LH_) that is responsible for the fast force recovery in response to length steps. The expanded model, appropriately reduced as described and motivated in Fig. S9, maintains the capacity of the original model (29) to account for several experimental findings in the absence of blebbistatin. This includes the shape of the force-velocity relationship and the relationships between [Pi] on the one hand and the magnitudes of the maximal sliding velocity and the maximal isometric force on the other (Fig. S1, *A* and *B*). Furthermore, the model is consistent with recent data ((18); see Fig. S1, *C* and *D*) suggesting that both phosphate release and cross-bridge attachment into force-generating states are rate-limited by the same transition(s) (k_on_(x); Fig. 4 *A*). Also, the actomyosin ATPase is primarily rate limited by k_on_(x) in the model, but it is modulated (Legend, Fig. S1) by the rate constant k_+3_ (Fig. 4 *A*). Here, k_on_(x) is the rate function (denoted attachment rate constant in the following discussion) for the transition from the nonstereospecific weakly bound actomyosin state AM^∗∗^DP to the stereospecific weakly bound state AM^∗^'DP.

The blebbistatin binding to and dissociation from the myosin head is treated in a simplified way as rapid equilibrium binding (K_bind_ = 1 *μ*M^−1^) to the M^∗∗^DP state and irreversible dissociation from the AM'D_H_ state. These simplifications account well for the blebbistatin effects on studied mechanical parameters (see below) and also accord with more than an order of magnitude increase (41) in the effective equilibrium between the M^∗∗^DP states (the M^∗∗^DP + M^∗∗^DP_bleb_ states) and the M^∗^T state. The effective equilibrium constant increases from ∼13 to ∼995 without any change in K_3_ because of the greatly increased population of the M^∗∗^DP_bleb_ state upon addition of blebbistatin.

We next tested the hypothesis that the blebbistatin effects are explained by reduced value of k_on_(x) in the expanded scheme in Fig. 4 *A*. A reduced magnitude of this rate function is sufficient to account for the blebbistatin-induced reduction of V_max_ of the actomyosin ATPase (41, 43). Additionally, it correctly predicts reduced isometric force and reduction in velocity for 1-*μ*m-long filaments. However, the predicted velocity-reduction for the longest filaments (5–20 *μ*m) studied in the model is appreciably smaller than that observed experimentally (Figs. 2 *C* and 4 *C*). Importantly, this conclusion is further strengthened under the assumption of three binding sites for myosin per helical half-repeat of actin (Fig. 2 *C*). Furthermore, the K_ATPase_ ([actin] for half-maximal steady-state ATPase activity) is increased (Fig. 4 *D*) in contrast to the small decrease found experimentally (41). These findings, together with proportionally rather similar blebbistatin-induced reduction in isometric force and force enhancement during stretch in simulations (Fig. 3 *D*; Fig. S10 *B*), argue strongly against the idea that blebbistatin acts by lowering k_on_(x). Indeed, the finding that a reduction of k_on_(x) alone cannot account for the blebbistatin effect is entirely consistent with the findings based on the simpler model (29) (Fig. S8). This follows because the latter model is virtually identical to the expanded model (Fig. 4 *A*) unless the rate function k_P+_(x) is appreciably reduced below its standard value (1000 s^−1^). The latter high value was chosen based on control simulations suggesting that k_P+_(x) must be higher than 500 s^−1^ in the absence of blebbistatin to explain the high velocity (∼10 *μ*m/s) under these conditions. Thus, under physiological conditions, the AM^∗^'DP state would be negligibly populated, and its existence is revealed by functional studies only after adding inhibiting compounds such as blebbistatin.

Alternative hypotheses that were initially considered include the effects of blebbistatin on the phosphate affinity (Kp) or the main force-generating transition (30) between the AM^∗^D_L_ and the AM'D_H_ state. However, these ideas are readily falsified without further analysis because neither of them accounts for the hallmark finding that blebbistatin appreciably reduces V_max_ of the actomyosin ATPase. It may, however, be worth mentioning that isolated inhibition of the AM^∗^D_L_-AM'D_H_ transition in the model would markedly reduce both the sliding velocity and the maximal force, whereas altered Kp has no effect on sliding velocity.

Finally, we tested the hypothesis that blebbistatin slows the transition from the AM^∗^'DP to the AM^∗^DP state (reduction in k_P+_(x); Fig. 4 *A*), i.e., the transition from a stereospecifically attached pre-power-stroke state to the phosphate release state (19). To quantitatively account for the reduced V_max_ of the actomyosin ATPase, the transition rate has to be reduced 650-fold. Good fits are obtained whether both the backward and forward reactions are reduced equally (i.e., increased activation energies for these transitions) or if only the forward rate constant is slowed by blebbistatin. The latter change would be consistent with higher free energy of the states after the AM^∗^'DP state. Importantly, the magnitude of the reduction in k_P+_(x), necessary to account for the blebbistatin-induced reduction in the maximal ATPase activity, quantitatively predicts other experimental findings by us and others without the need for further assumptions. The findings that are accounted for include 1) reduction in the maximal sliding velocity (Fig. 4 *C*), 2) reduction in the maximal isometric force (Fig. 3 *B*), 3) reduction in the K_ATPase_ (Fig. 4 *D*), 4) reduction of K_M_^v^ (Fig. 4 *E*), and 5) the lack of noticeable blebbistatin-induced change in the shape of the velocity-length plot (Fig. 4 *C*).

The favored mechanism (Fig. 3, *B* and *D*), compared to the mechanism assuming reduced attachment rate constant (k_on_(x) (Fig. 3, *C* and *D*), also provides quantitatively better reproduction of the blebbistatin-induced effects on force during stretch of active muscle (Fig. 3 *A*). This also applied if we modified the parameter values (Table S3) to be more consistent with the low temperature (5°C) of the experiment (Fig. 3, *B*–*D*). Finally, a shift of the rate-limiting transition to that between the AM^∗^'DP to the AM^∗^DP state (k_P+_(x)) would reduce the rate of rise of isometric force. However, the predicted reduction in this rate is appreciably lower (∼5-fold; Fig. S10) than the effect on the V_max_ of the actomyosin ATPase (∼50-fold reduction). We tested whether the model predictions may be further improved by introducing blebbistatin-induced inhibition of the power stroke (44). Adding this mechanism to reduced value of k_P+_ in response to blebbistatin improves the fit to the stretch response (Fig. S10 *D*) and to some other experimental results (Table S4).

## Discussion

Our central result is that a mechanokinetic model incorporating recent ideas for the phosphate release and the force-generating process (18, 19, 25, 29) accounts for a substantial set of experimental data both in the presence (Figs. 1, 2, and 3) and absence (Figs. 1, 2, 3, S1, and S2) of blebbistatin. The final model (Fig. 4 *A*) was developed by expanding a recent simpler model (29). The latter model (29), in turn, was selected for initial tests because all states are independently supported by biochemical, physiological, or structural studies (19, 29), allowing direct connections between biochemistry, structure, and contractile function. This model is defined by parameter values obtained under coherent conditions with regard to temperature, ionic strength, muscle type, etc. ((29); Tables S1 and S2). Furthermore, the elastic properties and free-energy profiles are consistent with single-molecule mechanics (72, 73) but have been fine-tuned to fit force-velocity data (29, 33, 55, 74) of fast vertebrate skeletal muscle. Finally, the model (29) accounts for findings ranging from single-molecule experiments over experiments on small myosin ensembles to the large ordered ensembles in muscle cells.

With regard to the sequence of events defining the initially tested model in Fig. S8 *A* (29), the rate-limiting steps, the two-step Pi release, and the temporal relationship between these events as well as the main force-generating structural change all have independent support in the literature. First, there is evidence that the same transition limits actomyosin ATPase activity and force generation ((28); see also (29)). In addition, recent experimental results on cardiac myofibrils suggest that the same transition is rate limiting for the tension changes in response to jumps in phosphate and calcium concentrations as well as in response to slackening-restretch procedures (18). This suggests that both force generation from the detached state and force changes in response to jumps in phosphate concentration are rate limited by the transition (k_on_(x)) that also rate-limits actomyosin ATPase. Admittedly, direct evidence for identical rate limitation for Pi transients and for force generation from the relaxed state has not been provided for fast rabbit skeletal muscle. However, the sarcomere nonuniformities that have central roles in the studies of cardiac myofibrils by Stehle (18) bear strong resemblance to those underlying the two phases of relaxation after a tetanus of fast skeletal muscle fibers (75). This demonstrates the existence of closely related mechanisms for tension relaxation in both preparations. Furthermore, two phases of tension relaxation have also been observed in skeletal muscle fibers upon sudden increase in Pi concentration but without the possibility for detailed analysis of the first phase due to its brief duration (9). These findings, taken together, provide arguments suggesting that the recent observations in cardiac myofibrils (18) can be extrapolated to skeletal muscle fibers. Finally, an identical rate-limiting step for Pi transients and force generation is consistent with the structural data (19, 20).

A second important feature of the model in Fig. S8 *A* (29) is that Pi release is a two-step process with one slow isomerization in series with rapid equilibrium Pi release (9, 12, 17). In two recent models (25, 29), the slow step has been assumed to correspond to the transition between the weak-binding and first strong-binding actomyosin state just before Pi release. In this connection, we agree with arguments put forward by Smith (25) against a slow Pi release step due to the severe velocity reduction it would produce. We also agree with arguments (25) based on energetics and effects of [Pi] on tension, suggesting that Pi release occurs before the force-generating structural change. The emerging picture with slow cross-bridge attachment, followed by fast Pi-release and the force-generating structural transition in sequence, is also fully consistent with structural findings (19, 20). One of the latter studies ((19); see further (25)) also provides evidence for an intermediate phosphate-binding site in the Pi-release tunnel that causes a delay before the released phosphate appears in solution. This can account for recent results from myosin II (15) and myosin V (26), which have suggested that Pi release occurs after the force-generating structural state. The fast force generation (K_HL_) after the Pi-release in the model (29) corresponds directly to that suggested by Huxley and Simmons (30) to be responsible for the rate of the fast force recovery after length steps. This idea fits with the strain dependence of the transition governed by K_LH_ in the model. The strain dependence follows from a shift along the *x* axis of the free-energy diagrams for the AM^∗^D_L_ and AM’D_H_ states relative to each other (Fig. 4 *B*). The proposed sequence of events is also consistent with the lack of [Pi] effect on the tension recovery process after a shortening step. The situation for stretches is more complex. A rapid stretch would thus increasingly populate the AM^∗^D_L_ state at x-values close to its free-energy minimum and close to the x-value at which transitions back and forth between the AM^∗^D_L_ state and the M^∗∗^DP state are most likely.

We take the above arguments as solid foundations for using the model (29) as a starting point for evaluating the blebbistastin effects. However, as shown above, neither that model nor, for example, that in (25), could account for the data with blebbistatin unless we also added states and transitions similar to those proposed by Llinas et al. (19). After the latter modification, the following view emerges: the initial transition from weakly to strongly bound pre-power-stroke states is rate limiting under normal conditions, but blebbistatin shifts the rate-limiting step to the subsequent transition between a pre-power-stroke state and a Pi release state (19). Thus, blebbistatin gives functional significance to the otherwise transient pre-power-stroke state proposed in (19). In the Pi release state, there is rapid equilibrium Pi release from the active site without any change in position of the lever arm. This is consistent with the same x-values for the free-energy minima of the AM^∗^DP and the AM^∗^D_L_ states. After phosphate release, a rapid force-generating structural change with a swing of the lever arm (the “power stroke” (30)) follows (between the AM^∗^D_L_ and the AM’D_H_ states), as indicated schematically by model 2 in Fig. 1 *C*.

Some issues regarding the quantitative analysis of the two major hypotheses (effect on k_P+_(x) or k_on_(x)) deserve comments. First, to summarize the principle of the analysis, we do not explore the entire parameter space, but instead, we first fix all parameters at literature values to simulate results in the absence of blebbistatin. Then, the magnitude of the blebbistatin effect on k_P+_(x) or k_on_(x) is inferred from the effect of saturating blebbistatin concentration on the actin-activated ATPase activity. Next, we performed Monte Carlo simulations to test if either of these changes also account for other blebbistatin effects observed by us and others.

This analysis, using fixed parameter values, has both advantages and disadvantages compared to methods relying on nonlinear regression, e.g., downhill simplex minimization procedures (76) in which the entire parameter space is explored. Of course, nonlinear regression would not be practically feasible in this case using slow Monte Carlo simulations. However, assuming that such an approach would be possible, it would nevertheless be problematic. Thus, with this number of parameter values, multiple local minima may occur in the error function with uncertainties in the best fit values. On the other hand, one may argue that fixing the parameter space may also introduce errors, e.g., if one or several of the fixed parameter values are erroneous, a worst-case scenario may be that the best fit (in the least-square sense) is obtained for a false hypothesis. However, in this case, this would seem to require appreciable uncertainties in the parameter values because the model assuming reduced k_P+_(x) gives a sum of squared errors that is more than one order of magnitude lower (Table S4) than that of the model assuming reduced k_on_(x). Also, importantly, the differences in the sum of squared errors between the two major models (Table S4) originate in substantial differences in the predictions of several individual variables (Fig. 4). Furthermore, the idea of appreciable uncertainties in the parameter values is in conflict with the very good fits to a range of independent and complex experimental data using the parameter values in Tables S1 and S2. This includes the force-velocity relationship and the actomyosin ATPase without blebbistatin (Figs. S1 and 4 in main article). Some of the parameter values, such as those defining the free-energy diagrams (cross-bridge stiffness, level, and *x*-position of free-energy minima) are also linked in the sense that change of one require changes of others to give reasonable force-velocity relationships. This argues against independent searches of the entire parameter space. Additionally, the existence of linked parameter values contributes to greater certainty in the values that we have actually used. Thus, although the latter define the free-energy profiles in a way that is consistent with recent optical tweezers data (72, 73), they have also been fine-tuned to each other (including subnanometer and single k_B_T changes in position and levels of the free-energy minima) in several studies (29, 33, 55, 74) to account for the force-velocity relationship. The above arguments suggest minimal risks of error in selecting between the two major models for the blebbistatin effects due to uncertainties in the parameter values used (Tables S1 and S2). This view is supported by the analysis in Fig. S11 and Table S5. Here, we tested the model predictions for the two major hypotheses above using different sets of parameter values, in which each value was randomly selected to be either 25% higher or 25% lower than the literature value in Tables S1 and S2. First, it is of interest to note that only 2 out of 10 sets of alternative parameter values selected in this way gave reasonable fits to the experimental force-velocity data. However, importantly, for all of six tested sets of parameter values (including those fitting the force-velocity relationship), the blebbistatin results were more faithfully reproduced by a reduction in kp+' than by a reduction in k_on_(x).

Both the AM^∗^'DP and the AM^∗^DP states are essential in the model in Fig. 4, *A* and *B* to account for the blebbistatin effects and most likely for the effects of other drugs such as 2,3-butanedione 2-monoxime (BDM) and N-benzyl-p-toluene sulphonamide (BTS) (35, 36). Additional challenging tests would ascertain whether the model, in detail, predicts effects of altered temperature (77), effects of other drugs (6, 38), and various mutation effects. It should also be tested if the model can account for phenomena previously found to require states and transitions without independent evidence in the literature (14, 27).

An important basis for interpreting the effects of blebbistatin on force, velocity, and actomyosin ATPase within one theoretical framework and one mechanism was the finding that the blebbistatin effects on velocity in the in vitro motility assay (in contrast to the situation in cells (52)) do not require phosphorylation of the RLCs. The results support the view that the lack of blebbistatin effects in the absence of RLC phosphorylation in cells is due to blebbistatin-induced enhancement of myosin head interactions with the thick filament backbones (absent in the in vitro motility assay). These interactions prevent the myosin heads with bound blebbistatin to interact with actin. When such interactions are eliminated, however, as in these in vitro motility assays, it is reasonable to view the reduction in isometric force, actomyosin ATPase rate, and sliding velocity as different facets of one specific blebbistatin effect on actomyosin function.

A remaining uncertainty (29) in the expanded model (Fig. 4) is whether the cross-bridge elasticity is linear or nonlinear (55, 72). Our assumption of linear, rather than nonlinear, cross-bridge elasticity could explain (29, 55) the lower K_M_^v^ value (for sliding velocity vs. [MgATP]) in simulations; Fig. 4 *E*) than that in experiments (Fig. 2 *D*). Also, the exact mechanism underlying the response of active muscle to stretch is uncertain (78, 79). Therefore, this treatment in this regard is necessarily somewhat tentative.

One source of complexity compared to previous models (41) is that the blebbistatin-inhibited step is different from the step that normally rate-limits the actomyosin ATP turnover cycle. Additionally, this transition occurs between two stereospecifically attached states. This means that when blebbistatin is bound to myosin, the transition between these states (governed by k_P+_(x); very fast in the absence of blebbistatin) is so extensively inhibited that it becomes the new rate limiting step for the cycle instead of k_on_(x). It is important to note in this connection that we do not propose an alternative pathway for Pi release upon blebbistatin binding. Instead, we suggest that the rate of a specific transition in the normal path (19, 25, 29) is appreciably reduced.

The idea that blebbistatin shifts the rate-limiting step to a transition between attached states implies increased fraction of actin-bound myosin heads in the presence of MgATP. This idea is consistent with the blebbistatin-induced reduction of the K_ATPase_ for the actin-activated ATPase and with the finding that blebbistatin-induced complete motility inhibition in the in vitro motility assay did not cause filament detachment from the myosin-coated surface (80). In contrast, no clearly noticeable blebbistatin-induced change in myosin head binding to actin in the presence of *μ*M MgATP could be deduced from pyrene-actin fluorescence or light-scattering data. This led Kovacs et al. (41) to conclude that also in the presence of blebbistatin, the myosin heads are predominantly not strongly bound to actin during steady-state ATP hydrolysis. Besides, a 50% blebbistatin-induced reduction in myosin binding to actin was observed in a cosedimentation assay in the presence of 10 mM MgATP, ∼1 mM free Mg^2+^, and 0 mM monovalent salt (41). These findings imply complexities in the experimental results (e.g., effects of low ionic strength on blebbistatin effects on actin-binding) or possibly in the mechanism of action of blebbistatin. These complexities deserve further study. For instance, it would be of interest to investigate whether blebbistatin stabilized state(s) could be detected in single-molecule studies such as ultrafast optical tweezers based force spectroscopy (81). In this connection, it is of interest to note that one of the pre-power-stroke cross-bridge states found by Capitanio et al. (81) using fast myosin II has properties reminiscent of the AM^∗^'DP state suggested to be increasingly populated due to the blebbistatin effect.

Our model agrees with the generally held view that blebbistatin appreciably reduces the population of strongly bound (Actin-Mysoin and Actin-Myosin-Adenosine diphosphate (AMD)) states. However, we found no noticeable change in the shape of the velocity length plot. This suggests that the blebbistatin effects cannot be unambiguously described using the duty ratio concept (64, 82). Thus, whereas the duty ratio is defined as the fraction of the cycle time spent by the myosin heads in strongly bound force-producing (AM and AMD) states (64, 82), its numerical value is generally estimated from the shape of the plot of velocity versus the number of available myosin heads (29, 39, 64, 83). Such plots in our study suggest virtually unchanged duty ratios in the presence of blebbistatin, whereas all other experimental data point to fewer heads in the AM and AMD states, consistent with the reduced duty ratio.

The need to assume a changed rate-limiting step of the actomyosin ATPase to account for the simultaneous blebbistatin effects on ATPase and sliding velocity is consistent with evidence that these two variables are governed by different transitions in the absence of blebbistatin. Thus, in vitro sliding velocity and the unloaded shortening velocity in muscle on the one hand and V_max_ of the actomyosin ATPase on the other exhibit different temperature dependence (60) for several different myosin isoforms (61). Additionally, a genetic modification of the myosin motor that reduced the actomyosin ATPase activity several-fold had no effect on the in vitro sliding velocity (84). Accordingly, our findings are consistent with the detachment limitation (29, 60, 61, 85) rather than attachment limitation (86) of shortening velocity in muscle fibers under physiological conditions as well as of the actin filament sliding velocity driven by a large number of myosin motors in the in vitro motility assay. On the other hand, as mentioned above, the rate of isometric force development and V_max_ of the actomyosin ATPase are primarily rate-limited by the attachment rate, k_on_(x), under physiological conditions. Additionally, increased attachment rate will increase the sliding velocity when the number of available myosin motors is considerably fewer (87) than that operating together in the muscle cell.

To conclude, this study has unveiled otherwise hidden secrets in the actomyosin cross-bridge cycle from attachment of myosin to actin over Pi release to force generation. Additionally, the model accounts for a wide range of contractile phenomena with the potential to clarify the currently quite bewildering picture of the analyzed phase of the cycle. We expect the model to be valuable in drug discovery efforts aiming to fine-tune specific transition rates in the force-generating cycle, either for adapting the function of normal myosin to abnormal conditions (e.g., altered load, surrounding areas with dead myocardium) or for correcting myosin malfunction, e.g., due to mutations or posttranslational modifications. Whereas blebbistatin is selective for myosin II, the model is likely to be generally valid for any myosin after appropriate modification of the numerical values of different rate functions. This is suggested by evidence (19) that critical states and transitions in this model (Fig. 4 *A*) are of relevance in several myosin classes.

## Author Contributions

A.M. conceived the study, developed the model, ran model simulations, and analyzed model data in relation to experiments. M.A.R. performed most experiments on isolated proteins and analyzed the data. M.U. performed some of the experiments on isolated proteins and analyzed the data. D.E.R. performed experiments on muscle fibers and analyzed the data. A.M. coordinated the project, but all authors contributed to planning the work, to data interpretation, and to writing the article.

## Supporting Material

Document S1. Supporting Materials and Methods, Figs. S1–S11, and Tables S1–S5

Document S2. Article plus Supporting Material
