# Supplementary material for: Blebbistatin Effects Expose Hidden Secrets in the Force-Generating Cycle of Actin and Myosin
Source: Biophys J. 2018 Jul 17;115(2):386–97. doi: 10.1016/j.bpj.2018.05.037 (PMC6050972; doi:10.1016/j.bpj.2018.05.037)
Supplement: Document S1. Supporting Materials and Methods, Figs. S1–S11, and Tables S1–S5 [file mmc1.pdf]

Biophysical Journal, Volume *115*

Supplemental Information

Blebbistatin Effects Expose Hidden Secrets in the Force-Generating Cycle of Actin and Myosin

Mohammad A. Rahman, Marko Ušaj, Dilson E. Rassier, and Alf Månsson

Supplementary Theory

ATP-hydrolysis, cross-bridge attachment and phosphate release

The processes, involving the recovery stroke and hydrolysis of ATP to ADP and inorganic phosphate (Pi) are lumped together into one transition between the M*T and M**DP states (Fig. 4; asterisks related to tryptophan fluorescence) with forward and reverse transition governed by rate constant k_3 and k_{-3}, respectively. Myosin heads in the M**DP state are assumed to bind weakly and non-stereospecifically to actin forming an AM**DP state with equilibrium constant $K_w(x)$:

$$K_w(x)=\exp(\Delta G_w-(ksw/2)(x-x_w)^2/k_BT) \quad \text{if } x\in[-1.3,16.7]\text{nm} \qquad \text{(S1a)}$$

$$K_w(x)=0 \qquad \text{for other x-values} \qquad \text{(S1b)}$$

where, ksw is the stiffness of myosin cross-bridges in the AM**DP state and x_w is the value of x where the free energy of binding (ΔG_w) of the AM**DP state attains its minimum (Table S1). Here, ΔG_w is in units of k_BT where k_B is the Bolztmann constant and T is the absolute temperature. The invididual rates are assumed to be infinitely high. Strictly, the weakly bound state, AM**DP, is not required in the model if ksw is very low as suggested by the lack of evidence for friction forces.

The transition from the weakly and non-stereospecifically bound AM**DP state to a weakly but stereospecifically attached AM*´DP state is governed by the rate function:

$$k_{on}(x) = k_{on}{'} \exp(\Delta G_{on} - ks\,(x-x_w)^2/\,k_BT + ksw(x-x_w)^2/\,k_BT) \qquad \text{(S2)}$$

where ΔG_{on} is the difference between the free energy minima of the states AM**DP and AM*´DP (see further, Table S1).

The reversal of this process is governed by:

$$k_{on\text{-}rev}(x) = k_{on}{'} \exp(ks\,(x-x_w)^2/k_BT\; ksw(x-x_w)^2/k_BT) \qquad \text{(S3)}$$

Next, a structural change is assumed to occur to allow the myosin head to enter the AM*DP state from which phosphate is released (1). Following, Llinas et al. (1), this transition is assumed to be associated with slightly increased affinity between actin and myosin and a small swing of the myosin head or lever arm as reflected in the vertical and horizontal shifts, respectively of the corresponding free energy diagrams (main Fig. 4B). After transition into the AM*DP state, phosphate is assumed to be rapidly and reversibly released from the active site. By now sharing the strain-dependence (cf. (2)) between the forward and backward rate functions and further assuming that phosphate release is a rapid equilibrium, the following rate functions emerge:

$$k_{P+}(x)= k_{P+}{'}\exp(\Delta G_{AM*DP\text{-}AM**DP}/2\;\; -(ks/2)(x-x_1)^2/\,(2k_BT) +(ks/2)(x-x_w)^2/(2k_BT))) \qquad \text{(S4)}$$

$$k_{P-}(x)=k_{P+}{'}[Pi]/([Pi]+Kp)\, \exp\,(\Delta G_{AM*DP\text{-}AM**DP}\,/2+(ks/2)(x-x_1)^2/(2k_BT)-(ks/2)(x-x_w)^2/(2k_BT)) \qquad \text{(S5)}$$

From $-\ln(k_{P+}(x)/k_{P-}(x))$, the free energy difference between the states AM*´DP and AM*D_L is given by:

$\Delta G_{AM*'DP\text{-}AM**DL} = \Delta G_{AM*'DP\text{-}AM*DP} - k_BT\ln([Pi]/Kp) + (ks/2)((x-x_w)^2 - (x-x_1)^2)/k_BT)$ (S6)

Main force-generating transition

The main force-generating transition i.e. the power-stroke is, assumed to be a rapid equilibrium. The start of the power-stroke state (AM*D_L) has an open actin binding cleft and the lever arm in the pre-stroke position and the post-power-stroke state (AM´D_H) has the actin binding cleft closed and the lever-arm in the post-power-stroke position. The equilibrium constant is given by:

$K_{LH}(x)=k_{LH+}(x)/k_{LH-}(x)$ (S7)

where

$k_{LH+}(x)=k_{LH-}(x)\exp(\Delta G_{AM*D\text{-}AM'D} +(ks/2)(x-x_1)^2/(k_BT)-(ks/2)(x-x_2)^2/(k_BT))$ (S8)

and

$k_{LH-}(x)=2000\ s^{-1}$ (S9)

If Keq(i)>1000 according to Eq. S5, it was, for practical reasons, set equal to 1000 in Monte-Carlo simulations.

Cross-bridge detachment at the end of the power-stroke

The cross-bridge detachment occurs in several steps (3-5). First, there is a transition (6, 7) between the AM´D_H and an AMD state that opens the nucleotide pocket for MgADP release (5, 7):

$$k_5(x) = k_5(x_1)\exp(\delta G) \cdot \exp\left(\frac{k_s \cdot |x| \cdot \delta x}{k_B T}\right) \quad (S10)$$

where $\delta x=x_2-x_3$ (Table S1) corresponds to the difference in position for the free energy minima of the AM'D_H and AMD states. Further, $\delta G=\Delta G_{AM'D\text{-}AMD}+(k_s/2)(x_2^2-x_3^2)/k_BT$, i.e. the difference in free energy between the AM´D_H and the AMD states states at x=0 nm.

The next steps involve MgADP release and MgATP binding and the AMD, AM and AMT states are lumped together, giving the following rate function ($k_{off}(x)$; (8)) for the transition from the AMD to the MT state (assuming [MgADP] = 0 mM):

$$k'_{off}(x) = \frac{k_2(x)k_6[MgATP]}{\frac{k_6}{K_1}+(k_2(x)+k_6)[MgATP]} = \frac{k_2(x)[MgATP]}{\frac{1}{K_1}+\frac{k_2(x)}{k_6}[MgATP]+[MgATP]} \quad (S11)$$

where

$$k_2(x) = k_2(0)\exp\left(\frac{k_s \cdot |x| \cdot x_{crit}}{k_B T}\right) \quad (S12)$$

Here, $k_2(0)$ and k_6 govern ATP induced detachment from the AMT state at x=0 and strain-independent ADP-release from the AMD state, respectively. The constant K_1 is the equilibrium constant for MgATP binding to the AM state (Fig. 3A) and x_{crit} is a strain parameter (9) that defines strain-dependence of the MgATP induced detachment. An overall detachment rate function, from the AM´D_L state to the M**T state, is given by:

$k_{off}(x) = \frac{k'_{off}(x) k_5(x)}{k'_{off}(x) + k_5(x)}$ (S13)

The latter rate function, together with Eqs. S11-S12 can be used for simplifying the current model, allowing detachment into the M**T state directly from the AM´D_L state (Fig. S8).

There has long been evidence that the myosin-actin interaction and its relation to ATP turnover differ appreciably between elongation and shortening of active muscle (10-13). The findings imply that a large fraction of the attachment-detachment cycles during elongation are very rapid and occur without consumption of ATP. This picture is captured by the present model: First, during stretch, a majority of the cross-bridges are forcibly detached from states (particularly the AM*D_L state) prior to the main force-generating transition without turnover of ATP (Eqs. S3 and S5). Second, cross-bridges detached from the AM*D_L state are assumed to instantaneously reattach into this state at a neighboring actin site 5.5 nm toward the pointed end of the actin filament (cf. similar model for shortening in (14)). The overall detachment-reattachment process is governed by the rate function:

$$k_{\text{off-str}}(x) = k_{off-str}(x_{11}) \cdot \exp\left(\frac{k_s \cdot |(x - x_{11}| \cdot \delta x_{str}}{k_B T}\right) \quad \text{(S14)}$$

where $\delta x_{str} = 2.7$ nm. Whereas the present model rather well approximates the early phase of the force response to stretch there are limitations in the fitting of later phases. This is attributed to the presence of elastic elements in parallel with the cross-bridges (15, 16) e.g. titin, and sarcomere non-uniformities (16-19) affecting the tension response in muscle cells. It may also reflect uncertainties on how the exact values of certain kinetic constants affect the stretch response. For instance, we have tentatively used parameter values for $k_{off-str}(x_{11})$ and δx_{str} identical to those for forcible detachment of rigor cross-bridges (20).

Contractile activation, filament compliance and target zones on actin filaments

Just as in the recent model (3) we assume maximum Ca^{2+} activation and, unless otherwise stated, that a single site on the actin filament is within reach of a given myosin cross-bridge. Furthermore, we assume that only one of the globular myosin units (heads) in a pair binds simultaneously to a given target zone and that neighboring zones are separated by 36 nm. Finally the actin filaments and the myosin attachment points are assumed infinitely stiff.

Comments on parameter values, relation to experimental data and model limitations

The numerical values of the cross-bridge stiffnesses for strongly and weakly bound cross-bridge states has been motivated previously as well as the location of the free-energy minima of a majority of the states (3). The free-energy minimum of the new pre-power-stroke state (AM*´DP) as well as the weakly bound AM**DP state was here assumed to occur at 8.7 nm, consistent with a small structural change (1) upon transition into the Pi-release state (AM*DP) with free energy minimum at x=7.7 nm.

The present model fits a range of data in the absence of blebbistatin. Thus, the maximum sliding velocity for long filaments is 9.4 µm/s similar to our experimental values of 8-11 µm/s in the temperature range from 28-31 ^{o}C. During steady-state isometric contraction the model predicts an average force per cross-bridge of 6 pN, similar to values from experiments on single molecules from fast mammalian muscle (21). Additionally, the predicted force enhancement during stretch, relative the isometric force, is similar to experiments (cf. this study). Also the Vmax and K_{ATPase} values (84 s^{-1} and 0.4 mM) of the actomyosin ATPase are

similar to appropriately temperature corrected (3) experimental data at close to physiological ionic strength used here (cf. (22)). The K_M^v value for the velocity vs [MgATP] is about 5-fold lower than in experiments. Whereas this could be amended by making the cross-bridge stiffness non-linear with low stiffness for cross-bridge strains that counteract sliding (3, 8) we did not introduce this complication because the details are poorly understood (cf. (8)). Finally, the relationship between [Pi] on the one hand and tension and velocity on the other hand are consistent with results in the litterature (Fig. S1).

Possible effects of uncertainties in parameter values

In our simulations we use parameters fixed at literature values only changing the parameter value of relevance for testing a particular hypothesis on basis of blebbistatin effects on the actomyosin ATP turnover rate. If the simulation results strongly depend on the exact parameter values that are used, erroneous conclusions may emerge as a result of uncertainties in these values. It is therefore desirable with an error propagation analysis where errors in each parameter value propagate through the analysis to investigate how these errors affect the conclusions. Unfortunately this approach suffers from two problems. First it is, for several reasons, difficult to obtain reliable and/or consistent error estimates of different parameters values from the litterature. For this reason, we assigned a reasonable lower and higher bound of the parameter value corresponding to 75 % and 125 % respectively of the mean value used. The second problem that prevents a full error propagation analysis is of combinatorial origin. Thus, if all combinations of lowest (mean value – error) and highest (mean + error) parameter value would be tested for each of the 25 parameters in Tables S1-S2, this would lead to 2^{25} 33 000 000 simulation runs. This is clearly incompletely unrealistic considering that some of the individual Monte-Carlo simulations (e.g. for length velocity plots) take several hours.
Nevertheless, in an effort to test the robustness of our conclusions by an approach related to a full investigation of the type considered above, we performed a limited error propagation analysis based on random sampling. First, we assumed (see above) that each parameter value is either 25 % higher or 25 % lower than the mean value currently used and then we andomly assigned either the lower value or the higher value to each parameter. If this assignment resulted in a priori unreasonable value of any parameter, e.g. giving unreasonable free energy profiles, the closest reasonable parameter value was used. “Unreasonable“ free energy profiles are either inconsistent with the free energy of ATP-turnover or the x-values for minima of the free energy are increased rather than decreased for sequential states from attachment towards detachment. Following this corrective step, the selected combinations of low and high parameter values were used to simulate the physiological force-velocity relationship because such simulations and the associated analysis could be conducted in minutes. Next, the sets of selected parameter values that gave reasonable shapes of the force-velocity relationship and three other set where used as basis for testing the two major models (change of kp+(x) or change of k_{on}(x).

Simulations of contraction and actomyosin interactions at 5 ^{o}C

In our treatment we focused on the parameters $k_{3,}$ k_{on}(x), k_2 and ΔG_{LH} for which there is evidence for significant temperature effects. These parameter values were changed to those given in Table S3 for the simulation of isometric tension and the tension response to active stretch at 5 ^{o}C. Other parameter values were left at those in Tables S1-S2. The rate constants k_3 and k_{on}(x) were assumed to have Q_{10} values of ~4 in agreement with high temperature sensitivity of these processes found previously (23-27). The free energy difference ΔG_{LH} (see below) was reduced from 14 k_BT at 25-30°C to 6 k_BT at 5 ^{o}C in order to account for the >50 % reduction of the average cross-bridge strain during isometric contraction of rabbit psoas fibers under these conditions (27, 28). The temperature sensitivity of the cross-bridge

detachment rate after the end of the power-stroke is primarily conferred by the rate constant k_2 with Q_{10} ~2 (29). In our simulations we tentatively assumed Q_{10} =2 for kp+(x).

The above changes in parameter values gave an approximate Q_{10} value of 3-4 for the maximum actin-activated ATP turnover rate (in the range 5 to 25-30 ^{o}C) somewhat lower than the experimental value of 5 (22). In addition, the change in parameter values predicted 2-3x increase of the maximum isometric force for an increase in temperature from 5 to 30 ^{o}C (Q_{10} ~1.3-1.6) similar to experimentally observed effects (3-4x increase; Q_{10} ~1.5-1.9;(27, 30, 31)). The predicted maximum velocity of shortening was increased 9-fold (Q_{10} ~2.4) quite similar to experimental data with Q_{10} ~2.0 (32). The maximum force during stretch was little affected (increase by 8 %) by the change in parameter values corresponding to increase in temperature from 5 to 25-30 ^{o}C. This small effect of temperature on the force during stretch is consistent with experimental results (30).

Supplementary Results

Ionic strength effects

The lower effect of blebbistatin on the sliding velocity at reduced ionic strength may have different grounds. The possibility that the affinity of blebbistatin to myosin is reduced at lower ionic strength is contradicted by similar effect of ionic strength on the fractional inhibition at 1 and 30 µM blebbistatin. The remaining possibilities include 1. different local effects of blebbistatin on myosin structure at low and high ionic strength or 2. modulated blebbistatin effects by different ionic strengths due to different steady-state distributions between different actomyosin states under these conditions. However, Monte-Carlo simulations suggest that the blebbistatin effect on velocity by the favored mechanism in the main paper is negligibly attenuated by 12-fold increased affinity (23) for the weak-binding state (AM**DP) corresponding to a reduction in ionic strength from 130 mM to 60 mM. However, appreciably increased actin affinity in the Pi-release state (AM*DPi) upon lowered ionic strength would enhance the attenuating effect of this intervention so that velocity reduction in response to 1 µM blebbistatin would be 35-40 % at 60 mM ionic strength compared to 45 % at 130 mM strength. This accords with the idea (1) that the actin-binding of myosin in the Pi-release state (AM*DP state in the present model) is to a large extent mediated by ionic interactions. However, it is premature currently to consider this issue in detail due to lack of quantitative information about possible changes in actin affinity in the AM*DP state.

Possible effects of uncertainties in parameter values

Random change of all parameter values in Table S1 and S2 either up or down by 25 % (values used shown in Table S5) caused, in a majority of the cases (7/9), the force-velocity relationship to deviate appreciably from the experimental force velocity data (Fig. S9). In some cases (2/9) the force-velocity relationship was quite similar to that observed experimentally. For these cases as well as one case were the parameter values were changed away from the random selections to give better fit to the force-velocity data we performed a full set of simulations of the blebbistatin effects assuming that blebbistatin either reduced k_{p+}(x) or k_{on}(x). Similar simulations were also performed for three randomly selected sets of parameter values giving poor fits to the force-velocity data The results of these simulations are illustrated in Fig. S11 showing a comparison between the sum of squared deviations for all parameter values in Table S4 for the two different models. It is clear from Fig. S11 that, for all sets of parameter values tested the sum of the squared deviations between model and experiments were smallest for the model assuming that blebbistatin reduces k_{p+}(x).

Supplementary Methods

Chemicals and Materials

Blebbistatin [(+/-)-1-Phenyl-1,2,3,4-tetrahydro-4-hydroxypyrrolo[2,3-b]-7-methylquinolin-4-one] was purchased from Toronto Research Chemical (cat. no.TRC-B592490-10) for the in vitro motility assays. Other chemicals were of analytical grade and purchased from Sigma Aldrich except Rhodamine Phalloidin that was from Thermo Fisher Scientific (cat. no. R415).

Protein preparations

Actin, myosin and HMM were prepared from fast skeletal muscle of New Zealand white rabbits (33, 34). To obtain myosin with phosphorylated RLCs (resulting in partial phosphorylation; pP-myosin),minced muscle was extracted for 20 min in 60 ml/20g ice cold Guba Straub solution (0.3 M KCl, 0.1 M KH_2PO_4, 0.05 M K_2HPO_4; pH 6.5). The extract was then centrifuged at 11000 g for 30 min at 4^0C. The supernatant was filtered through two layers of gauze followed by addition of 14 volumes of ice cold buffer A (5 mM potassium phosphate buffer, pH 7.0; with 0.1 mM DTT). After 2 hours incubation, the precipitated myosin filaments were collected by centrifugation at 11000 g for 18 min at 4^oC. The pellet was re-suspended in 2-3 ml of buffer B (20 mM potassium phosphate buffer, pH 8.0; with 0.5 M KCl, 5mM Na_2ATP, 12.5 mM $MgCl_2$x6H_2O and 0.1 mM $CaCl_2$x2H_2O). The above procedure was repeated in obtaining dephosphorylated myosin (dP-myosin) but buffer A was exchanged for buffer C (1 mM EDTA and 0.1 mM DTT) and buffer B for buffer D (20 mM MOPS, 1 mM DTT and 0.5 M KCl; pH 7.0). In preparation of pP-myosin, the solution (pellet re-suspended in buffer B or D) was next kept for 30 minutes at room temperature followed by ultracentrifugation at 120 000 g for 2 hours and 30 minutes at 4^oC. The supernatant was then precipitated with 14 volumes of degassed ice-cold distilled water for 30 minutes at 4^oC followed by an additional centrifugation at 6500 g for 10 minutes at 4^oC. In the preparation of pP-Myosin, the precipitate in the final step was re-suspended in buffer B and ultracentrifugation was repeated with collection of the supernatant that was then used immediately for HMM preparation. In the preparation of dP-myosin, 1 ml of buffer D was added to the pellet followed by storage on ice overnight, to ensure that myosin is fully dephosphorylated. On the next day, the pellet was re-suspended in 1-2 ml of buffer D and ultracentrifugation was repeated followed by HMM preparation.

Gel electrophoresis

The purity and integrity of actin, myosin and heavy meromyosin were confirmed by SDS-PAGE (ThermoFisher Scientific; cat. no. NP0342BOX). The level of myosin RLC phosphorylation was analyzed by 8 M Urea PAGE slightly modified from previous work (35) by using 25 mM Tris-Glycine running buffer containing 6 M Urea.

In vitro motility assays

In vitro motility assays (IVMA) were performed (8) by adsorbing HMM to coverslips silanized with trimethylchlorosilane (TMCS). Assay solutions were prepared in buffer E (10 mM MOPS, 1 mM $MgCl_2$, 0.1 mM K_2EGTA; pH 7.4). Buffer F (10 mM MOPS, 50 mM KCl, 1 mM DTT, 1 mM $MgCl_2$, 0.1 mM K_2EGTA) was used to rinse the flow cells and for diluting HMM and F-Actin. In an in vitro motility assay, the flow cell was first incubated with HMM (30 µg/ml or 120 µg/ml) for 2-5 minutes followed by incubation and washing steps as follows: 1mg/ml BSA (2 min), buffer F (30 s), 1µM blocking actin (non-fluorescent actin filaments in buffer F; 1-2 min), 1mM MgATP in buffer F (30 s), 2 x buffer E (30 s each), 2-10 nM rhodamine-phalloidin labeled actin filaments, buffer E (30 s) prior to initation of the assay. Assay solution (10 mM DTT, 45-135 mM KCl, 3 mg/ml Glucose, 0.1 mg/ml glucose oxidase, 0.02 mg/ml catalase, 2.5 mM creatine phosphate, 0.2 mg/ml creatine phosphokinase,

0.01-1 mM MgATP) was prepared with methyl cellulose (0.64%) in buffer E if the ionic strength was ≥ 80 mM. In some experiments, the incubation step with blocking actin was not included, e.g. in cases when velocity was studied vs. filament length Blebbistatin (mixed enantiomer) was dissolved in N,N-Dimethylformamide (DMF; Sigma Aldrich; cat. no. 227056) to a final concentration of 16.27 mM whereas S-(-) blebbistatin was dissolved in dimethyl sulfoxide to a final concentration of 17.91 mM. Both were aliquoted and stored in the dark (-20^{0}C). As suggested by control experiments at the final concentrations used, DMF per se, did not affect the HMM induced actin filament sliding in the in vitro motility assay. The blebbistatin concentration was based on absorbance spectrophotometric analys using an exctinction coefficient of 7400 M^{-1} cm^{-1} at a wavelength of 422 nm. The blebbistatin aliquot was diluted in buffer E, 1 hour prior to the in vitro motility assays. The flow cells were incubated with blebbistatin for 15 minutes (36) before adding the assay solution with the same blebbistatin concentration. Prolonged illumination with the wavelength used for visualizing the rhodamine phalloidin labelled actin filaments did not affect the results.
Actin filament movements were recorded using an electron multiplying charge coupled device (EMCCD) camera (C9100-12, Hamamatsu Photonics) with a frame rate in the range 4-10 f/s. In the data analysis, actin filament sliding velocities were calculated as described (Månsson & Tågerud, 2003) earlier. At low velocities (either due to a high blebbistatin concentration or a low [MgATP]) every 3rd or 4th frames were considered until in total 15 frames were analyzed. The cut-off of the coefficient of variation (CV) (standard deviation of frame-to-frame velocity divided by average velocity in ten frames) (Månsson & Tågerud, 2003) for inclusion of data in velocity analysis varied between 0.2 and 0.4 with negligible effects on the results. For analysis of velocity vs length plots, all data was used independent of the CV value and the average velocity was calculated over 2.6-10.4 s, with the shortest time for the highest velocities (~10 µm/s) and the longest time for the lowest velocities (< 2 µm/s). This was important because simulations indicated that some models for the blebbistatin effect would reduce average velocity by producing pauses in filament sliding. Such effects would not be captured by analysis of the experimental data if CV based cut-offs had been used.

Muscle fiber experiments
Muscle bundles of rabbit psoas were dissected and permeabilized following standard procedures (37). Muscles were incubated in rigor solution (pH = 7.0) for ∼4 h, after which they were transferred to a rigor-glycerol (50:50) solution for ~15 h. The samples were subsequently placed in a fresh rigor-glycerol (50:50) solution with the addition of a cocktail of protease inhibitors (Roche Diagnostics) and stored in a freezer (−20°C) for at least 7 days. On the day of the experiment, a small section of the sample was cut (∼4 mm in length), and single fibers were dissected in relaxing solution (see below). The fibers were gripped at their ends with T-shaped clips made of aluminum foil and were transferred to a temperature-controlled chamber to be attached between a force transducer (resonant frequency 1 kHz) (model 403A, Aurora Scientific, Toronto, ON, Canada) and a length controller (model 312B, Aurora Scientific). The rigor solution (pH 7.0) was composed of (mM) 50 Tris, 100 NaCl, 2 KCl, 2 $MgCl_2$, and 10 EGTA. The relaxing solution used for muscle storage and dissection (pH 7.0) was composed of 100 KCl, 2 EGTA, 20 imidazole, 4 ATP, and 7 $MgCl_2$. The experimental solutions with pCa^{2+} of 4.5, 5.0, 5.5, and 6.0 (pH 7.0) contained 20 imidazole, 14.5 creatine phosphate, 7 EGTA, 4 MgATP, 1 free $Mg^{2+,}$ free Ca^{2+} ranging from 1 nM (pCa^{2+} 9.0) to 32 µM (pCa^{2+} 4.5), and KCl to adjust the ionic strength to 180 mM. A pre-activating solution: 68 KCl, 0.5 EGTA, 20 imidazole, 14.5 creatine phosphate, 4.83 ATP, 0.00137 $CaCl_2$, 5.41 $MgCl_2$ and 6.5 HDTA; pH 7.0, pCa^{2+} 9.0) with a reduced Ca^{2+} buffering capacity was used immediately before activation. Blebbistatin was dissolved in dimethylformamide (DMF) and was stored at −20°C before use. On the day of the

experiment, blebbistatin was diluted in 4 ml of activating (pCa^{2+} 4.5) or relaxing (pCa^{2+} 9.0) solution to reach final concentrations of 1, 2, 5 or 10μM. A red filter (650 nm) was placed on the light source of the microscope to avoid exposure of blebbistatin to light during the experiments, as it loses its effectiveness in wavelengths between 365 and 490 nm (38).

Fibers were activated in the presence or absence of blebbistatin (n = 16). All experiments were performed at 5°C. The initial SL was adjusted to ~2.5 μm (optimal length, *L*o) before fiber activation. The fibers were first activated at a pCa^{2+} of 4.5 and stretched by 5 or 10% *L*o, at difference velocities from 0.4-2 *L*o·SL·s−1. After that, the fibers were incubated in relaxing solution (pCa^{2+} = 9.0) containing blebbistatin. After blebbistatin incubation in relaxing solution (15 min), the fiber was immersed in activating solution also containing blebbistatin. After full force development, similar stretches as in the absence of blebbistatin were applied to the fibers. Control contractions at a pCa^{2+} of 4.5 were elicited through the experiments; at the end of the experiments the isometric forces never decreased by >10% (actual range: 5.2–8.3%) from the maximal force produced at the beginning of the experiment (Po). When the striation pattern of the muscle fibers became unclear such that it did not allow measurements of SL, the experiments were ended.

The transition between the two phases of force rise during a stretch was detected with a two-segment piecewise regression (37).When the piecewise regression did not detect the transition point based on these criteria, we extrapolated the two lines visually to detect te breakpoint. Visual inspection provided results similar to regression analyses when both methods could be compared. The intersection between the two slopes representing the fast and slow increases in force was used to define Pc, calculated as the relative increase in force obtained from the maximal isometric force developed before stretch (Po) in any given condition.

Data analysis for muscle fiber experiments. Force was measured just before stretch (isometric force, F_{iso}) and at a breakpoint between two phases of force increase during stretch defining the critical force (F_c). The transition between the two phases was evaluated by differentiation of force during the stretch phase [d(force)/d*t*)](39).

Statistical analysis

Data are presented as mean ± 95 % confidence limits unless otherwise stated. N represents number of actin filaments for in vitro motilitya assays and the number of muscle fibers tested for muscle fiber experiments. All statistical analyzes and curve fittings in relation to experiments on isolated proteins and modelling were performed using the GraphPad Prism software (Version 6.07; GraphPad software Inc, USA).

Monte Carlo simulations

For the purpose of our simulations we made the simplifying assumption that HMM motor fragments are adsorbed to motility assay surfaces with uniform density, ρ (5000 μm^{-2}) and that myosin heads in a band of d=30 nm width around the long axis of the filament are available for binding (40). Then it was assumed that all 36 nm intervals along an actin filament are identical. That is we assumed that the total number of available myosin heads (n=ρdl) along the entire filament of length, l, is distributed uniformly between 360 bins each of 0.1 nm width (3). The simulations started with all myosin heads in the M**DP and M**DP-bleb states at the appropriate equilibrium ratio assuming a blebbistatin affinity of 1 μM^{-1} (41). The time, Δt, until the first/next update event was calculated using the Gillespie algorithm (42) from the inverse rate summed over all possible chemical transitions at each discret value, $x=x_{bin}$, as described previously (3). The simplifying assumption was made that

blebbistatin binds to myosin in the MDP or MT states with a dissociation constant K_B, and then undergoes obligatory dissociation from myosin in the AM´D_H state. This is assumption is essential in order not to overwhelm the computational power. In order to maintain a roughly constant blebbistatin-bound fraction of myosin, despite the assumed obligatory dissociation from the AM´D_H state, we assumed that the rates associated with the blebbistatin binding-equilibrium are orders of magnitude faster than found in experiments. The consequences of these simplifying assumptions were minimal as suggested by simulations where the rate was changed 100-fold.

Numerical solution of differential equations

For simulation of actomyosin ATPase in solution and estimates of V_{max} and K_M, for this relationship we used Simnon (version 1.3; SSPA, Gothenburg, Sweden) to numerically solve the system of differential equations for the kinetic scheme in main Fig. 4A. The rate constants were those at the minima of the free energy diagrams in Fig. 4B.

Table S1. Parameter values[a] determining shape of free energy diagrams for simulation of contractile properties of fast mammalian muscle at 25-30 ^{o}C

**Parameter**	**Numeri-cal value**	**Range from litterature**	**Value used in testing hypothesis for blebbistatin effect**[b]	**References and comments**
x_w (AM**DP)	8.7 nm	Set equal to x_1	-	(1) Details in (3)
x_1 (AM*´DP)	8.7 nm	~1 nm higher than x_{11}	-	Based on small structural change between prepowerstroke state and Pi-release state
x_{11} (AM*D_L)	7.7 nm	~8 nm	-	(43)[b] Details in (3)
x_2 (AM*D_H)	1.0 nm	0.9-1.1 nm	-	(6)[c] Details in (3)
x_3	0 nm		-	By definition
ΔG_w (M**DP-AM**DP)	0 k_BT; corresponds to K_w=1	~0 k_BT	-	(44) Details in (3)
$\Delta G_{AM**DP-AM*´DP} \equiv \Delta \mathbf{G_{on}}$	0.7 k_BT	Due to surface loops	Model I: (Table S4): -4k_BT (reduces $k_{on}(x)$; Eq. S2)	
$\Delta G_{AM*´DP-AM*DP} \equiv \Delta \mathbf{G_P}$ (AM*´DP – AM*DP)	1 k_BT	Due to surface loops	-	(22, 45) and Q_{10} = 2.7-3.7 in range 20-35°C. Details in (3)
$\Delta G_{AM*DL-AM´DH} \equiv \Delta \mathbf{G_{LH}}$ (AM*D_L-AM*D_H)	14 k_BT	$\Delta G_{AM*DL-AM'DH}$ + $\Delta G_{AM´DH-AMD}$ (10-20 k_BT)	Model III: 2.5 k_BT	(43, 46) Details in (3)
$\Delta G_{AM*DH-AMD}$ (AM*D_H AMD)	2 k_BT	See previous row; 1-2 k_BT	-	(8, 43) Details in (3)
ΔG_{ATP}	13.1 + ln ([MgATP] / ([MgADP] [Pi]) k_BT	Free energy of ATP-hydrolysis	-	(47)
ks	2.8 pN/nm	2.5-2.8 pN/nm	-	(43) [b]
ksw	0.02 pN/nm	-	-	Details in (3)

Footnotes to Table S1
NA: Not applicable IS: ionic strength
[a] The parameter values were from two-headed myosin motor fragments from fast skeletal muscle of rabbit at 25-30°C, ionic strength 130-200 mM, pH 7-8 unless otherwise stated.
[b] "-" means that control value was always used

Table S2. Parameter values[a] defining rate functions and kinetic constants for simulation of contractile properties of fast mammalian muscle at 25-30 ^oC

Parameter	Numerical value used	Litterature value(s)	Value used in testing hypothesis for blebbistatin effect	References and comments
$k_{+3} + k_{-3}$ (Recovery stroke+hydrolysis)	220 s^{-1}	200-500 s^{-1} Assuming Q_{10} in range 3-4 (23)	-	(23, 25) and references therein
K_3	10	2-10	-	(23, 25) and references therein. Details in (3)
$k_{on}´$	130 s^{-1}		-	(25, 45). Fine-tuned to fit rate of rise of force, V_{max} of actomyosin ATPase and phosphate transient
$k_{P+}´$	1000 s^{-1}		Model II: 1.5 s^{-1}	(20) High to ensure high maximum velocity (see text)
$k_{off\text{-}str}(x_{11})$	0.016 s^{-1}		-	(20) Forcible detachment of cross-bridges during stretch (see further text)
δx_{str}	2.7 nm		-	(20) Forcible detachment of cross-bridges during stretch
k_{-5}	2000 s^{-1}		-	Details in (3)
Kc	10 mM	~1-10 mM (temp corrected)	Model IV: 0.1-1000 (not analyzed in detail)	(45) Details in (3)
x_{crit}	0.6 nm	< 0.2 nm (see further, Theory)	-	(21) Details in (3)
k_6	5000 s^{-1}	>3500 s^{-1}	-	(29) Details in (3)
k_{-6}	14 290 mM^{-1} s^{-1}	>10 000 mM^{-1} s^{-1}	-	(29) Details in (3)
Physio-logical [Pi]	0.5 mM	~ 0.5 mM		(48)
K_1	1.7 mM^{-1}	1.7 mM^{-1}	-	(29) Details in (3)
k_2	2000 s^{-1}	1400 s^{-1}	-	(29) Details in (3). Higher value used to account for high maximum shortening velocity

Footnotes to Table S2

[a] The parameter values were from two-headed myosin motor fragments from fast skeletal muscle of rabbit at 25-30°C, ionic strength 130-200 mM, pH 7-8 unless otherwise stated.

[b]Fit of force velocity relationship. See text.

[c]From the assumption of a diffusion limited rate constant > 10 000 mM^{-1} s^{-1} (14 000 mM^{-1} s^{-1})

Table S3. Parameter values[a] for simulation of contractile properties of fast mammalian muscle at 5 °C

Parameter	Numerical value used for control simulations	Value used in testing hypothesis for blebbistatin effect	References and comments
Δ **G_{on}**	0.7 k_BT	Model I: -4 k_BT	
$\Delta\, G_{AM*DL\text{-}AM'DH} \equiv$ Δ **G_{LH}** ($AM*D_L$-$AM*D_H$)	6 k_BT	-	(28) Temperature induced decrease in average cross-bridge strain during isometric contraction in (28) requires decrease in the parameter value for effect to be accomodated by the present model
$k_{+3} + k_{-3}$ (Recovery stroke+hydrolysis)	12.5 s^{-1}	-	(24, 25) and references therein
K_3	4	-	(24, 25) and references therein
k_{on}'	25 s^{-1}	-	(24, 25) and references therein
k_2	413 s^{-1}	-	(29)
k_{P+}'	250 s^{-1}	Model II: 0.4 s^{-1}	

[a]Parameter values not given here are assumed identical to those given in Tables S1-S2

Table S4. Effects of critical changes in model parameters according to three key models, compared to experimentally observed blebbistatin effects[a]

**Variable**[b] (B: blebbistatin)	**I. ΔG_{on} from 0.7 to -4 k_BT (reduced $k_{on}(x)$)**[c]	**II. k_{p+}' down from 1000 to 1.5 s^{-1}**[d]	**III. k_{p+}' down from 1000 to 1.5 s^{-1} and ΔG_{LH} from 14 to 2.5 k_BT**[e]
Velocity > 2 µm long filaments (1 µM B)	***0.1842*** **(+)**	8.86 10^{-5} (+)	6.13 10^{-5} (+)
Velocity (0.5-1.5 µm filament; [B] with close to half maximum vel.)	***0.1062 (+)***	0.0005327 (+)	0.0003556 (+)
Velocity > 2 µm long filaments(10-30 µM B)	***0.1951*** **(+)**	0.04171 (+)	0.04227 (+)
Velocity (0.5-1 µm filament; [B] with half maximum velocity)	0.000897 (+)	0.03013 (+)	0.03548 (+)
Velocity (1 µM B; 0.01 mM MgATP)	0.08697 **(-)**	0.002567 (+)	0.001380 (+)
Velocity vs [MgATP], K_M (1 µM B)	*0.071289* (+)	0.0619 (+)	0.03378 (+)
Isometric force (2 µM B)[g]	0.004976 (+)	0.004112 (+)	0.01852 (+)
Isometric force (10 µM B)[g]	0.01233 (+)	0.003812 (+)	0.0002171 (+)
Force enhancement during stretch (2 µM B)[g]	***0.1501 (-)***	***0.09359 (-)***	0.02182 (-)
Force enhancement - stretch (10 µM B)[g]	***0.1054 (+)***	***0.01084 (+)***	9.831 10^{-5} (+)
V_{max}:Actomyosin – ATPase (saturating B)	0.000015 (+)	<1 10^{-6} (+)	<1 10^{-6} (+)
K_{ATPase} (saturating B)	***3.91*** **(-)**	***0.192811 (+)***	***0.192811 (+)***
Total SS[f]	**4.8284**	**0.4421**	**0.3468**

[a] Numerical values refer to squared difference between fractional blebbistatin/control values in model and experiments. Changes in the model are in same (+) or different (-) direction compared to experiments. Numbers in both bold and italics indicate particularly poor fits. One HMM preparation and one batch of blebbistatin used in the experiments to allow use of one value of the blebbistatin affinity (K_B=1 µM^{-1}) in the simulations. The velocity for short (0.5-1.5 µm) filaments was an exception where different blebbistatin batches were used. The blebbistatin concentration studied in this case was that which lowered the maximum velocity by 50 %.

[b] 1 mM MgATP, 130 mM ionic strength and long filaments (>5 µm) unless otherwise stated for in vitro motility assay data.

[c] Corresponds to blebbistatin-inhibition of normal rate limiting step of actomyosin ATPase.

[d] Corresponds to blebbistatin-induced inhibition of the transition from the AM*´DP state to the Pi-release state (AM*DP)

[e] Corresponds to blebbistatin-induced inhibition of the main force-generating transition.

[f] Statistical analysis using Friedman´s test for pairwise comparison of squared deviations, followed by Dunn´s post hoc tests, suggests that hypotheses 2 and 3 are better than hypothesis 1 ($p \approx 0.043$ and $p \approx 0.007$, respectively) whereas there is no significant difference between hypotheses 2 and 3.

[g] For both stretch and isometric force the simulated data that were employed in this analysis assumed high-temperature parameter values (Tables S1-S2) as only relative differences between blebbistatin data and control data are considered.

Table S5: Parameter values that were varied and used for the simulations in Fig. S9 testing effects of errors in literature parameter values[a]

	1	2	3	4	5	6	7	8	9	10[b]
K_{+3}	150	150	250	150	150	150	250	150	150	150
k_{-3}	15	15	15	15	25	25	25	15	15	25
k_{on}'	97.5	97.5	162.5	162.5	97.5	162.5	97.5	97.5	97.5	97.5*
k_{P+}'	1250	1250	750	1250	750	1250	1250	1250	1250	1250*
k_{-5}	1500	2500	1500	1500	1500	2500	2500	1500	2500	1500
Kc	12.5	7.5	12.5	12.5	7.5	7.5	12.5	7.5	12.5	7.5
x_{crit}	**0.6**	0.45	0.45	0.45	0.75	0.75	0.75	0.75	0.75	0.75
k_6	5000	5000	3500	3500	5000	3500	3500	5000	3500	5000
K_1	2.125	1.275	1.275	2.125	2.125	1.275	2.125	1.275	1.275	2.125
k_2	1500	1500	2500	1500	2500	2500	1500	1500	2500	2500
x_1	0.75	1.25	0.75	0.75	0.75	0.75	1.25	1.25	0.75	0.75
x_{11}	0	0	0	0	0	0	0	0	0	0
x_2	-8.375	-8.375	-5.025	-8.375	-8.375	-8.375	-5.025	-8.375	-5.775	-8.375
x_3	-9.625	-9.625	-5.775	-8.375	-9.625	-9.625	-5.775	-9.625	-5.775	-9.625
ΔG_{on}	0	0	2	2	0	0	0	2	2	0
ΔG_P	0	2	2	2	0	2	2	2	2	0
ΔG_{LH}	10.5	18	18	18	10.5	18	18	18	10.5	14*
$\Delta G_{AM*DH-AMD}$	2.5	2.5	1.5	1.5	2.5	1.5	1.5	2.5	2.5	2.5
ks	2.1	3.5	3.5	3.5	2.1	2.1	3.5	3.5	3.5	2.1

[a]Parameter values in red fonts gave bad fits to experimental force-velocity data (see Fig. S9)

[b]Numerical values in this column (10) not obtained by random selection. Instead, parameter values in column 10 are identical to those in column 5 except for the fact that the parameters labelled with asterisk have been modified to improve the fit of the force-velocity relation in the absence of blebbistatin. This approach was taken in order to obtain three sets of parameter values with good fit.

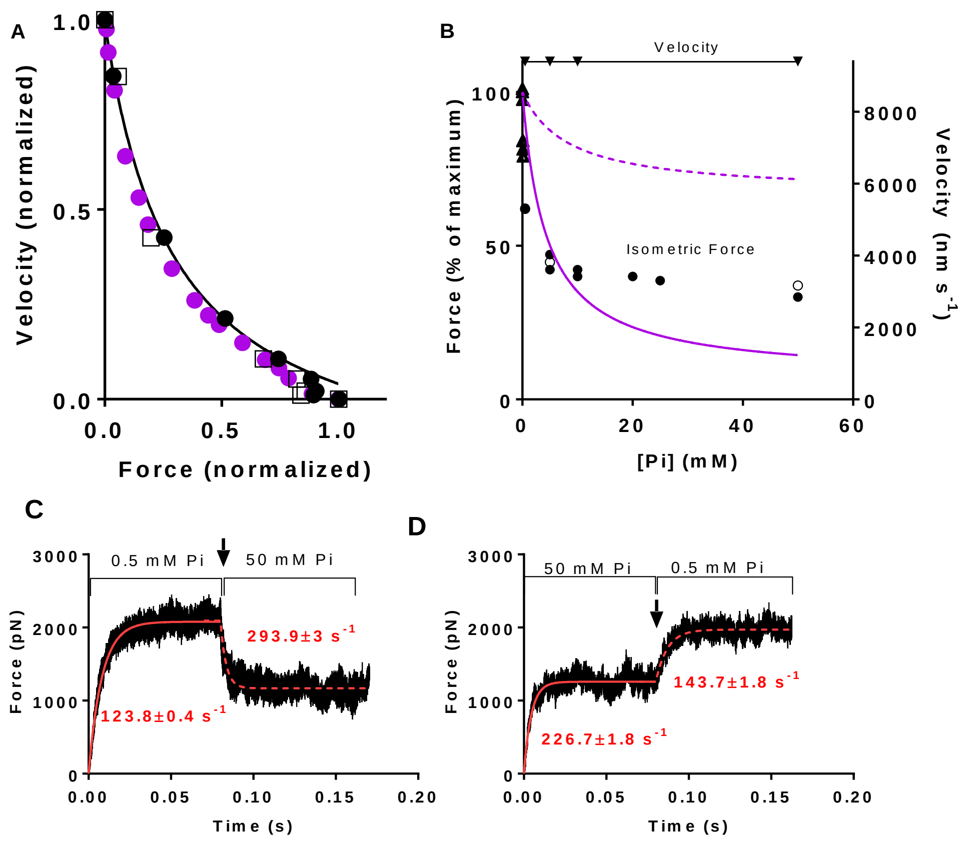

Figure S1. Comparison between model predictions and experimental data (purple symbols) A. Force-velocity data normalized to maximum isometric force and maximum velocity. Absolute velocity values given in B. Experimental data (purple symbols) as in (3) from intact mouse skeletal muscle (49) fitted by the Hill (50) hyperbolic equation (line). Model predictions: black symbols (filled symbols: Monte-Carlo simulations; open symbols: obtained by solving differential equations). Note, both simulated data and experimental data deviate from the hyperbolic relation at high force (c.f. (3, 4, 51)). **B.** Model simulations of the relationship between isometric force and velocity (triangles) on the one hand and concentration of inorganic phosphate on the other, compared to experimental force data (purple) from rabbit psoas myofibril (full line) at 15°C (52) and muscle fiber (dashed line) at 30 °C (53). The model predictis negligible changes in sliding velocity with altered [Pi] in accordance with experiments (14, 48, 54). Filled symbols: Monte-Carlo simulations; open symbols: obtained by solving differential equations. **C.** Transient tension changes in response to an instantaneous change in [Pi] from 0.5 mM to 50 mM after 0.08 s during an isometric contraction initiated at 0 s. Monte-Carlo simulations assuming a 30 µm long filament and at myosin head density on the surface of 5000 µm^2. Rate constants (± 95 % CIs) given in red text in the figures refer to single exponential fits to data by non-linear regression. **D.** Transient tension changes in response to an instantaneous change in [Pi] from 50 mM to 0.5 mM after 0.08 s during an isometric contraction initiated at 0 s. Other conditions similar to those in C. Note similar rates of Pi-transient and initial rate of rise of tension at a given Pi-concenctration. These rates are somewhat higher than the V_{max} of the actomyosin ATPase (83 s^{-1}) in solution because the latter is also influenced by the rate of the ATP hydrolysis/recovery stroke on the myosin active site.

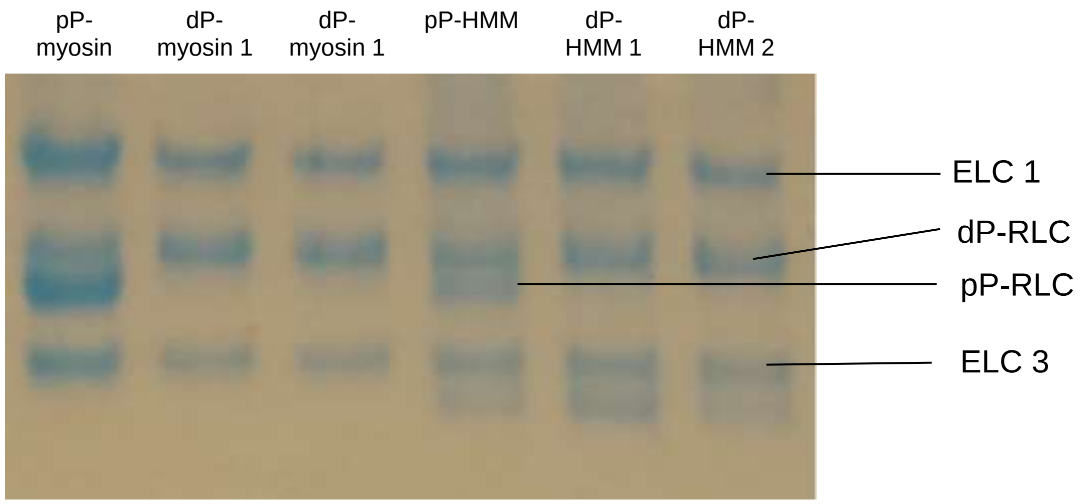

Figure S2. Polyacrylamide gel (12 %) electrophoresis of myosin and HMM in 8M urea with partly phosphorylated (pP-myosin and pP-HMM) and fully dephosphorylated (dP-myosin and dP-HMM) regulatory light chains. ELC 1 – long isoform of myosin essential light chain, dP-RLC – dephosphorylated regulatory light chain, pP-RLC – phosphorylated regulatory chain, ELC 3 – short isoform of myosin essential light chain. Band below ELC 3 with HMM due to 17 kDa fragment of RLC (55) that is co-migrating with the RLC in SDS PAGE. The myosin and HMM preparations were those (labelled 1 and 2, respectively) used for the present experiments, including all data in main Fig. 1 and Fig. S3.

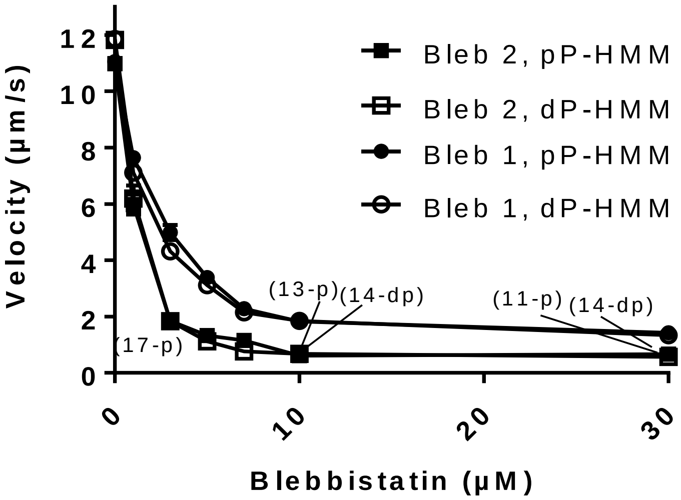

Figure S3. Concentration-response curves for effects of blebbistatin on sliding velocities in the in vitro motility assay. Data obtained using HMM with partially phosphorylated (filled symbols) or fully dephosphorylated (open symbols) regulatory light chains with two batches of blebbistatin (Bleb1 and Bleb 2). Data,given as mean ± 95 % CI, were obtained at130 mM ionic strength (1 mM MgATP). Results from > 20 filaments if not stated otherwise in parentheses (with pP or dP to indicate pP-HMM and dP-HMM, respectively) The data for bleb 2 and dP-HMM are reproduced from Fig. 1 in main paper.

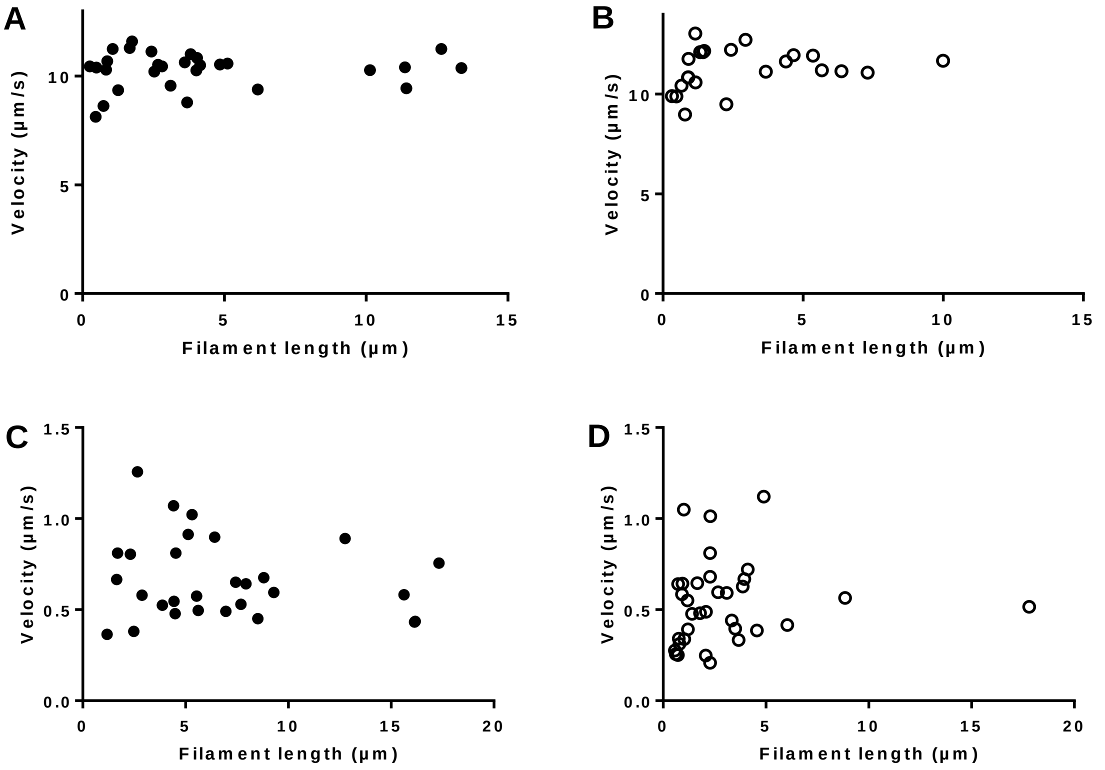

Figure S4. Velocity vs length plots using both partially phosphorylated (pP-HMM (left panels; A, C) and fully dephosphorylated (dP-HMM; right panels; B, D). A and **B,** No blebbistatin. **C** and **D,** 7 µM blebbistatin. An incubation step with blocking actin (non-fluorescent sheared actin filaments; see Methods) was used in the experiments to block dead heads from interfering with the observed fluorescence labelled filaments. The blebbistatin batch with highest activity was used for these experiments.

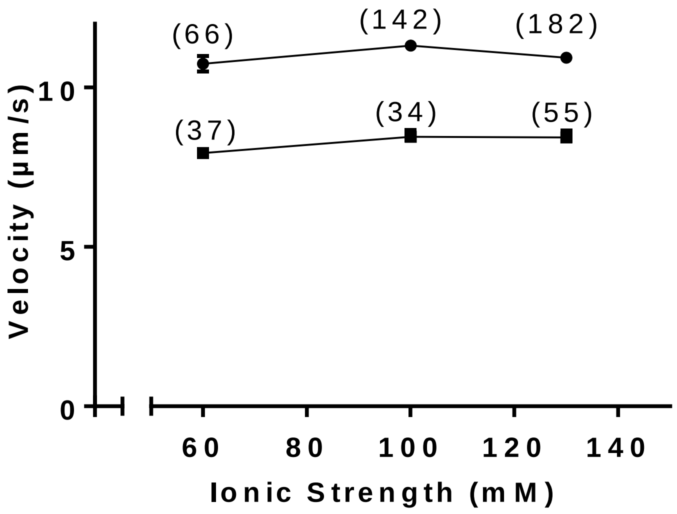

Figure S5. Sliding velocity vs ionic strength of the assay solution using dP-HMM in the absence of blebbistatin. Two different experimental occasions using one HMM preparation with the temperature either 29.8-30.5^{o}C (circles) or 27.2-28.6^{o}C (squares). Data given as mean ± 95 % CI. The numbers in parentheses is the number of filaments studied.

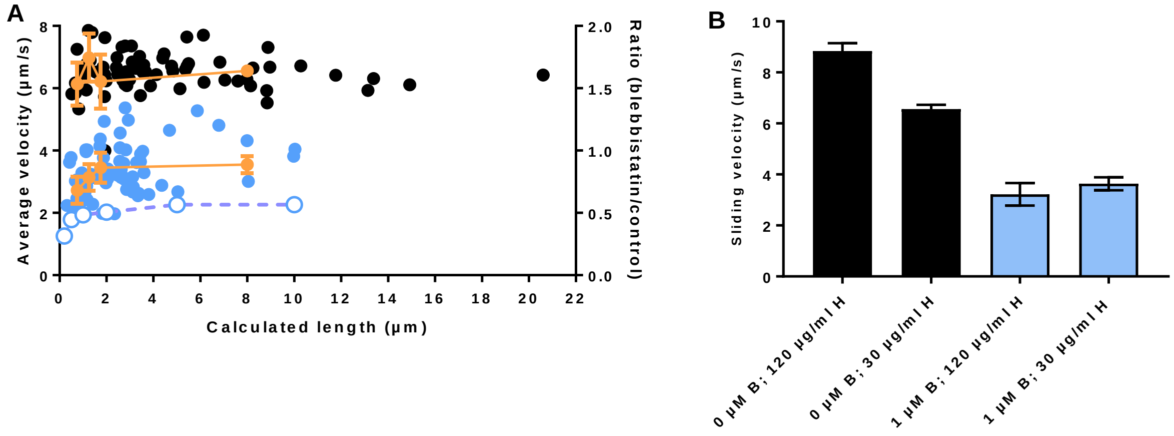

Figure S6. Effect of 1 µM blebbistatin on sliding velocity in the in vitro motility assay – modulation by HMM surface density. A, Velocity vs length plots in an in vitro motility assay after incubation with 30 µg/ml HMM, expected (56) to lower the HMM density on the surface to less than 50 % of that seen after incubation with HMM at 120 µg/ml**.** Data in the absence (black) and presence (blue) of 1 µM S(-) blebbistatin. Orange data points represent mean values ± 95 % CI for ranges of lengths from left to right: 0.5-1.0µm; 1.0-1.5 µm, 1.5-2.0 µm and > 2 µm. Note, lower velocity ratio between blebbistatin and control data for the range 0.5-1 µm compared to lengths > 2 µm. The open blue symbols and dashed line (right axis) gives the ratio between simulated data in the presence and absence of 1 µM blebbistatin (open symbols in main Fig. 2) assuming that the compound lowers k_{P+}'. **B**, Sliding velocity independent of filament length given as mean ± 95 % CI in the absence (black) and presence (blue) of 1 µM S(-) blebbistatin blebbistatin at two different HMM incubation concentration. Note, that reduced HMM incubation concentration lowered sliding velocity in the absence but not in the presence of blebbistatin giving attenuated blebbistatin induced reduction in velocity at the lower HMM incubation concentration. Different myosin preparation than any of those used in main Fig. 1.

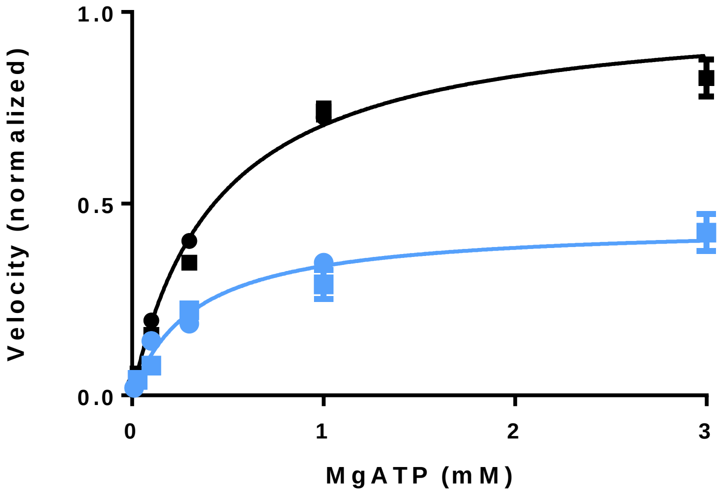

Fig. S7. Actin sliding velocity vs [MgATP] in the presence (blue) and absence (black) of blebbistatin. Data from the same experiments as in main Fig. 2D.Velocity in each experiment was normalized to the velocity at infinite [MgATP] in the absence of blebbistatin as estimated from rectangular hyperbolic fits (Michaelis-Menten type). The absolute value of that velocity value was 12.75 ± 0.35 µm/s (mean ± 95 % CI) in one of the experiments (circles) and 11.66 ± 0.45 µm/s in the other (squares). Curves represent fits of hyperbolic functions to all normalized data with Michaelis-Menten constants V_{Max}= 1.017 ± 0.024 and K_M^v=0.442 ± 0.030 mM under control conditions (black) and V_{Max}= 0.447 ± 0.025 and K_M^v =0.324 ± 0.054 mM in the presence of 1 µM blebbistatin. Temperature: 27.6-30.5 °C (constant to within 1.0 °C in a given experiment).

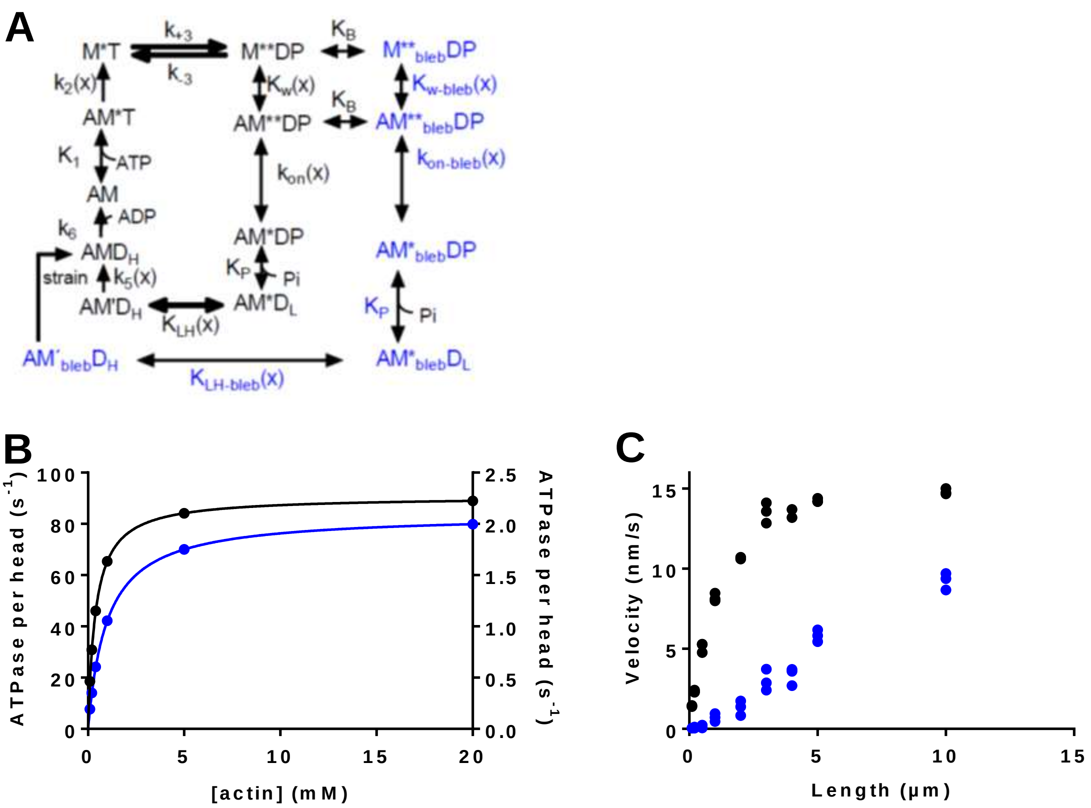

Fig. S8. Simulations using model of Månsson (3). **A**. Kinetic scheme of model adapted with blebbistatin bound states in blue. **B.** Actomyosin ATPase vs actin concentration in solution simulated without blebbistatin (black; left vertical axis) or under the assumption that blebbistatin (saturating concentrations; blue; right axis) reduces the kon(x) rate function 100-fold (to kon-bleb(x)) and the equilibrium constant for the force-generating transition (K_{LH}(x)) more than 5 orders of magnitude. Curves represent fits of hyperbolic functions to the data with Michaelis-Menten constants V_{max}= 90.69 ± 0.01 s^{-1} (mean ± 95 % CI) and K_{ATPase}=0.388 ± 0.0002 mM under control conditions (black; left axis) and V_{max}= 2.096 ± 0.005 s^{-1} and K_{ATPase} =0.987 ± 0.007 mM (blue; right axis) for saturating blebbistatin conditions. **C.** Velocity vs filament length plots simulated without blebbistatin (black) or under the assumption that blebbistatin (blue) reduces both kon(x) and (K_{LH}(x)) as in B. Note appreciable length dependence of the simulated blebbistatin effects on velocity. Blebbistatin concentration assumed to be 2 µM and blebbistatin affinity 5 μM^{-1}. The latter value is five-fold higher than assumed in main Fig. 4 using the expanded version of the model

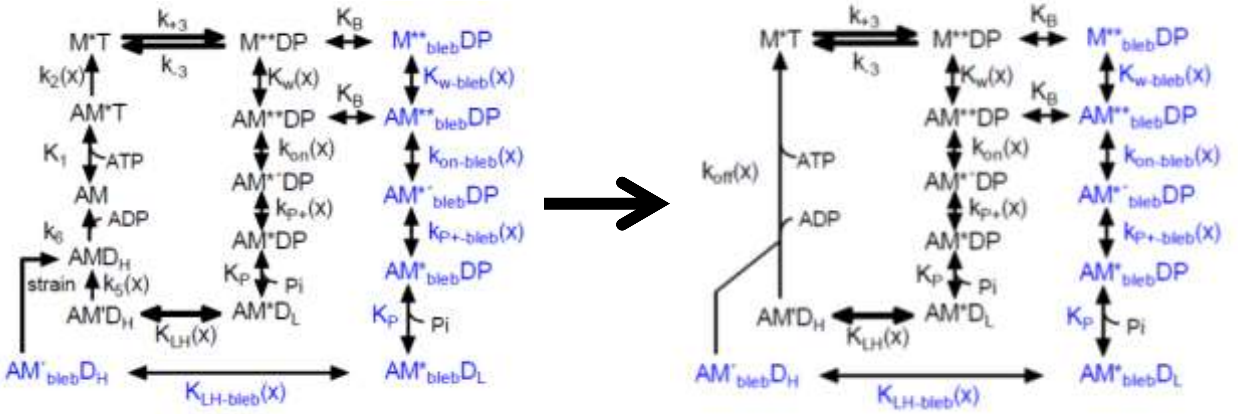

Fig. S9. Reduction of final model (model in Fig. S7 + ideas in (1)) by lumping all rate functions between the AM´D_H and the M*T states into one detachment rate function ($k_{off}(x)$). The reduction is possible because biochemical evidence(41) suggests negligible effect of blebbistatin on that part of the cycle. See further main Fig. 4 for analyses using this model.

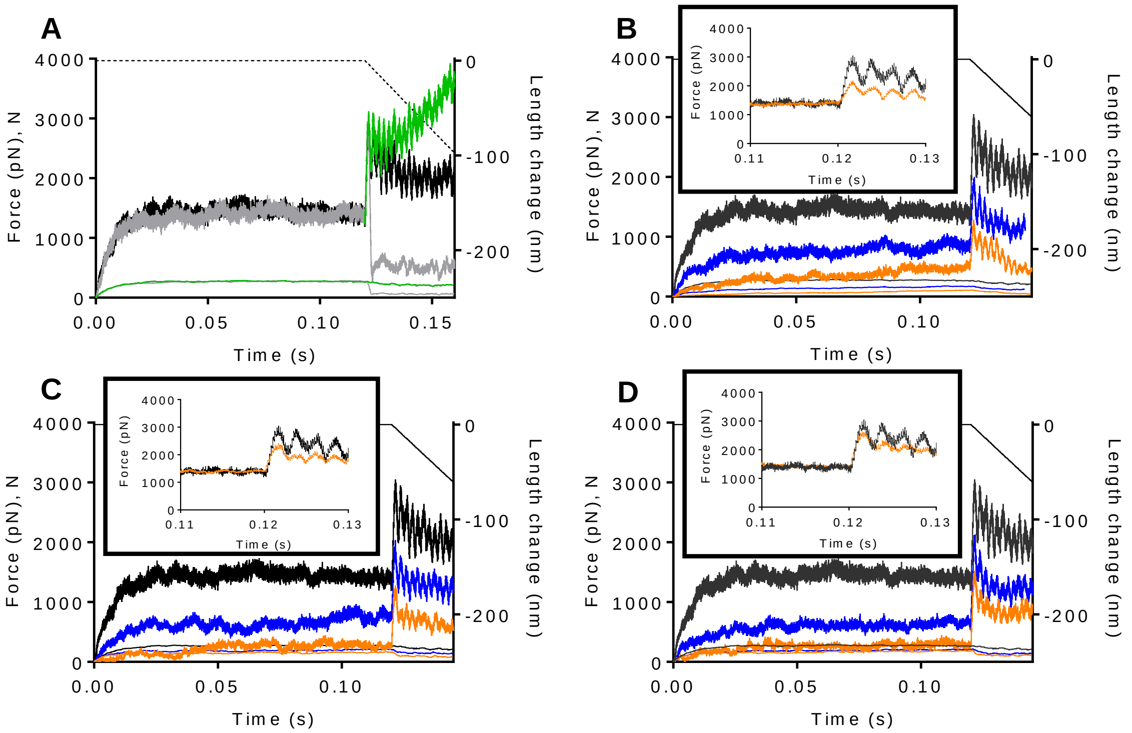

Figure S10. Development of isometric force, number (N) of attached myosin heads and tension responses to stretch during steady-state isometric contraction modeled by Monte-Carlo simulations. Simulated tension responses (thick full lines; left vertical axis) and number of attached cross-bridges (thin full lines; left axis) are shown in response to stretch (dashed black line; right axis). **A.** Control conditions under different assumptions. Grey: no “slippage” between sites (no rapid detachment-reattachment events; Eq. S14). Black: Same parameter values as for grey trace but slippage assumed to occur; Green: Same parameter values as for black trace, but effect of a linear parallel elastic element, simulating titin effects, also included. In the rest of the panels the parallel elastic element is omitted but slippage is included in the model. **B.** Simulated tension responses at 0 (black), 2 µM blebbistatin (blue) and 10 µM blebbistatin (orange) on the assumption that blebbistatin reduces $k_{on+}(x)$ to a degree, sufficient to account for reduced V_{max} of the actomyosin ATPase. **C.** Simulated tension responses at 0 (black), 2 µM blebbistatin (blue) and 10 µM blebbistatin (orange) on the assumption that blebbistatin reduces k_{P+} 650-fold, sufficient to account for reduced V_{max} of the actomyosin ATPase. **D.** Simulated tension responses as in C on the assumption that blebbistatin reduces k_{P+} 650-fold but that it also reduces the difference between the free energy minima of the AM*D_L and the AM´D_H states from 14 to 2.5 k_BT. The insets in B-D show the tension responses to stretch on an expanded time scale for the control condition and in the presence of 10 µM blebbistatin where the latter trace is shifted upwards to make the isometric force level coincide with that under control conditions. The Monte-Carlo simulations were implemented for the model in main Fig. 3A assuming that a 20 µm long actin filament interacts with 5000 myosin heads µm^{-2} on an in vitro motility assay surface. The filament is first assumed to be held isometrically and then (at time 0.05 s) being stretched at 2500 nm/s. Simulations were performed assuming parameter values as at 25-30 ^{o}C (Tables S1-S2).

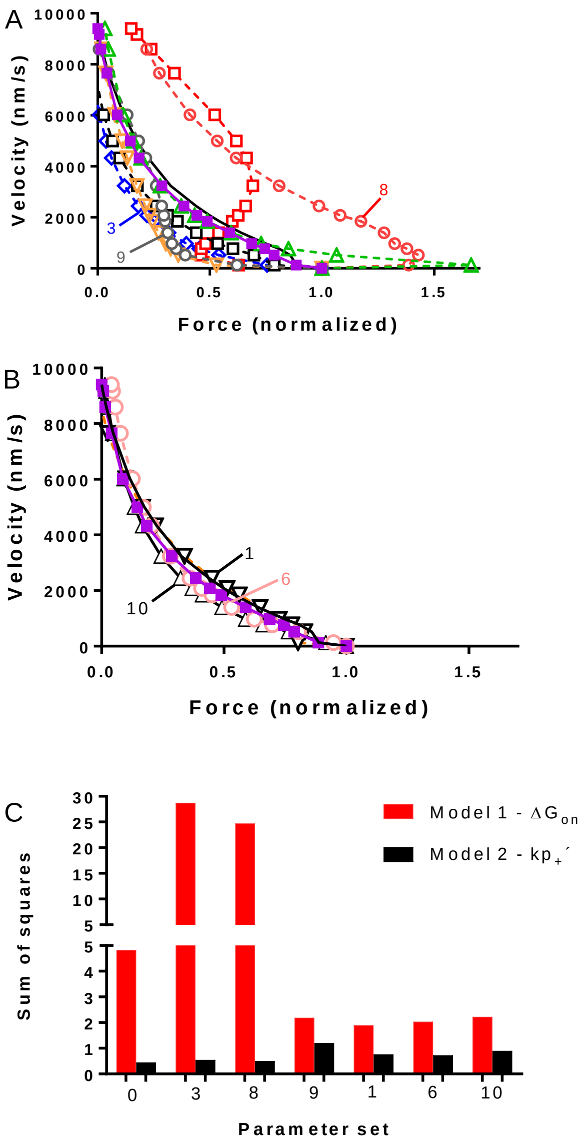

Figure S11. Simulation results obtained by random 25 % increase or 25 % decrease of parameter values (Table S5) compared to parameter values from the literature (Tables S1 and S2). A. Simulated force-velocity data in the absence of blebbistatin using parameter values that give bad fits (open symbols and dashed line) to the experimental data (purple). Full black line without symbols represent simulated results using the parameter values in Tables S1 and S2. The simulations correspond to the parameter values which are given in red font in Table S5. The numbers correspond to parameter set in Table S5. **B.** Simulated force velocity data as in A but using parameter values (black font in Table S5) that give good fits. Corresponding color coding as in A for purple symbols and line and black line without symbols. The numbers correspond to parameter set in C. **C**. Sum of squared deviations between experimental data and simulated data for variables shown in Table S4. The simulations were performed for all sets of parameter values (sets 1,6,10) from Table S5 that give good fits to force-velocity data (Fig. S11A) and for a random selection of three sets of parameter values (sets 3,8,9 in Table S5) giving poor fits to the force velocity data (Fig. S11B). In these sum of squares values contributions for the variable K_M^V was not included. The sum of squares for the standard parameter values are also shown (parameter set 0), including contributions for the variable K_M^V.

Supplementary References

1. Llinas, P., T. Isabet, L. Song, V. Ropars, B. Zong, H. Benisty, S. Sirigu, C. Morris, C. Kikuti, D. Safer, H. L. Sweeney, and A. Houdusse. 2015. How actin initiates the motor activity of Myosin. Dev Cell 33:401-412.
2. Eisenberg, E., T. L. Hill, and Y. Chen. 1980. Cross-bridge model of muscle contraction. Quantitative analysis. Biophys. J. 29:195-227.
3. Mansson, A. 2016. Actomyosin based contraction: one mechanokinetic model from single molecules to muscle? J. Muscle Res. Cell Motil. 37:181-194.
4. Mansson, A. 2010. Actomyosin-ADP states, inter-head cooperativity and the force-velocity relation of skeletal muscle. Biophys. J. 98:1237-1246.
5. Nyitrai, M., and M. A. Geeves. 2004. Adenosine diphosphate and strain sensitivity in myosin motors. Philos. Trans. R. Soc. Lond. B. Biol. Sci. 359:1867-1877.
6. Capitanio, M., M. Canepari, P. Cacciafesta, V. Lombardi, R. Cicchi, M. Maffei, F. S. Pavone, and R. Bottinelli. 2006. Two independent mechanical events in the interaction cycle of skeletal muscle myosin with actin. Proc. Natl. Acad. Sci. U. S. A. 103:87-92.
7. Albet-Torres, N., M. J. Bloemink, T. Barman, R. Candau, K. Frölander, M. A. Geeves, K. Golker, C. Herrmann, C. Lionne, C. Piperio, S. Schmitz, C. Veigel, and A. Månsson. 2009. Drug effect unveils inter-head cooperativity and strain-dependent ADP release in fast skeletal actomyosin. J. Biol. Chem. 284:22926–22937.
8. Persson, M., E. Bengtsson, L. ten Siethoff, and A. Mansson. 2013. Nonlinear cross-bridge elasticity and post-power-stroke events in fast skeletal muscle actomyosin. Biophys. J. 105:1871-1881.
9. Bell, G. I. 1978. Models for the specific adhesion of cells to cells. Science 200:618-627.
10. Curtin, N. A., and R. E. Davies. 1975. Very high tension with very little ATP breakdown by active skeletal muscle. J. Mechanochem. Cell Motil. 3:147-154.
11. Lombardi, V., and G. Piazzesi. 1990. The contractile response during steady lengthening of stimulated frog muscle fibres. J. Physiol. (Lond). 431:141-171.
12. Mansson, A. 1994. The tension response to stretch of intact skeletal muscle fibres of the frog at varied tonicity of the extracellular medium. J. Muscle Res. Cell Motil. 15:145-157.
13. Brunello, E., M. Reconditi, R. Elangovan, M. Linari, Y. B. Sun, T. Narayanan, P. Panine, G. Piazzesi, M. Irving, and V. Lombardi. 2007. Skeletal muscle resists stretch by rapid binding of the second motor domain of myosin to actin. Proc. Natl. Acad. Sci. U. S. A. 104:20114-20119.
14. Caremani, M., L. Melli, M. Dolfi, V. Lombardi, and M. Linari. 2013. The working stroke of the myosin II motor in muscle is not tightly coupled to release of orthophosphate from its active site. J Physiol 591:5187-5205.
15. Joumaa, V., and W. Herzog. 2014. Calcium sensitivity of residual force enhancement in rabbit skinned fibers. Am. J. Physiol. Cell Physiol. 307:C395-401.
16. Rassier, D. E., and I. Pavlov. 2012. Force produced by isolated sarcomeres and half-sarcomeres after an imposed stretch. Am. J. Physiol. Cell Physiol. 302:C240-248.
17. Edman, K. A. 2012. Residual force enhancement after stretch in striated muscle. A consequence of increased myofilament overlap? J Physiol 590:1339-1345.
18. Campbell, S. G., P. C. Hatfield, and K. S. Campbell. 2011. A mathematical model of muscle containing heterogeneous half-sarcomeres exhibits residual force enhancement. PLoS Comp Biol. 7:e1002156.
19. Duke, T. A. 1999. Molecular model of muscle contraction. Proc. Natl. Acad. Sci. U. S. A. 96:2770-2775.

20. Nishizaka, T., R. Seo, H. Tadakuma, K. Kinosita, Jr., and S. Ishiwata. 2000. Characterization of single actomyosin rigor bonds: load dependence of lifetime and mechanical properties. Biophys. J. 79:962-974.
21. Capitanio, M., M. Canepari, M. Maffei, D. Beneventi, C. Monico, F. Vanzi, R. Bottinelli, and F. S. Pavone. 2012. Ultrafast force-clamp spectroscopy of single molecules reveals load dependence of myosin working stroke. Nature methods 9:1013-1019.
22. Brenner, B., and E. Eisenberg. 1986. Rate of force generation in muscle: correlation with actomyosin ATPase activity in solution. Proc. Natl. Acad. Sci. U. S. A. 83:3542-3546.
23. Woledge, R. C., N. A. Curtin, and E. Homsher. 1985. Energetic aspects of muscle contraction. Academic Press, London.
24. Offer, G., and K. W. Ranatunga. 2015. The endothermic ATP hydrolysis and crossbridge attachment steps drive the increase of force with temperature in isometric and shortening muscle. J Physiol 593:1997-2016.
25. Sleep, J., M. Irving, and K. Burton. 2005. The ATP hydrolysis and phosphate release steps control the time course of force development in rabbit skeletal muscle. J Physiol 563:671-687.
26. Ranatunga, K. W. 2010. Force and power generating mechanism(s) in active muscle as revealed from temperature perturbation studies. J Physiol 588:3657-3670.
27. Zhao, Y., and M. Kawai. 1994. Kinetic and thermodynamic studies of the cross-bridge cycle in rabbit psoas muscle fibers. Biophys. J. 67:1655-1668.
28. Linari, M., M. Caremani, C. Piperio, P. Brandt, and V. Lombardi. 2007. Stiffness and fraction of Myosin motors responsible for active force in permeabilized muscle fibers from rabbit psoas. Biophys. J. 92:2476-2490.
29. Nyitrai, M., R. Rossi, N. Adamek, M. A. Pellegrino, R. Bottinelli, and M. A. Geeves. 2006. What limits the velocity of fast-skeletal muscle contraction in mammals? J. Mol. Biol. 355:432-442.
30. Roots, H., G. J. Pinniger, G. W. Offer, and K. W. Ranatunga. 2012. Mechanism of force enhancement during and after lengthening of active muscle: a temperature dependence study. J. Muscle Res. Cell Motil. 33:313-325.
31. Ranatunga, K. W., and M. E. Coupland. 2010. Crossbridge mechanism(s) examined by temperature perturbation studies on muscle. Adv. Exp. Med. Biol. 682:247-266.
32. Hook, P., and L. Larsson. 2000. Actomyosin interactions in a novel single muscle fiber in vitro motility assay. J. Muscle Res. Cell Motil. 21:357-365.
33. Pardee, J. D., and J. A. Spudich. 1982. Purification of muscle actin. Methods Cell Biol. 24:271-289.
34. Kron, S. J., Y. Y. Toyoshima, T. Q. Uyeda, and J. A. Spudich. 1991. Assays for actin sliding movement over myosin-coated surfaces. Methods Enzymol. 196:399-416.
35. Perrie, W. T., and S. V. Perry. 1970. An electrophoretic study of the low-molecular-weight components of myosin. Biochem. J. 119:31-38.
36. Wilson, C., N. Naber, E. Pate, and R. Cooke. 2014. The myosin inhibitor blebbistatin stabilizes the super-relaxed state in skeletal muscle. Biophys. J. 107:1637-1646.
37. Minozzo, F. C., and D. E. Rassier. 2010. Effects of blebbistatin and Ca2+ concentration on force produced during stretch of skeletal muscle fibers. Am. J. Physiol. Cell Physiol. 299:C1127-1135.
38. Sakamoto, T., J. Limouze, C. A. Combs, A. F. Straight, and J. R. Sellers. 2005. Blebbistatin, a myosin II inhibitor, is photoinactivated by blue light. Biochemistry. 44:584-588.

39. Getz, E. B., R. Cooke, and S. L. Lehman. 1998. Phase transition in force during ramp stretches of skeletal muscle. Biophys.J. 75:2971-2983.
40. Lard, M., L. ten Siethoff, J. Generosi, M. Persson, H. Linke, and A. Mansson. 2015. Nanowire-imposed geometrical control in studies of actomyosin motor function. IEEE trans. Nanobiosci. 14:289-297.
41. Kovacs, M., J. Toth, C. Hetenyi, A. Malnasi-Csizmadia, and J. R. Sellers. 2004. Mechanism of blebbistatin inhibition of myosin II. J. Biol. Chem. 279:35557-35563.
42. Gillespie, D. T. 1976. A general method for numerically simulating the stochastic time evolution of coupled chemical reactions. J. Comp. Phys. 22:403-434.
43. Kaya, M., and H. Higuchi. 2010. Nonlinear elasticity and an 8-nm working stroke of single myosin molecules in myofilaments. Science 329:686-689.
44. Brenner, B., L. C. Yu, L. E. Greene, E. Eisenberg, and M. Schoenberg. 1986. Ca2+-sensitive cross-bridge dissociation in the presence of magnesium pyrophosphate in skinned rabbit psoas fibers. Biophys. J. 50:1101-1108.
45. Dantzig, J. A., Y. E. Goldman, N. C. Millar, J. Lacktis, and E. Homsher. 1992. Reversal of the cross-bridge force-generating transition by photogeneration of phosphate in rabbit psoas muscle fibres. J Physiol 451:247-278.
46. Karatzaferi, C., M. K. Chinn, and R. Cooke. 2004. The force exerted by a muscle cross-bridge depends directly on the strength of the actomyosin bond. Biophys. J. 87:2532-2544.
47. Pate, E., and R. Cooke. 1989. A model of crossbridge action: the effects of ATP, ADP and Pi. J. Muscle Res. Cell Motil. 10:181-196.
48. Debold, E. P., M. A. Turner, J. C. Stout, and S. Walcott. 2011. Phosphate enhances myosin-powered actin filament velocity under acidic conditions in a motility assay. Am J Physiol Regul Integr Comp Physiol 300:R1401-1408.
49. Mansson, A., J. Morner, and K. A. Edman. 1989. Effects of amrinone on twitch, tetanus and shortening kinetics in mammalian skeletal muscle. Acta Physiol. Scand. 136:37-45.
50. Hill, A. V. 1938. The heat of shortening and the dynamic constants of muscle. Proc. Royal Soc. B 136–195 126:136-195.
51. Edman, K. A., A. Mansson, and C. Caputo. 1997. The biphasic force-velocity relationship in frog muscle fibres and its evaluation in terms of cross-bridge function J. Physiol. (Lond). 503:141-156.
52. Tesi, C., F. Colomo, N. Piroddi, and C. Poggesi. 2002. Characterization of the cross-bridge force-generating step using inorganic phosphate and BDM in myofibrils from rabbit skeletal muscles. J Physiol 541:187-199.
53. Coupland, M. E., E. Puchert, and K. W. Ranatunga. 2001. Temperature dependence of active tension in mammalian (rabbit psoas) muscle fibres: effect of inorganic phosphate. J Physiol 536:879-891.
54. Cooke, R., and E. Pate. 1985. The effects of ADP and phosphate on the contraction of muscle fibers. Biophys. J. 48:789-798.
55. Wagner, P. D. 1984. Effect of skeletal muscle myosin light chain 2 on the Ca2+-sensitive interaction of myosin and heavy meromyosin with regulated actin. Biochemistry. 23:5950-5956.
56. Bengtsson, E., M. Persson, M. A. Rahman, S. Kumar, H. Takatsuki, and A. Mansson. 2016. Myosin-Induced Gliding Patterns at Varied [MgATP] Unveil a Dynamic Actin Filament. Biophys. J. 111:1465-1477.
