# Supplementary material for: Blebbistatin Effects Expose Hidden Secrets in the Force-Generating Cycle of Actin and Myosin
Source: Biophys J. 2018 Jul 17;115(2):386–97. doi: 10.1016/j.bpj.2018.05.037 (PMC6050972; doi:10.1016/j.bpj.2018.05.037)
Supplement: Document S2. Article plus Supporting Material [file mmc2.pdf]

Biophysical Journal
Article

Biophysical Society

Blebbistatin Effects Expose Hidden Secrets in the Force-Generating Cycle of Actin and Myosin

Mohammad A. Rahman,[1] Marko Ušaj,[1] Dilson E. Rassier,[2] and Alf Månsson[1,*]
[1]Department of Chemistry and Biomedical Sciences, Linnaeus University, Kalmar, Sweden and [2]Department of Kinesiology and Physical Education, McGill University, Montreal, Canada

ABSTRACT Cyclic interactions between myosin II motors and actin filaments driven by ATP turnover underlie muscle contraction and have key roles in the motility of nonmuscle cells. A remaining enigma in the understanding of this interaction is the relationship between the force-generating structural change and the release of the ATP-hydrolysis product, inorganic phosphate (Pi), from the active site of myosin. Here, we use the small molecular compound blebbistatin to probe otherwise hidden states and transitions in this process. Different hypotheses for the Pi release mechanism are tested by interpreting experimental results from in vitro motility assays and isolated muscle fibers in terms of mechanokinetic actomyosin models. The data fit with ideas that actomyosin force generation is preceded by Pi release, which in turn is preceded by two serial transitions after/coincident with cross-bridge attachment. Blebbistatin changes the rate limitation of the cycle from the first to the second of these transitions, uncovering functional roles of an otherwise short-lived pre-power stroke state that has been implicated by structural data.

INTRODUCTION

Muscle contraction results from cyclic interactions of myosin II motor domains with actin filaments driven by ATP turnover. This process underlies bodily movements powered by skeletal muscle, pumping of blood by the heart, and a range of homeostatic mechanisms governed by smooth muscle. Additionally, nonmuscle myosin II and actin have pivotal roles in cell motility and thereby in functions associated with the immune system, synaptic plasticity, and cell division (1). Therefore, disturbed myosin II function is central to a range of diseases (2–4) as well as to functional decline during aging (5). Such characteristics have motivated the development of myosin-active small molecular compounds for therapeutic use (2,3,6–8). However, the insights into diseases as well as the associated drug discovery processes are hampered by remaining enigmas in the fundamental understanding of the force-generating cross-bridge cycle between myosin II and actin. Particularly, the relationship between actomyosin cross-bridge formation, phosphate (Pi) release from the myosin active site, and the force-generating structural change is poorly understood with a range of conflicting views (9–21) (reviewed in (22–24)). Outstanding questions include but are not limited to the following. 1) Does Pi release occur before (11,19,20,25) or after (12,15,17,26) force generation? Are Pi release and force generation loosely coupled (14)? Are branched kinetic paths necessary to account for Pi effects (27)? 2) Which transition(s) is/are rate limiting for Pi release, force generation, and actomyosin ATPase (9,13,23,28,29)? 3) How does the fast force generation in response to rapid length changes (30) fit into the picture (11,23,31)? Naturally, these questions are of highest fundamental importance because the force-generating process and its relationship to rate-limiting transitions as well as to the kinetic step with the largest drop in free energy (Pi release) is at the core of the energy transduction process (19,23,32). Accordingly, several drugs and toxins (33–45) affect transitions and states in this phase of the cycle with substantial effects both on the actomyosin ATPase in solution, force generation, and shortening velocity. However, consistent with the limited insight into the process, it has been difficult to arrive at a full understanding of the mechanism of action of a range of myosin active substances (35,38,39,46,47).

One small molecular compound that has been extensively studied is the myosin II selective inhibitor blebbistatin, a 1-phenyl-2-pyrrolidinone derivative (40–43,48) (Fig. 1 *A*). Because of its incompletely understood effects on Pi release, more detailed studies have the potential to expose hidden states and transitions that cannot be readily probed otherwise (cf. (33)). One advantage of using chemical substances

Submitted February 12, 2018, and accepted for publication May 30, 2018.
*Correspondence: alf.mansson@lnu.se
Editor: Steven Rosenfeld.
https://doi.org/10.1016/j.bpj.2018.05.037
© 2018 Biophysical Society.
This is an open access article under the CC BY license (http://creativecommons.org/licenses/by/4.0/).

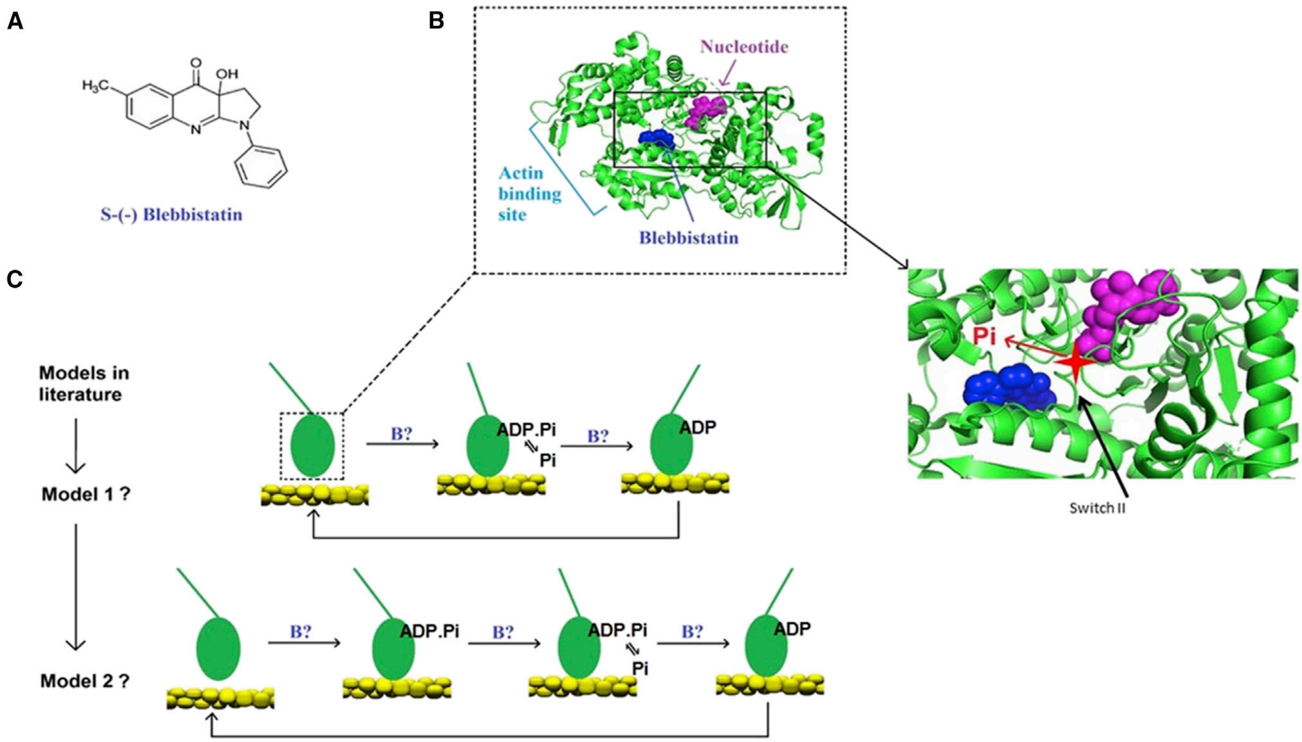

FIGURE 1 Blebbistatin probing the mechanism for phosphate release and force generation. (*A*) The molecular structure of S-(−) blebbistatin is shown. (*B*) The motor domain of dictyostelium myosin II (48) shows blebbistatin at its binding site and ADP at the active site. The further enlarged image to the right illustrates the proposed molecular model for phosphate release (inorganic phosphate molecule indicated by *star*). The figure was produced by PyMOL 2.0 using the deposited structure with Protein Data Bank: 1YV3 showing the myosin II motor domain with blebbistatin and ADP. Vanadate, magnesium and 1,2-ethanediol present in the deposited structure are excluded. (*C*) Two models (model 1 (29) and model 2 (19,24)) were selected for final testing based on well-founded arguments from previous experimental and theoretical studies, as considered in detail in the Discussion. The schematic drawing illustrates the myosin motor domain either bound or not bound to actin (*double helical structure*) and with different lever arm (*straight lines*) positions. Hypothetical blebbistatin-affected transitions tested below are indicated by "B?." The dashed rectangle encloses the myosin motor domain illustrated in molecular detail in (*B*). To see this figure in color, go online.

instead of specific mutations for functional studies of this type is that it facilitates the use of a range of experimental preparations, from isolated single molecules to contractile cells investigated in situ. Substances of particular value in this context are those, such as blebbistatin, whose binding site on myosin is well characterized (Fig. 1 *B*). This compound and its derivatives (e.g., (49,50)) are known to selectively inhibit the actin-activated ATPase of myosin II (40–43), causing accumulation of cross-bridges in a pre-power-stroke state with ADP and inorganic phosphate (Pi) at the active site (47,51). Whereas this mechanism of action appears deceivingly simple, the full picture is complex. Particularly, as outlined above, the affected part of the acto-myosin cross-bridge cycle remains poorly understood despite intense investigations (9–19,21). Second, a blebbistatin-induced decrease in the unloaded shortening velocity in muscle cells only occurs when the myosin regulatory light chains (RLCs) are phosphorylated, whereas the effect on isometric force is independent of phosphorylation (52). Finally, blebbistatin has also been found (44) to stabilize the start of the power-stroke actomyosin state without Pi at the active site.

Here, we performed in vitro motility assay experiments and mechanical studies of muscle fibers in the presence and absence of blebbistatin to allow testing of different mechanokinetic models for the actomyosin cross-bridge cycle (Fig. 1 *C*). These models connect the states and transitions found in biochemical and structural studies to contractile function. The simplest model tested is identical to that in (29). Whereas this model (model 1 in Fig. 1 *C*) accounts for a wide range of experimental data in the absence of blebbistatin, it failed to fully account for the blebbistatin effects. We therefore extended model 1 by introducing states and transitions similar to those proposed by Llinas et al. (19) based on structural and reverse genetics studies of several myosin classes. The expanded model (model 2 in Fig. 1 *C*) accounts for all our new experimental results and, to the best of our knowledge, also for experimental blebbistatin data reported elsewhere (see Discussion). Importantly, therefore, our results corroborate the functional significance of a sequence of events from cross-bridge attachment to phosphate release and force generation proposed recently (18,19,25). Furthermore, the results give a solid platform for addressing all the outstanding questions mentioned

above as well as for analyzing the effects of other relevant drugs, toxins, and mutations.

MATERIALS AND METHODS

Ethical statement

Rabbits were kept and sacrificed using procedures approved by the Regional Ethical Committee for Animal Experiments in Linköping, Sweden (reference number 73-14).

In vitro motility assays

In vitro motility assays were performed as described previously using actin (53) and heavy meromyosin (HMM) (54) from fast rabbit muscle. Also, flow cells with silanized surfaces, incubation conditions, assay solutions, recording of motility assay data, and subsequent analysis were described earlier (55). The flow cells were incubated with HMM at 120 or 30 μg/mL (Fig. S6) for 2–5 min. The assay solution had [MgATP] = 1 mM, an ionic strength of 130 mM, and contained 0.64% methylcellulose unless stated otherwise. Temperature was 27.6–30.5°C, and pH = 7.4. HMM with and without phosphorylated RLCs were prepared essentially as described in (56) and characterized using urea gels (57). Blebbistatin was either (±)-blebbistatin or S-(−) blebbistatin.

Muscle fiber experiments

Experiments using skinned skeletal muscle fibers from fast rabbit muscle were performed as described previously (51). Fibers were activated in the presence or absence of S-(−) blebbistatin (n = 16). All experiments were performed at 5°C, and the initial sarcomere length was adjusted to ~2.5 μm (optimal length, Lo) before fiber activation.

Modeling

The mechanokinetic model was developed from that in (29) with the addition of all blebbistatin-bound states and two blebbistatin-free states (AM*'DP and AM*DP). The parameters that define the model for temperatures in the range of 25–30°C are given in Tables S1 and S2. These parameter values are essentially the same as those used previously (29) except for those related to the AM*'DP and the AM*DP states. Unless otherwise stated, the model predictions were derived by Monte Carlo simulations using the Gillespie algorithm (58) as described previously (29). In some cases (e.g., actin-activated solution ATPase and some control simulations; Figs. S1 and S11), predictions were obtained by solving differential equations in state probabilities (29). Because experiments on skinned muscle fibers were conducted at 5°C, we also performed simple analysis based on changes in key parameters to values corresponding to those at 5°C (Table S3).

More methodological details, including details about the modeling, are given in the Supporting Materials and Methods.

RESULTS

Blebbistatin reduced the sliding velocity in the in vitro motility assay in a concentration-dependent manner (Fig. 2 A). The half-maximal inhibition occurred in the range of ~1–5 μM blebbistatin for three different batches of HMM (three different myosin preparations) and three different batches of blebbistatin with a mixture of (+) and (−) enantiomers. In two experiments, we also tested pure S-(−) blebbistatin, generally claimed to be the active enantiomer (40). The half-inhibitory concentration in this case was <1 μM for one HMM batch and ~3 μM for the other

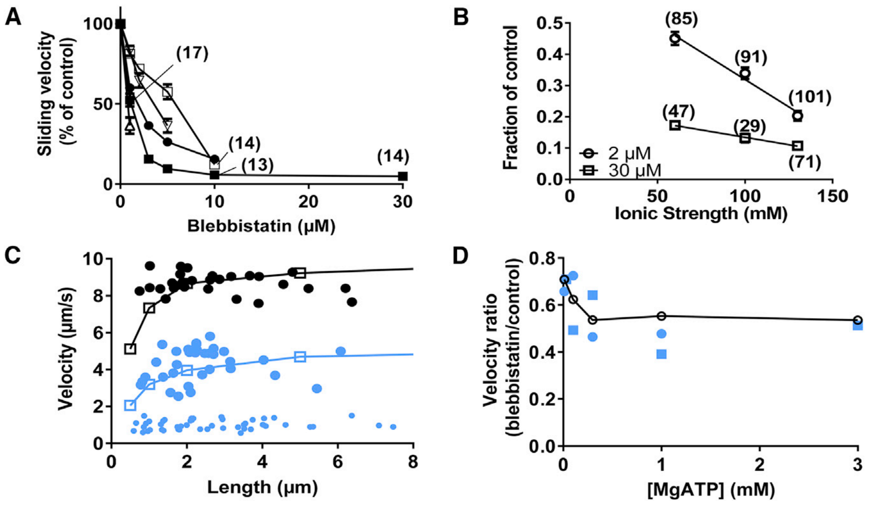

FIGURE 2 Effects of blebbistatin on sliding velocities in the in vitro motility assay. (A) Concentration-response curve for effect of blebbistatin on sliding velocity at 130 mM ionic strength (1 mM MgATP) is shown using HMM with dephosphorylated regulatory light chains (dP-HMM). Each data point represents two to five flow cells and more than 20 filaments unless indicated in parentheses. Data from three different blebbistatin batches with mixed enantiomers are illustrated by different symbols, and two different dP-HMM preparations are illustrated by filled and open symbols, respectively. Two experiments using pure S-(−) blebbistatin are illustrated by open triangles. The sliding velocity in the control solution (0 μM blebbistatin) varied between 9.8 and 11.8 μm/s in the different experiments. (B) Blebbistatin effects on sliding velocity at different ionic strengths (1 mM MgATP) are shown. Fractional velocity at either 2 μM ($circles$) or 30 μM ($squares$) blebbistatin is shown, where velocity data are normalized to the velocity at the respective ionic strength in the absence of blebbistatin (control). In the absence of blebbistatin, the mean velocity was constant with changes in ionic strengths to ± 3%. (C) Velocity versus filament length is shown in the absence of blebbistatin ($black$) or in the presence of 10 μM blebbistatin ($small$ $bright$ $symbols$) or a blebbistatin concentration (5 μM; big $bright$ $symbols$) approximately reducing the velocity to half. Filled symbols show experimental data. Open symbols connected by lines show simulated data assuming three independent myosin-binding sites per 36 nm of the actin filament. The blebbistatin effect was modeled using a model in which k_{P+}' was reduced from 1000 to 1.5 s^{-1}, as illustrated in greater detail in Fig. 4 below (1 mM MgATP; ionic strength 130 mM). (D) Sliding velocity vs. [MgATP] in the presence of 1 μM blebbistatin is shown as fraction of velocity in the absence of the blebbistatin ($bright$ $symbols$). Data points from experiment with mixed blebbistatin enantiomer ($filled$ $circles$) or pure S-(−) blebbistatin ($filled$ $squares$) are shown. Different myosin preparations were used for these two experiments. The number of filaments for each data point was >20 in all cases. Open symbols connected by line represent blebbistatin effects modeled using a model in which k_{P+}' was reduced from 1000 to 1.5 s^{-1}. See further details in Fig. 4. Temperature was 27.6–30.5°C (constant to within 1.0°C in a given experiment). Error bars had 95% confidence intervals. To see this figure in color, go online.

(Fig. 2 *A*). The sliding velocity plateaued at a nonzero value at blebbistatin concentrations higher than 7–10 μM. In tests below, we titrated the concentration of each specific blebbistatin batch to achieve ~50% blebbistatin-induced reduction in velocity compared to the control. Two of the three HMM preparations in Fig. 2 had dephosphorylated RLCs (dephosphorylated (dP)-HMM), as verified by Urea gels (Fig. S2). The third HMM batch was prepared to give dP-HMM, but the phosphorylation status was not verified in a gel. Importantly, however, partial phosphorylation of the RLCs did not alter the blebbistatin effects (Figs. S2–S4).

Biochemical assays suggest that blebbistatin inhibits a transition that traps myosin heads in a weakly actin-attached state (41). A blebbistatin-induced reduction in force is a self-evident consequence of such a mechanism. However, a reduction in maximal sliding velocity is not because velocity is believed to be governed primarily by the cross-bridge detachment rate at the end of the power stroke (59–61). Nevertheless, one possibility to account for lowered velocity as a consequence of increased population of weakly bound states is if blebbistatin enhances actin affinity in these states, leading to increased friction upon relative sliding of actin and myosin (28,62). If such a mechanism is at work, the effect of blebbistatin on sliding velocity would be increased by lowering ionic strength because the actin affinity of weak-binding states is approximately doubled for a 20 mM reduction in ionic strength (63). However, we found (Fig. 2 *B*) a reduced effect of blebbistatin on velocity when ionic strength was lowered in the range from 130 to 60 mM, arguing against the relevance of frictional forces. This idea is consistent with the lack of change in velocity when the ionic strength was varied in the range of 60–130 mM in the absence of blebbistatin (Fig. S5). The attenuation of the blebbistatin effect at reduced ionic strength is briefly considered in Supporting Materials and Methods.

One other possibility to account for blebbistatin-induced reduction in velocity on the basis of reduced attachment of cross-bridges into force-producing states is if the velocity reduction primarily reflects the behavior of the shortest actin filaments in the in vitro motility assay. Such filaments are propelled by a few myosin motors also in the absence of blebbistatin (29,64), and a further reduction in this number by reduced attachment rate would severely reduce the propulsion speed. However, our results show blebbistatin effects on sliding velocity that are virtually independent of filament length (Fig. 2 *C*). There was no tendency for reduced effect of the compound even for the longest filaments observed. Similar results were found whether we used motility assays without (Fig. 2 *C*) or with (Fig. S4) blocking actin to largely eliminate the effects of rigor-like heads (cf. (55)). A lowered HMM density on the surface was achieved in one experiment by reduced HMM incubation concentration from 120 to 30 μg/mL (Fig. S6). Under these conditions, the blebbistatin effect on velocity was slightly higher for the shortest filaments (1 μm) compared to the longest filaments observed. However, overall the reduced HMM density did not enhance the blebbistatin effect when all filament lengths were considered. The findings argue further against ideas that lowering the cross-bridge attachment rate plays any appreciable role in the effect of blebbistatin on velocity.

For further insight into the mechanisms underlying the blebbistatin effects, we studied the relationship between [MgATP] and velocity because this relationship is influenced by kinetic parameters of the cross-bridge cycle as well as by the myosin step length (65). The main finding is that the blebbistatin effect on velocity was lower at the lowest MgATP concentrations (<0.1 mM) studied (Fig. 2 *D*). This effect was associated with reduced MgATP concentration ($K_M{}^v$) for half-maximal velocity in the presence of 1 μM blebbistatin (Fig. S7). Biochemical kinetics suggests limited effects of blebbistatin on cross-bridge detachment rate at the end of the power stroke (41). We therefore conclude that blebbistatin causes accumulation of cross-bridges in a pre-power-stroke attached state that acts as a brake on cross-bridge sliding. Such a mechanism lowers $K_M{}^v$ because it reduces velocity particularly at high [MgATP] when there are very few braking cross-bridges in the MgATP-free Actin-Myosin (AM) (rigor) state. The latter cross-bridge state, on the other hand, has a dominating role in limiting velocity at low [MgATP], both in the presence and absence of blebbistatin, explaining the low effect of blebbistatin in this MgATP concentration range.

We also investigated the effects of blebbistatin on maximal isometric force and on the resistance to stretch in lengthening contractions of skinned rabbit psoas muscle cells (Fig. 3 *A*). The experiments were performed at 5°C, a temperature with little blebbistatin-induced myosin head ordering on the thick filament backbone, similar to the effects of RLC phosphorylation (66). We found a concentration-dependent reduction in isometric force and force during stretch, expanding previous findings (51) with 5 μM blebbistatin. The typical phases of the tension response to stretch during active contraction were maintained at all blebbistatin concentrations tested. These phases include a fast increase in force concomitant with half-sarcomere extension of a few nanometers, and a subsequent slow increase (67–69). The first phase is commonly attributed to the elastic behavior of the cross-bridges, and the second phase to changes in the occupational fraction of cross-bridges in the pre- and postpower-stroke states (51,69,70). The second phase may also reflect contribution of parallel elastic elements, e.g., those attributed to the stretching of titin (71). The transition between the two phases is marked by a change in slope of the force rise at a critical force (F_c), commonly associated with the mechanical detachment of cross-bridges from actin.

In Fig. 3, *B* and *C* the sliding velocity, the isometric force, and F_c are all plotted against the blebbistatin concentration as fractions of the values in the absence of the compound.

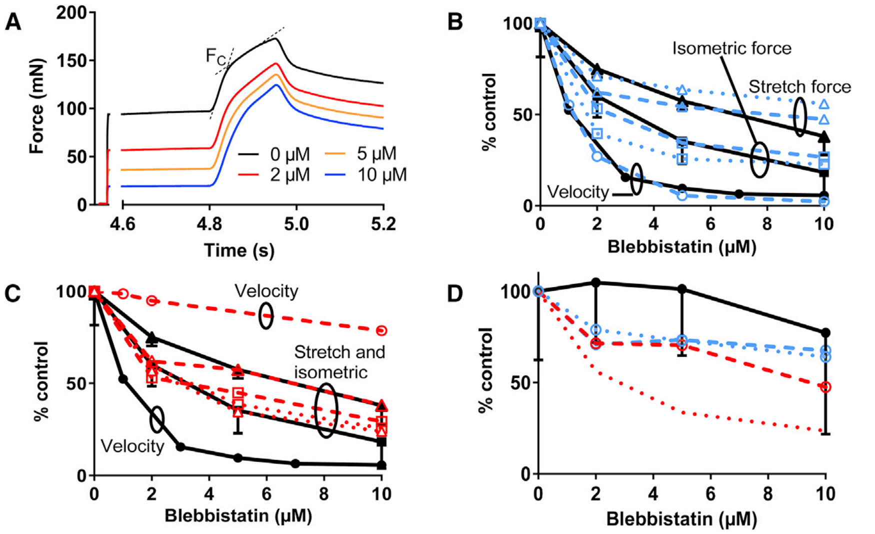

FIGURE 3 Effects of different concentrations of blebbistatin on contractile parameters in experiments and simulations. (*A*) Force records show the effects of blebbistatin on isometric force and the force response to stretch of fast skinned muscle fibers of rabbit psoas muscle. Stretch ramps at velocity of 2.5 fiber lengths/s (2500 nm/half-sarcomere/s) were imposed between times of 4.8 and 4.95 s during otherwise steady-state isometric contraction. The records were obtained in the presence of 0–10 μM blebbistatin. The critical force during stretch, F_c, was estimated as indicated by crossing dashed lines. (*B*) Concentration-effect curves for experiments (*filled symbols, full black lines*) were compared to the model (*blue*) assuming blebbistatin-induced reduction of rate constant (k_{P+}') for the transition from the AM*'DP to the AM*DP state from 1000 to 1.5 s^{-1}. Parameter values are either assumed to apply at 25–30°C (Tables S1 and S2, *open symbols, dashed lines*) or 5°C (Table S3, *dotted lines*). (*C*) Concentration-effect curves for experiments (*filled symbols, full lines*) were compared to the model assuming reduction of ΔG_{on} (Eq. S2) from 0.7 to −4 $k_B T$ (*red*). Other parameter values for 25–30°C (Tables S1 and S2, *open symbols, dashed lines*) or 5°C (Table S3, *dotted lines*) are shown. (*D*) Force enhancement during stretch (Fc-isometric force) is plotted versus blebbistatin concentration with color and symbol coding as in (*B*) and (*C*), i.e., lower data sets (*red*), model assuming reduction in ΔG_{on}, and middle data sets (*blue*), model assuming reduction in k_{P+}'. All data are normalized to control values. The experimental data for sliding velocity (*circles*) are from Fig. 1 *A* (blebbistatin batch with highest activity). Error bars, 95% confidence interval. $N = 16$ muscle fibers are tested for each blebbistatin concentration. Blebbistatin affinity (1 μM^{-1}) in the simulations is shown according to measurements in (41) using rabbit fast muscle myosin. For absolute values of forces and velocity in simulations, see Figs. 2, 4, and S10 (high temperature).

These data show that the relative magnitude of the blebbistatin-induced reduction varies in the following order: effect on velocity > effect on isometric force > effect on F_c. The experimental data in Fig. 3 are shown together with simulation data referring to different conditions (see further below). The experimental results in Fig. 3, *A* and *D* show that the force enhancement during stretch was little changed by blebbistatin, more consistent with lower blebbistatin-induced reduction of the critical force during stretch than of the isometric force (Fig. 3, *B* and *C*). This result fits with the idea that actomyosin cross-bridges with blebbistatin contribute to the force response during stretch in our experiments rather than being parked on the thick filament backbone. This can be inferred by comparing to the effects of varied Ca^{2+} concentrations in which the force during stretch was approximately proportional to the isometric force (51). The results are consistent with a proportionally larger reduction in isometric force by blebbistatin than in the number of attached cross-bridges resisting lengthening.

Model predictions of experimental results

An ideal model for actomyosin-based force generation should be as simple as possible while accounting for contractile phenomena both under normal physiological conditions and in the presence of mutations and/or modifying chemical compounds. A suitable model for initial analysis, as motivated in the Discussion, is that in (29) (Fig. S8). However, this model (29) could not predict the very substantial inhibiting effect of blebbistatin on actomyosin ATPase while simultaneously accounting for similar reduction in sliding velocity for short and long actin filaments in the in vitro motility assay. The blebbistatin-induced reduction of the ATPase activity in the model could only be achieved by lowering the rate constant for the transition between weakly and strongly bound actomyosin cross-bridge states ($k_{on}(x)$ in Fig. S8 *A*). However, this mechanism appreciably lowered the sliding velocity only for short and not for long actin filaments. The alternative mechanism, which shows that blebbistatin acts primarily by inhibiting the power stroke ("the Huxley and Simmons transition" (30) governed by K_{HL} in Fig. S8 *A*), does not explain the blebbistatin effect on the actomyosin ATPase. Neither the combination of the two mechanisms is successful in accounting for the blebbistatin effects (Fig. S8, *B* and *C*). Thus, in contrast to the experimental findings, an appreciable filament length dependence of the blebbistatin effect on velocity was predicted, and the K_{ATPase} for the actin-activated ATPase activity would be increased by blebbistatin (Fig. S8 *B*) rather than decreased, as found experimentally (41). This suggests that a more complex model is required.

The model in Fig. S8 (29) was therefore expanded (Figs. S9 and 4) by incorporating recent ideas for the mechanism of phosphate release (19). More specifically, the ideas of Llinas et al. (19) were integrated into the model (29) by addition (Fig. 4, *A* and *B*; schematic in Fig. 1 *C*) of 1) a weakly but stereospecifically bound pre-power-stroke state (AM*'DP) and 2) a phosphate release state (AM*DP) (Fig. 4 *A*). These states precede the main force-generating transition ((30); equilibrium constant K_{LH}) that is

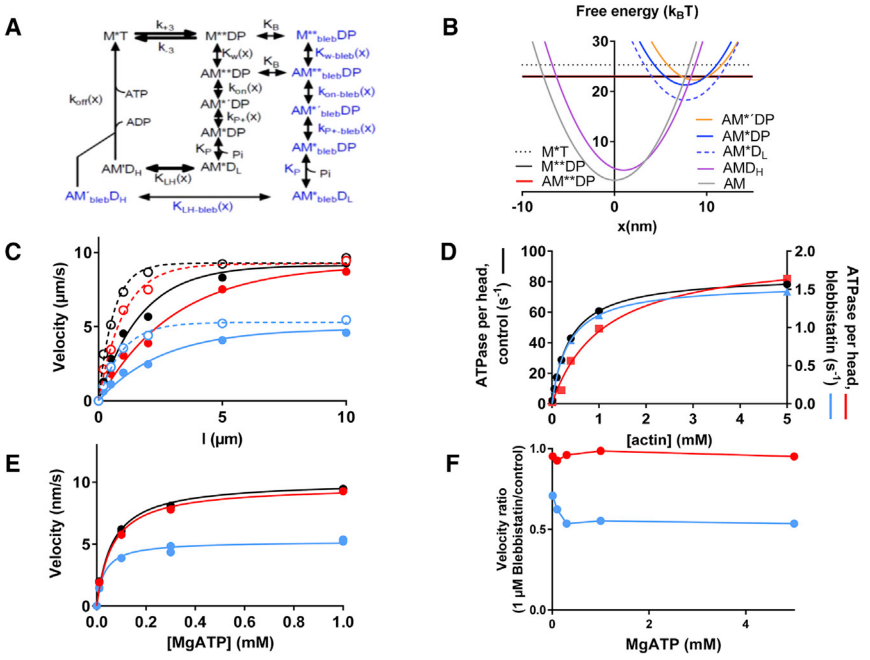

FIGURE 4 Modeling of blebbistatin effects. (*A*) Kinetic scheme of model states shows myosin (M) free or bound to actin (*A*) with ATP (T), ADP (D), and/or inorganic phosphate (P, Pi) at the active site. Several states exist also in blebbistatin-bound form (*blue*; subscript "bleb"). Rate constants or rate functions are indicated by the generic form $k_i(x)$ and equilibrium constant by K_i. Only forward rates are indicated. (*B*) Free-energy diagrams illustrate the dependence on the variable *x* of the free energy of cross-bridges in the states without blebbistatin. Here, *x* is the distance between the nearest actin site and a reference point on the myosin filament with x = 0 nm at the free-energy minimum of the AM state. (*C*) Simulated velocity length plots in the in vitro motility assay with standard conditions (*black*) and 1 μM blebbistatin (*colored circles*) are shown, assuming changes in parameter values corresponding to different hypotheses for blebbistatin effects. Simulations for 1 μM blebbistatin assumed either one (*filled symbols* and *full lines*) or three (*open symbols* and *dashed lines*) myosin-binding sites for actin per 36 nm half-repeat of the actin filament. Conditions tested included the following: 1) ΔG_{on} reduced from 0.7 to -4 k_BT (*red*; reduction in $k_{on}(x)$; Eq. S2) and 2) k_{P+}' reduced from 1000 to 1.5 s^{-1} (*blue*; Eqs. S4 and S5). (*D*) Simulated relationships are shown between actomyosin ATPase rate and actin concentration in solution. Color coding corresponds to that in (*C*), but a saturating blebbistatin concentration is assumed when the simulations are performed with the compound believed to be present. Curves represent fits of hyperbolic functions to the data with Michaelis-Menten constants $V_{max} = 84.2 \pm 0.2$ s^{-1} (mean $\pm$ 95% confidence interval) and $K_{ATPase} = 0.386 \pm 0.001$ mM under control conditions (*black*; *left axis*) and $V_{max} = 1.98 \pm 0.20$ s^{-1} and $K_{ATPase} = 1.09 \pm 0.33$ mM (*red*; *right axis*) or $V_{Max} = 1.57 \pm 0.02$ s^{-1} and $K_{ATPase} = 0.355 \pm 0.002$ mM (*blue*; *right axis*). (*E*) Sliding velocity vs. [MgATP] is simulated using parameter values according to color coding in (*C*) and (*D*). Curves represent fits of hyperbolic functions to the data (including points at 5 mM MgATP; data not shown) with Michaelis-Menten constants $V_{Max} = 10.1 \pm 0.4$ μm/s and $K_M^v = 0.062 \pm 0.015$ mM under control conditions (*black*) and $V_{Max} = 5.22 \pm 0.34$ μm/s and $K_M^v = 0.031 \pm 0.010$ mM assuming reduced k_{P+}' (*blue*) or $k_{on}(x)$ (*red*) in the presence of 1 μM blebbistatin. Filaments are assumed to be 20 μm long. Note the slightly low V_{Max} and the low K_M^v values compared to the experimental data in Fig. S7 would be increased by assuming nonlinear cross-bridge elasticity (55), but this was not further pursued here. (*F*) Simulated sliding velocity vs. [MgATP] is shown in the presence of 1 μM blebbistatin as a fraction of velocity in the absence of the blebbistatin using parameter values according to color coding in other panels.

responsible for the fast force recovery in response to length steps. The expanded model, appropriately reduced as described and motivated in Fig. S9, maintains the capacity of the original model (29) to account for several experimental findings in the absence of blebbistatin. This includes the shape of the force-velocity relationship and the relationships between [Pi] on the one hand and the magnitudes of the maximal sliding velocity and the maximal isometric force on the other (Fig. S1, *A* and *B*). Furthermore, the model is consistent with recent data ((18); see Fig. S1, *C* and *D*) suggesting that both phosphate release and cross-bridge attachment into force-generating states are rate-limited by the same transition(s) ($k_{on}(x)$; Fig. 4 *A*). Also, the actomyosin ATPase is primarily rate limited by $k_{on}(x)$ in the model, but it is modulated (Legend, Fig. S1) by the rate constant k_{+3} (Fig. 4 *A*). Here, $k_{on}(x)$ is the rate function (denoted attachment rate constant in the following discussion) for the transition from the nonstereospecific weakly bound actomyosin state AM**DP to the stereospecific weakly bound state AM*'DP.

The blebbistatin binding to and dissociation from the myosin head is treated in a simplified way as rapid equilibrium binding ($K_{bind} = 1$ μM^{-1}) to the M**DP state and irreversible dissociation from the AM'D_H state. These simplifications account well for the blebbistatin effects on studied mechanical parameters (see below) and also accord with more than an order of magnitude increase (41) in the effective equilibrium between the M**DP states (the M**DP + M**DP_{bleb} states) and the M*T state. The effective equilibrium constant increases from ~13 to ~995 without any change in K_3 because of the greatly increased population of the M**DP_{bleb} state upon addition of blebbistatin.

We next tested the hypothesis that the blebbistatin effects are explained by reduced value of $k_{on}(x)$ in the expanded scheme in Fig. 4 *A*. A reduced magnitude of this rate function is sufficient to account for the blebbistatin-induced reduction of V_{max} of the actomyosin ATPase (41,43). Additionally, it correctly predicts reduced isometric force and reduction in velocity for 1-μm-long filaments. However, the predicted velocity-reduction for the longest filaments (5–20 μm) studied in the model is appreciably smaller than that observed experimentally (Figs. 2 *C* and 4 *C*). Importantly, this conclusion is further strengthened under the assumption of three binding sites for myosin per helical half-repeat of actin (Fig. 2 *C*). Furthermore, the K_{ATPase} ([actin] for half-maximal steady-state ATPase activity) is increased (Fig. 4 *D*) in

contrast to the small decrease found experimentally (41). These findings, together with proportionally rather similar blebbistatin-induced reduction in isometric force and force enhancement during stretch in simulations (Fig. 3 *D*; Fig. S10 *B*), argue strongly against the idea that blebbistatin acts by lowering $k_{on}(x)$. Indeed, the finding that a reduction of $k_{on}(x)$ alone cannot account for the blebbistatin effect is entirely consistent with the findings based on the simpler model (29) (Fig. S8). This follows because the latter model is virtually identical to the expanded model (Fig. 4 *A*) unless the rate function $k_{P+}(x)$ is appreciably reduced below its standard value (1000 s^{-1}). The latter high value was chosen based on control simulations suggesting that $k_{P+}(x)$ must be higher than 500 s^{-1} in the absence of blebbistatin to explain the high velocity (~10 μm/s) under these conditions. Thus, under physiological conditions, the AM*'DP state would be negligibly populated, and its existence is revealed by functional studies only after adding inhibiting compounds such as blebbistatin.

Alternative hypotheses that were initially considered include the effects of blebbistatin on the phosphate affinity (Kp) or the main force-generating transition (30) between the AM*D_L and the AM'D_H state. However, these ideas are readily falsified without further analysis because neither of them accounts for the hallmark finding that blebbistatin appreciably reduces V_{max} of the actomyosin ATPase. It may, however, be worth mentioning that isolated inhibition of the AM*D_L-AM'D_H transition in the model would markedly reduce both the sliding velocity and the maximal force, whereas altered Kp has no effect on sliding velocity.

Finally, we tested the hypothesis that blebbistatin slows the transition from the AM*'DP to the AM*DP state (reduction in $k_{P+}(x)$; Fig. 4 *A*), i.e., the transition from a stereospecifically attached pre-power-stroke state to the phosphate release state (19). To quantitatively account for the reduced V_{max} of the actomyosin ATPase, the transition rate has to be reduced 650-fold. Good fits are obtained whether both the backward and forward reactions are reduced equally (i.e., increased activation energies for these transitions) or if only the forward rate constant is slowed by blebbistatin. The latter change would be consistent with higher free energy of the states after the AM*'DP state. Importantly, the magnitude of the reduction in $k_{P+}(x)$, necessary to account for the blebbistatin-induced reduction in the maximal ATPase activity, quantitatively predicts other experimental findings by us and others without the need for further assumptions. The findings that are accounted for include 1) reduction in the maximal sliding velocity (Fig. 4 *C*), 2) reduction in the maximal isometric force (Fig. 3 *B*), 3) reduction in the K_{ATPase} (Fig. 4 *D*), 4) reduction of K_M^v (Fig. 4 *E*), and 5) the lack of noticeable blebbistatin-induced change in the shape of the velocity-length plot (Fig. 4 *C*).

The favored mechanism (Fig. 3, *B* and *D*), compared to the mechanism assuming reduced attachment rate constant ($k_{on}(x)$ (Fig. 3, *C* and *D*), also provides quantitatively better reproduction of the blebbistatin-induced effects on force during stretch of active muscle (Fig. 3 *A*). This also applied if we modified the parameter values (Table S3) to be more consistent with the low temperature (5°C) of the experiment (Fig. 3, *B–D*). Finally, a shift of the rate-limiting transition to that between the AM*'DP to the AM*DP state ($k_{P+}(x)$) would reduce the rate of rise of isometric force. However, the predicted reduction in this rate is appreciably lower (~5-fold; Fig. S10) than the effect on the V_{max} of the actomyosin ATPase (~50-fold reduction). We tested whether the model predictions may be further improved by introducing blebbistatin-induced inhibition of the power stroke (44). Adding this mechanism to reduced value of k_{P+} in response to blebbistatin improves the fit to the stretch response (Fig. S10 *D*) and to some other experimental results (Table S4).

DISCUSSION

Our central result is that a mechanokinetic model incorporating recent ideas for the phosphate release and the force-generating process (18,19,25,29) accounts for a substantial set of experimental data both in the presence (Figs. 1, 2, and 3) and absence (Figs. 1, 2, 3, S1, and S2) of blebbistatin. The final model (Fig. 4 *A*) was developed by expanding a recent simpler model (29). The latter model (29), in turn, was selected for initial tests because all states are independently supported by biochemical, physiological, or structural studies (19,29), allowing direct connections between biochemistry, structure, and contractile function. This model is defined by parameter values obtained under coherent conditions with regard to temperature, ionic strength, muscle type, etc. ((29); Tables S1 and S2). Furthermore, the elastic properties and free-energy profiles are consistent with single-molecule mechanics (72,73) but have been fine-tuned to fit force-velocity data (29,33,55,74) of fast vertebrate skeletal muscle. Finally, the model (29) accounts for findings ranging from single-molecule experiments over experiments on small myosin ensembles to the large ordered ensembles in muscle cells.

With regard to the sequence of events defining the initially tested model in Fig. S8 *A* (29), the rate-limiting steps, the two-step Pi release, and the temporal relationship between these events as well as the main force-generating structural change all have independent support in the literature. First, there is evidence that the same transition limits actomyosin ATPase activity and force generation ((28); see also (29)). In addition, recent experimental results on cardiac myofibrils suggest that the same transition is rate limiting for the tension changes in response to jumps in phosphate and calcium concentrations as well as in response to slackening-restretch procedures (18). This suggests that both force generation from the detached state and force changes in response to jumps in phosphate concentration are rate limited by the transition ($k_{on}(x)$) that also rate-limits actomyosin ATPase. Admittedly, direct evidence for

identical rate limitation for Pi transients and for force generation from the relaxed state has not been provided for fast rabbit skeletal muscle. However, the sarcomere nonuniformities that have central roles in the studies of cardiac myofibrils by Stehle (18) bear strong resemblance to those underlying the two phases of relaxation after a tetanus of fast skeletal muscle fibers (75). This demonstrates the existence of closely related mechanisms for tension relaxation in both preparations. Furthermore, two phases of tension relaxation have also been observed in skeletal muscle fibers upon sudden increase in Pi concentration but without the possibility for detailed analysis of the first phase due to its brief duration (9). These findings, taken together, provide arguments suggesting that the recent observations in cardiac myofibrils (18) can be extrapolated to skeletal muscle fibers. Finally, an identical rate-limiting step for Pi transients and force generation is consistent with the structural data (19,20).

A second important feature of the model in Fig. S8 *A* (29) is that Pi release is a two-step process with one slow isomerization in series with rapid equilibrium Pi release (9,12,17). In two recent models (25,29), the slow step has been assumed to correspond to the transition between the weak-binding and first strong-binding actomyosin state just before Pi release. In this connection, we agree with arguments put forward by Smith (25) against a slow Pi release step due to the severe velocity reduction it would produce. We also agree with arguments (25) based on energetics and effects of [Pi] on tension, suggesting that Pi release occurs before the force-generating structural change. The emerging picture with slow cross-bridge attachment, followed by fast Pi-release and the force-generating structural transition in sequence, is also fully consistent with structural findings (19,20). One of the latter studies ((19); see further (25)) also provides evidence for an intermediate phosphate-binding site in the Pi-release tunnel that causes a delay before the released phosphate appears in solution. This can account for recent results from myosin II (15) and myosin V (26), which have suggested that Pi release occurs after the force-generating structural state. The fast force generation (K_{HL}) after the Pi-release in the model (29) corresponds directly to that suggested by Huxley and Simmons (30) to be responsible for the rate of the fast force recovery after length steps. This idea fits with the strain dependence of the transition governed by K_{LH} in the model. The strain dependence follows from a shift along the *x* axis of the free-energy diagrams for the AM*D_L and AM'D_H states relative to each other (Fig. 4 *B*). The proposed sequence of events is also consistent with the lack of [Pi] effect on the tension recovery process after a shortening step. The situation for stretches is more complex. A rapid stretch would thus increasingly populate the AM*D_L state at x-values close to its free-energy minimum and close to the x-value at which transitions back and forth between the AM*D_L state and the M**DP state are most likely.

We take the above arguments as solid foundations for using the model (29) as a starting point for evaluating the blebbistastin effects. However, as shown above, neither that model nor, for example, that in (25), could account for the data with blebbistatin unless we also added states and transitions similar to those proposed by Llinas et al. (19). After the latter modification, the following view emerges: the initial transition from weakly to strongly bound pre-power-stroke states is rate limiting under normal conditions, but blebbistatin shifts the rate-limiting step to the subsequent transition between a pre-power-stroke state and a Pi release state (19). Thus, blebbistatin gives functional significance to the otherwise transient pre-power-stroke state proposed in (19). In the Pi release state, there is rapid equilibrium Pi release from the active site without any change in position of the lever arm. This is consistent with the same x-values for the free-energy minima of the AM*DP and the AM*D_L states. After phosphate release, a rapid force-generating structural change with a swing of the lever arm (the "power stroke" (30)) follows (between the AM*D_L and the AM'D_H states), as indicated schematically by model 2 in Fig. 1 *C*.

Some issues regarding the quantitative analysis of the two major hypotheses (effect on $k_{P+}(x)$ or $k_{on}(x)$) deserve comments. First, to summarize the principle of the analysis, we do not explore the entire parameter space, but instead, we first fix all parameters at literature values to simulate results in the absence of blebbistatin. Then, the magnitude of the blebbistatin effect on $k_{P+}(x)$ or $k_{on}(x)$ is inferred from the effect of saturating blebbistatin concentration on the actin-activated ATPase activity. Next, we performed Monte Carlo simulations to test if either of these changes also account for other blebbistatin effects observed by us and others.

This analysis, using fixed parameter values, has both advantages and disadvantages compared to methods relying on nonlinear regression, e.g., downhill simplex minimization procedures (76) in which the entire parameter space is explored. Of course, nonlinear regression would not be practically feasible in this case using slow Monte Carlo simulations. However, assuming that such an approach would be possible, it would nevertheless be problematic. Thus, with this number of parameter values, multiple local minima may occur in the error function with uncertainties in the best fit values. On the other hand, one may argue that fixing the parameter space may also introduce errors, e.g., if one or several of the fixed parameter values are erroneous, a worst-case scenario may be that the best fit (in the least-square sense) is obtained for a false hypothesis. However, in this case, this would seem to require appreciable uncertainties in the parameter values because the model assuming reduced $k_{P+}(x)$ gives a sum of squared errors that is more than one order of magnitude lower (Table S4) than that of the model assuming reduced $k_{on}(x)$. Also, importantly, the differences in the sum of squared errors between the two major models (Table S4) originate in substantial differences

in the predictions of several individual variables (Fig. 4). Furthermore, the idea of appreciable uncertainties in the parameter values is in conflict with the very good fits to a range of independent and complex experimental data using the parameter values in Tables S1 and S2. This includes the force-velocity relationship and the actomyosin ATPase without blebbistatin (Figs. S1 and 4 in main article). Some of the parameter values, such as those defining the free-energy diagrams (cross-bridge stiffness, level, and *x*-position of free-energy minima) are also linked in the sense that change of one require changes of others to give reasonable force-velocity relationships. This argues against independent searches of the entire parameter space. Additionally, the existence of linked parameter values contributes to greater certainty in the values that we have actually used. Thus, although the latter define the free-energy profiles in a way that is consistent with recent optical tweezers data (72,73), they have also been fine-tuned to each other (including subnanometer and single k_BT changes in position and levels of the free-energy minima) in several studies (29,33,55,74) to account for the force-velocity relationship. The above arguments suggest minimal risks of error in selecting between the two major models for the blebbistatin effects due to uncertainties in the parameter values used (Tables S1 and S2). This view is supported by the analysis in Fig. S11 and Table S5. Here, we tested the model predictions for the two major hypotheses above using different sets of parameter values, in which each value was randomly selected to be either 25% higher or 25% lower than the literature value in Tables S1 and S2. First, it is of interest to note that only 2 out of 10 sets of alternative parameter values selected in this way gave reasonable fits to the experimental force-velocity data. However, importantly, for all of six tested sets of parameter values (including those fitting the force-velocity relationship), the blebbistatin results were more faithfully reproduced by a reduction in kp+' than by a reduction in $k_{on}(x)$.

Both the AM*'DP and the AM*DP states are essential in the model in Fig. 4, *A* and *B* to account for the blebbistatin effects and most likely for the effects of other drugs such as 2,3-butanedione 2-monoxime (BDM) and N-benzyl-p-toluene sulphonamide (BTS) (35,36). Additional challenging tests would ascertain whether the model, in detail, predicts effects of altered temperature (77), effects of other drugs (6,38), and various mutation effects. It should also be tested if the model can account for phenomena previously found to require states and transitions without independent evidence in the literature (14,27).

An important basis for interpreting the effects of blebbistatin on force, velocity, and actomyosin ATPase within one theoretical framework and one mechanism was the finding that the blebbistatin effects on velocity in the in vitro motility assay (in contrast to the situation in cells (52)) do not require phosphorylation of the RLCs. The results support the view that the lack of blebbistatin effects in the absence of RLC phosphorylation in cells is due to blebbistatin-induced enhancement of myosin head interactions with the thick filament backbones (absent in the in vitro motility assay). These interactions prevent the myosin heads with bound blebbistatin to interact with actin. When such interactions are eliminated, however, as in these in vitro motility assays, it is reasonable to view the reduction in isometric force, actomyosin ATPase rate, and sliding velocity as different facets of one specific blebbistatin effect on actomyosin function.

A remaining uncertainty (29) in the expanded model (Fig. 4) is whether the cross-bridge elasticity is linear or nonlinear (55,72). Our assumption of linear, rather than nonlinear, cross-bridge elasticity could explain (29,55) the lower K_M^v value (for sliding velocity vs. [MgATP]) in simulations; Fig. 4 *E*) than that in experiments (Fig. 2 *D*). Also, the exact mechanism underlying the response of active muscle to stretch is uncertain (78,79). Therefore, this treatment in this regard is necessarily somewhat tentative.

One source of complexity compared to previous models (41) is that the blebbistatin-inhibited step is different from the step that normally rate-limits the actomyosin ATP turnover cycle. Additionally, this transition occurs between two stereospecifically attached states. This means that when blebbistatin is bound to myosin, the transition between these states (governed by $k_{P+}(x)$; very fast in the absence of blebbistatin) is so extensively inhibited that it becomes the new rate limiting step for the cycle instead of $k_{on}(x)$. It is important to note in this connection that we do not propose an alternative pathway for Pi release upon blebbistatin binding. Instead, we suggest that the rate of a specific transition in the normal path (19,25,29) is appreciably reduced.

The idea that blebbistatin shifts the rate-limiting step to a transition between attached states implies increased fraction of actin-bound myosin heads in the presence of MgATP. This idea is consistent with the blebbistatin-induced reduction of the K_{ATPase} for the actin-activated ATPase and with the finding that blebbistatin-induced complete motility inhibition in the in vitro motility assay did not cause filament detachment from the myosin-coated surface (80). In contrast, no clearly noticeable blebbistatin-induced change in myosin head binding to actin in the presence of μM MgATP could be deduced from pyrene-actin fluorescence or light-scattering data. This led Kovacs et al. (41) to conclude that also in the presence of blebbistatin, the myosin heads are predominantly not strongly bound to actin during steady-state ATP hydrolysis. Besides, a 50% blebbistatin-induced reduction in myosin binding to actin was observed in a cosedimentation assay in the presence of 10 mM MgATP, ~1 mM free Mg^{2+}, and 0 mM monovalent salt (41). These findings imply complexities in the experimental results (e.g., effects of low ionic strength on blebbistatin effects on actin-binding) or possibly in the mechanism of action of blebbistatin. These complexities deserve further study. For instance, it would be of interest to investigate whether blebbistatin stabilized state(s) could be detected

in single-molecule studies such as ultrafast optical tweezers based force spectroscopy (81). In this connection, it is of interest to note that one of the pre-power-stroke cross-bridge states found by Capitanio et al. (81) using fast myosin II has properties reminiscent of the AM*'DP state suggested to be increasingly populated due to the blebbistatin effect.

Our model agrees with the generally held view that blebbistatin appreciably reduces the population of strongly bound (Actin-Mysoin and Actin-Myosin-Adenosine diphosphate (AMD)) states. However, we found no noticeable change in the shape of the velocity length plot. This suggests that the blebbistatin effects cannot be unambiguously described using the duty ratio concept (64,82). Thus, whereas the duty ratio is defined as the fraction of the cycle time spent by the myosin heads in strongly bound force-producing (AM and AMD) states (64,82), its numerical value is generally estimated from the shape of the plot of velocity versus the number of available myosin heads (29,39,64,83). Such plots in our study suggest virtually unchanged duty ratios in the presence of blebbistatin, whereas all other experimental data point to fewer heads in the AM and AMD states, consistent with the reduced duty ratio.

The need to assume a changed rate-limiting step of the actomyosin ATPase to account for the simultaneous blebbistatin effects on ATPase and sliding velocity is consistent with evidence that these two variables are governed by different transitions in the absence of blebbistatin. Thus, in vitro sliding velocity and the unloaded shortening velocity in muscle on the one hand and V_{max} of the actomyosin ATPase on the other exhibit different temperature dependence (60) for several different myosin isoforms (61). Additionally, a genetic modification of the myosin motor that reduced the actomyosin ATPase activity several-fold had no effect on the in vitro sliding velocity (84). Accordingly, our findings are consistent with the detachment limitation (29,60,61,85) rather than attachment limitation (86) of shortening velocity in muscle fibers under physiological conditions as well as of the actin filament sliding velocity driven by a large number of myosin motors in the in vitro motility assay. On the other hand, as mentioned above, the rate of isometric force development and V_{max} of the actomyosin ATPase are primarily rate-limited by the attachment rate, $k_{on}(x)$, under physiological conditions. Additionally, increased attachment rate will increase the sliding velocity when the number of available myosin motors is considerably fewer (87) than that operating together in the muscle cell.

To conclude, this study has unveiled otherwise hidden secrets in the actomyosin cross-bridge cycle from attachment of myosin to actin over Pi release to force generation. Additionally, the model accounts for a wide range of contractile phenomena with the potential to clarify the currently quite bewildering picture of the analyzed phase of the cycle. We expect the model to be valuable in drug discovery efforts aiming to fine-tune specific transition rates in the force-generating cycle, either for adapting the function of normal myosin to abnormal conditions (e.g., altered load, surrounding areas with dead myocardium) or for correcting myosin malfunction, e.g., due to mutations or posttranslational modifications. Whereas blebbistatin is selective for myosin II, the model is likely to be generally valid for any myosin after appropriate modification of the numerical values of different rate functions. This is suggested by evidence (19) that critical states and transitions in this model (Fig. 4 *A*) are of relevance in several myosin classes.

SUPPORTING MATERIAL

Supporting Materials and Methods, eleven figures, and five tables are available at http://www.biophysj.org/biophysj/supplemental/S0006-3495(18)30682-9.

AUTHOR CONTRIBUTIONS

A.M. conceived the study, developed the model, ran model simulations, and analyzed model data in relation to experiments. M.A.R. performed most experiments on isolated proteins and analyzed the data. M.U. performed some of the experiments on isolated proteins and analyzed the data. D.E.R. performed experiments on muscle fibers and analyzed the data. A.M. coordinated the project, but all authors contributed to planning the work, to data interpretation, and to writing the article.

ACKNOWLEDGMENTS

This work was funded by European Union Seventh Framework Future and Emerging Technologies Programme under contract 613044 (ABACUS) and Horizon2020 Future and Emerging Technologies Programme under contract 732482 (Bio4comp). Further, funding is acknowledged from The Swedish Research Council (grant number 2015-05290), The Faculty of Health and Life Sciences at The Linnaeus University, and the Natural Science and Engineering Research Council of Canada. D.E.R. is a Canada Research Chair (Tier I) in Muscle Biophysics.

REFERENCES

1. Heissler, S. M., and D. J. Manstein. 2013. Nonmuscle myosin-2: mix and match. *Cell. Mol. Life Sci.* 70:1–21.
2. Spudich, J. A. 2014. Hypertrophic and dilated cardiomyopathy: four decades of basic research on muscle lead to potential therapeutic approaches to these devastating genetic diseases. *Biophys. J.* 106:1236–1249.
3. Ochala, J., and Y. B. Sun. 2016. Novel myosin-based therapies for congenital cardiac and skeletal myopathies. *J. Med. Genet.* 53:651–654.
4. Tajsharghi, H., and A. Oldfors. 2013. Myosinopathies: pathology and mechanisms. *Acta Neuropathol.* 125:3–18.
5. Li, M., H. Ogilvie, ..., L. Larsson. 2015. Aberrant post-translational modifications compromise human myosin motor function in old age. *Aging Cell.* 14:228–235.
6. Malik, F. I., J. J. Hartman, ..., D. J. Morgans. 2011. Cardiac myosin activation: a potential therapeutic approach for systolic heart failure. *Science.* 331:1439–1443.
7. Green, E. M., H. Wakimoto, ..., C. E. Seidman. 2016. A small-molecule inhibitor of sarcomere contractility suppresses hypertrophic cardiomyopathy in mice. *Science.* 351:617–621.

8. Tang, W., C. A. Blair, …, C. M. Yengo. 2017. Modulating beta-cardiac myosin function at the molecular and tissue levels. *Front. Physiol.* 7:659.
9. Dantzig, J. A., Y. E. Goldman, …, E. Homsher. 1992. Reversal of the cross-bridge force-generating transition by photogeneration of phosphate in rabbit psoas muscle fibres. *J. Physiol.* 451:247–278.
10. Kawai, M., and H. R. Halvorson. 1991. Two step mechanism of phosphate release and the mechanism of force generation in chemically skinned fibers of rabbit psoas muscle. *Biophys. J.* 59:329–342.
11. Davis, J. S., and N. D. Epstein. 2009. Mechanistic role of movement and strain sensitivity in muscle contraction. *Proc. Natl. Acad. Sci. USA.* 106:6140–6145.
12. Ranatunga, K. W. 2010. Force and power generating mechanism(s) in active muscle as revealed from temperature perturbation studies. *J. Physiol.* 588:3657–3670.
13. Smith, D. A., and J. Sleep. 2004. Mechanokinetics of rapid tension recovery in muscle: the Myosin working stroke is followed by a slower release of phosphate. *Biophys. J.* 87:442–456.
14. Caremani, M., L. Melli, …, M. Linari. 2013. The working stroke of the myosin II motor in muscle is not tightly coupled to release of orthophosphate from its active site. *J. Physiol.* 591:5187–5205.
15. Muretta, J. M., K. J. Petersen, and D. D. Thomas. 2013. Direct real-time detection of the actin-activated power stroke within the myosin catalytic domain. *Proc. Natl. Acad. Sci. USA.* 110:7211–7216.
16. Lionne, C., M. Brune, …, T. Barman. 1995. Time resolved measurements show that phosphate release is the rate limiting step on myofibrillar ATPases. *FEBS Lett.* 364:59–62.
17. Tesi, C., F. Colomo, …, C. Poggesi. 2000. The effect of inorganic phosphate on force generation in single myofibrils from rabbit skeletal muscle. *Biophys. J.* 78:3081–3092.
18. Stehle, R. 2017. Force responses and sarcomere dynamics of cardiac myofibrils induced by rapid changes in [P_i]. *Biophys. J.* 112:356–367.
19. Llinas, P., T. Isabet, …, A. Houdusse. 2015. How actin initiates the motor activity of Myosin. *Dev. Cell.* 33:401–412.
20. Reubold, T. F., S. Eschenburg, …, D. J. Manstein. 2003. A structural model for actin-induced nucleotide release in myosin. *Nat. Struct. Biol.* 10:826–830.
21. Pate, E., and R. Cooke. 1989. A model of crossbridge action: the effects of ATP, ADP and Pi. *J. Muscle Res. Cell Motil.* 10:181–196.
22. Månsson, A., D. Rassier, and G. Tsiavaliaris. 2015. Poorly understood aspects of striated muscle contraction. *BioMed Res. Int.* 2015:245154.
23. Stehle, R., and C. Tesi. 2017. Kinetic coupling of phosphate release, force generation and rate-limiting steps in the cross-bridge cycle. *J. Muscle Res. Cell Motil.* 38:275–289.
24. Houdusse, A., and H. L. Sweeney. 2016. How myosin generates force on actin filaments. *Trends Biochem. Sci.* 41:989–997.
25. Smith, D. A. 2014. A new mechanokinetic model for muscle contraction, where force and movement are triggered by phosphate release. *J. Muscle Res. Cell Motil.* 35:295–306.
26. Trivedi, D. V., J. M. Muretta, …, C. M. Yengo. 2015. Direct measurements of the coordination of lever arm swing and the catalytic cycle in myosin V. *Proc. Natl. Acad. Sci. USA.* 112:14593–14598.
27. Debold, E. P., S. Walcott, …, M. A. Turner. 2013. Direct observation of phosphate inhibiting the force-generating capacity of a miniensemble of myosin molecules. *Biophys. J.* 105:2374–2384.
28. Brenner, B., and E. Eisenberg. 1986. Rate of force generation in muscle: correlation with actomyosin ATPase activity in solution. *Proc. Natl. Acad. Sci. USA.* 83:3542–3546.
29. Månsson, A. 2016. Actomyosin based contraction: one mechanokinetic model from single molecules to muscle? *J. Muscle Res. Cell Motil.* 37:181–194.
30. Huxley, A. F., and R. M. Simmons. 1971. Proposed mechanism of force generation in striated muscle. *Nature.* 233:533–538.
31. Ranatunga, K. W., M. E. Coupland, and G. Mutungi. 2002. An asymmetry in the phosphate dependence of tension transients induced by length perturbation in mammalian (rabbit psoas) muscle fibres. *J. Physiol.* 542:899–910.
32. Eisenberg, E., and L. E. Greene. 1980. The relation of muscle biochemistry to muscle physiology. *Annu. Rev. Physiol.* 42:293–309.
33. Albet-Torres, N., M. J. Bloemink, …, A. Månsson. 2009. Drug effect unveils inter-head cooperativity and strain-dependent ADP release in fast skeletal actomyosin. *J. Biol. Chem.* 284:22926–22937.
34. McKillop, D. F., N. S. Fortune, …, M. A. Geeves. 1994. The influence of 2,3-butanedione 2-monoxime (BDM) on the interaction between actin and myosin in solution and in skinned muscle fibres. *J. Muscle Res. Cell Motil.* 15:309–318.
35. Regnier, M., C. Morris, and E. Homsher. 1995. Regulation of the cross-bridge transition from a weakly to strongly bound state in skinned rabbit muscle fibers. *Am. J. Physiol.* 269:C1532–C1539.
36. Shaw, M. A., E. M. Ostap, and Y. E. Goldman. 2003. Mechanism of inhibition of skeletal muscle actomyosin by N-benzyl-p-toluenesulfonamide. *Biochemistry.* 42:6128–6135.
37. Radke, M. B., M. H. Taft, …, D. J. Manstein. 2014. Small molecule-mediated refolding and activation of myosin motor function. *eLife.* 3:e01603.
38. Rohde, J. A., D. D. Thomas, and J. M. Muretta. 2017. Heart failure drug changes the mechanoenzymology of the cardiac myosin powerstroke. *Proc. Natl. Acad. Sci. USA.* 114:E1796–E1804.
39. Swenson, A. M., W. Tang, …, C. M. Yengo. 2017. Omecamtiv mecarbil enhances the duty ratio of human β-cardiac Myosin resulting in increased calcium sensitivity and slowed force development in cardiac muscle. *J. Biol. Chem.* 292:3768–3778.
40. Straight, A. F., A. Cheung, …, T. J. Mitchison. 2003. Dissecting temporal and spatial control of cytokinesis with a myosin II inhibitor. *Science.* 299:1743–1747.
41. Kovács, M., J. Tóth, …, J. R. Sellers. 2004. Mechanism of blebbistatin inhibition of myosin II. *J. Biol. Chem.* 279:35557–35563.
42. Limouze, J., A. F. Straight, …, J. R. Sellers. 2004. Specificity of blebbistatin, an inhibitor of myosin II. *J. Muscle Res. Cell Motil.* 25:337–341.
43. Ramamurthy, B., C. M. Yengo, …, H. L. Sweeney. 2004. Kinetic mechanism of blebbistatin inhibition of nonmuscle myosin IIb. *Biochemistry.* 43:14832–14839.
44. Takács, B., N. Billington, …, M. Kovács. 2010. Myosin complexed with ADP and blebbistatin reversibly adopts a conformation resembling the start point of the working stroke. *Proc. Natl. Acad. Sci. USA.* 107:6799–6804.
45. Franks-Skiba, K., R. Lardelli, …, R. Cooke. 2007. Myosin light chain phosphorylation inhibits muscle fiber shortening velocity in the presence of vanadate. *Am. J. Physiol. Regul. Integr. Comp. Physiol.* 292:R1603–R1612.
46. Tesi, C., F. Colomo, …, C. Poggesi. 2002. Characterization of the cross-bridge force-generating step using inorganic phosphate and BDM in myofibrils from rabbit skeletal muscles. *J. Physiol.* 541:187–199.
47. Minozzo, F. C., L. Hilbert, and D. E. Rassier. 2012. Pre-power-stroke cross-bridges contribute to force transients during imposed shortening in isolated muscle fibers. *PLoS One.* 7:e29356.
48. Allingham, J. S., R. Smith, and I. Rayment. 2005. The structural basis of blebbistatin inhibition and specificity for myosin II. *Nat. Struct. Mol. Biol.* 12:378–379.
49. Várkuti, B. H., M. Képiró, …, A. Málnási-Csizmadia. 2016. A highly soluble, non-phototoxic, non-fluorescent blebbistatin derivative. *Sci. Rep.* 6:26141.
50. Képiró, M., B. H. Várkuti, …, A. Málnási-Csizmadia. 2012. Azido-blebbistatin, a photoreactive myosin inhibitor. *Proc. Natl. Acad. Sci. USA.* 109:9402–9407.
51. Minozzo, F. C., and D. E. Rassier. 2010. Effects of blebbistatin and Ca2+ concentration on force produced during stretch of skeletal muscle fibers. *Am. J. Physiol. Cell Physiol.* 299:C1127–C1135.

52. Stewart, M., K. Franks-Skiba, and R. Cooke. 2009. Myosin regulatory light chain phosphorylation inhibits shortening velocities of skeletal muscle fibers in the presence of the myosin inhibitor blebbistatin. *J. Muscle Res. Cell Motil.* 30:17–27.
53. Pardee, J. D., and J. A. Spudich. 1982. Purification of muscle actin. *Methods Cell Biol.* 24:271–289.
54. Kron, S. J., Y. Y. Toyoshima, …, J. A. Spudich. 1991. Assays for actin sliding movement over myosin-coated surfaces. *Methods Enzymol.* 196:399–416.
55. Persson, M., E. Bengtsson, …, A. Månsson. 2013. Nonlinear cross-bridge elasticity and post-power-stroke events in fast skeletal muscle actomyosin. *Biophys. J.* 105:1871–1881.
56. Greenberg, M. J., T. R. Mealy, …, J. R. Moore. 2009. The molecular effects of skeletal muscle myosin regulatory light chain phosphorylation. *Am. J. Physiol. Regul. Integr. Comp. Physiol.* 297:R265–R274.
57. Perrie, W. T., and S. V. Perry. 1970. An electrophoretic study of the low-molecular-weight components of myosin. *Biochem. J.* 119:31–38.
58. Gillespie, D. T. 1976. A general method for numerically simulating the stochastic time evolution of coupled chemical reactions. *J. Comput. Phys.* 22:403–434.
59. Huxley, A. F. 1957. Muscle structure and theories of contraction. *Prog. Biophys. Biophys. Chem.* 7:255–318.
60. Siemankowski, R. F., M. O. Wiseman, and H. D. White. 1985. ADP dissociation from actomyosin subfragment 1 is sufficiently slow to limit the unloaded shortening velocity in vertebrate muscle. *Proc. Natl. Acad. Sci. USA.* 82:658–662.
61. Yengo, C. M., Y. Takagi, and J. R. Sellers. 2012. Temperature dependent measurements reveal similarities between muscle and non-muscle myosin motility. *J. Muscle Res. Cell Motil.* 33:385–394.
62. Brenner, B., M. Schoenberg, …, E. Eisenberg. 1982. Evidence for cross-bridge attachment in relaxed muscle at low ionic strength. *Proc. Natl. Acad. Sci. USA.* 79:7288–7291.
63. Woledge, R. C., N. A. Curtin, and E. Homsher. 1985. Energetic Aspects of Muscle Contraction. Academic Press, London.
64. Uyeda, T. Q., S. J. Kron, and J. A. Spudich. 1990. Myosin step size. Estimation from slow sliding movement of actin over low densities of heavy meromyosin. *J. Mol. Biol.* 214:699–710.
65. Ferenczi, M. A., Y. E. Goldman, and R. M. Simmons. 1984. The dependence of force and shortening velocity on substrate concentration in skinned muscle fibres from Rana temporaria. *J. Physiol.* 350:519–543.
66. Xu, S., H. D. White, …, L. C. Yu. 2009. Stabilization of helical order in the thick filaments by blebbistatin: further evidence of coexisting multiple conformations of myosin. *Biophys. J.* 96:3673–3681.
67. Edman, K. A. 1999. The force bearing capacity of frog muscle fibres during stretch: its relation to sarcomere length and fibre width. *J. Physiol.* 519:515–526.
68. Getz, E. B., R. Cooke, and S. L. Lehman. 1998. Phase transition in force during ramp stretches of skeletal muscle. *Biophys. J.* 75:2971–2983.
69. Rassier, D. E. 2008. Pre-power stroke cross bridges contribute to force during stretch of skeletal muscle myofibrils. *Proc. Biol. Sci.* 275:2577–2586.
70. Månsson, A. 1994. The tension response to stretch of intact skeletal muscle fibres of the frog at varied tonicity of the extracellular medium. *J. Muscle Res. Cell Motil.* 15:145–157.
71. Cornachione, A. S., F. Leite, …, D. E. Rassier. 2016. The increase in non-cross-bridge forces after stretch of activated striated muscle is related to titin isoforms. *Am. J. Physiol. Cell Physiol.* 310:C19–C26.
72. Kaya, M., and H. Higuchi. 2010. Nonlinear elasticity and an 8-nm working stroke of single myosin molecules in myofilaments. *Science.* 329:686–689.
73. Capitanio, M., M. Canepari, …, R. Bottinelli. 2006. Two independent mechanical events in the interaction cycle of skeletal muscle myosin with actin. *Proc. Natl. Acad. Sci. USA.* 103:87–92.
74. Månsson, A. 2010. Actomyosin-ADP states, interhead cooperativity, and the force-velocity relation of skeletal muscle. *Biophys. J.* 98:1237–1246.
75. Edman, K. A. 1980. The role of non-uniform sarcomere behaviour during relaxation of striated muscle. *Eur. Heart J.* 1 (*Suppl A*):49–57.
76. Offer, G., and K. W. Ranatunga. 2013. A cross-bridge cycle with two tension-generating steps simulates skeletal muscle mechanics. *Biophys. J.* 105:928–940.
77. Offer, G., and K. W. Ranatunga. 2015. The endothermic ATP hydrolysis and crossbridge attachment steps drive the increase of force with temperature in isometric and shortening muscle. *J. Physiol.* 593:1997–2016.
78. Nocella, M., M. A. Bagni, …, B. Colombini. 2013. Mechanism of force enhancement during stretching of skeletal muscle fibres investigated by high time-resolved stiffness measurements. *J. Muscle Res. Cell Motil.* 34:71–81.
79. Fusi, L., M. Reconditi, …, G. Piazzesi. 2010. The mechanism of the resistance to stretch of isometrically contracting single muscle fibres. *J. Physiol.* 588:495–510.
80. Sakamoto, T., J. Limouze, …, J. R. Sellers. 2005. Blebbistatin, a myosin II inhibitor, is photoinactivated by blue light. *Biochemistry.* 44:584–588.
81. Capitanio, M., M. Canepari, …, F. S. Pavone. 2012. Ultrafast force-clamp spectroscopy of single molecules reveals load dependence of myosin working stroke. *Nat. Methods.* 9:1013–1019.
82. Yanagida, T., A. Ishijima, …, Y. Harada. 1993. Coupling between ATPase and force-generating attachment-detachment cycles of actomyosin in vitro. *Adv. Exp. Med. Biol.* 332:339–347, discussion 347–339.
83. Sommese, R. F., J. Sung, …, J. A. Spudich. 2013. Molecular consequences of the R453C hypertrophic cardiomyopathy mutation on human β-cardiac myosin motor function. *Proc. Natl. Acad. Sci. USA.* 110:12607–12612.
84. Várkuti, B. H., Z. Yang, …, A. Málnási-Csizmadia. 2012. A novel actin binding site of myosin required for effective muscle contraction. *Nat. Struct. Mol. Biol.* 19:299–306.
85. Brizendine, R. K., G. G. Sheehy, …, C. R. Cremo. 2017. A mixed-kinetic model describes unloaded velocities of smooth, skeletal, and cardiac muscle myosin filaments in vitro. *Science.* 3:eaao2267.
86. Brizendine, R. K., D. B. Alcala, …, C. R. Cremo. 2015. Velocities of unloaded muscle filaments are not limited by drag forces imposed by myosin cross-bridges. *Proc. Natl. Acad. Sci. USA.* 112:11235–11240.
87. Walcott, S., D. M. Warshaw, and E. P. Debold. 2012. Mechanical coupling between myosin molecules causes differences between ensemble and single-molecule measurements. *Biophys. J.* 103:501–510.

Biophysical Journal, Volume *115*

Supplemental Information

Blebbistatin Effects Expose Hidden Secrets in the Force-Generating Cycle of Actin and Myosin

Mohammad A. Rahman, Marko Ušaj, Dilson E. Rassier, and Alf Månsson

Supplementary Theory

ATP-hydrolysis, cross-bridge attachment and phosphate release

The processes, involving the recovery stroke and hydrolysis of ATP to ADP and inorganic phosphate (Pi) are lumped together into one transition between the M*T and M**DP states (Fig. 4; asterisks related to tryptophan fluorescence) with forward and reverse transition governed by rate constant k_3 and k_{-3}, respectively. Myosin heads in the M**DP state are assumed to bind weakly and non-stereospecifically to actin forming an AM**DP state with equilibrium constant $K_w(x)$:

$$K_w(x)=\exp(\Delta G_w-(ksw/2)(x-x_w)^2/k_BT) \quad \text{if } x\in[-1.3,16.7]\text{nm} \qquad \text{(S1a)}$$

$$K_w(x)=0 \qquad \text{for other x-values} \qquad \text{(S1b)}$$

where, ksw is the stiffness of myosin cross-bridges in the AM**DP state and x_w is the value of x where the free energy of binding (ΔG_w) of the AM**DP state attains its minimum (Table S1). Here, ΔG_w is in units of k_BT where k_B is the Bolztmann constant and T is the absolute temperature. The invididual rates are assumed to be infinitely high. Strictly, the weakly bound state, AM**DP, is not required in the model if ksw is very low as suggested by the lack of evidence for friction forces.

The transition from the weakly and non-stereospecifically bound AM**DP state to a weakly but stereospecifically attached AM*´DP state is governed by the rate function:

$$k_{on}(x) = k_{on}' \exp(\Delta G_{on} - ks\,(x-x_w)^2/\,k_BT + ksw(x-x_w)^2/\,k_BT) \qquad \text{(S2)}$$

where ΔG_{on} is the difference between the free energy minima of the states AM**DP and AM*´DP (see further, Table S1).

The reversal of this process is governed by:

$$k_{on\text{-}rev}(x) = k_{on}' \exp(ks\,(x-x_w)^2/k_BT\; ksw(x-x_w)^2/k_BT) \qquad \text{(S3)}$$

Next, a structural change is assumed to occur to allow the myosin head to enter the AM*DP state from which phosphate is released (1). Following, Llinas et al. (1), this transition is assumed to be associated with slightly increased affinity between actin and myosin and a small swing of the myosin head or lever arm as reflected in the vertical and horizontal shifts, respectively of the corresponding free energy diagrams (main Fig. 4B). After transition into the AM*DP state, phosphate is assumed to be rapidly and reversibly released from the active site. By now sharing the strain-dependence (cf. (2)) between the forward and backward rate functions and further assuming that phosphate release is a rapid equilibrium, the following rate functions emerge:

$$k_{P+}(x)= k_{P+}'\exp(\Delta G_{AM^*DP\text{-}AM^{**}DP}/2 \;-(ks/2)(x-x_1)^2/\,(2k_BT) +(ks/2)(x-x_w)^2/(2k_BT))) \qquad \text{(S4)}$$

$$k_{P-}(x)=k_{P+}'[Pi]/([Pi]+Kp) \exp(\Delta G_{AM^*DP\text{-}AM^{**}DP}/2+(ks/2)(x-x_1)^2/(2k_BT)-(ks/2)(x-x_w)^2/(2k_BT)) \qquad \text{(S5)}$$

From $-\ln(k_{P+}(x)/k_{P-}(x))$, the free energy difference between the states AM*´DP and AM*D_L is given by:

$\Delta G_{AM*'DP\text{-}AM**DL} = \Delta G_{AM*'DP\text{-}AM*DP} - k_BT\ln([Pi]/Kp) + (ks/2)((x-x_w)^2 - (x-x_1)^2)/k_BT)$ (S6)

Main force-generating transition

The main force-generating transition i.e. the power-stroke is, assumed to be a rapid equilibrium. The start of the power-stroke state ($AM*D_L$) has an open actin binding cleft and the lever arm in the pre-stroke position and the post-power-stroke state ($AM'D_H$) has the actin binding cleft closed and the lever-arm in the post-power-stroke position. The equilibrium constant is given by:

$$K_{LH}(x) = k_{LH+}(x)/k_{LH-}(x) \quad \text{(S7)}$$

where

$$k_{LH+}(x) = k_{LH-}(x)\exp(\Delta G_{AM*D\text{-}AM'D} + (ks/2)(x-x_1)^2/(k_BT) - (ks/2)(x-x_2)^2/(k_BT)) \quad \text{(S8)}$$

and

$$k_{LH-}(x) = 2000\ s^{-1} \quad \text{(S9)}$$

If Keq(i)>1000 according to Eq. S5, it was, for practical reasons, set equal to 1000 in Monte-Carlo simulations.

Cross-bridge detachment at the end of the power-stroke

The cross-bridge detachment occurs in several steps (3-5). First, there is a transition (6, 7) between the $AM'D_H$ and an AMD state that opens the nucleotide pocket for MgADP release (5, 7):

$$k_5(x) = k_5(x_1)\exp(\delta G)\cdot \exp\left(\frac{k_s\cdot|x|\cdot\delta x}{k_BT}\right) \quad \text{(S10)}$$

where $\delta x = x_2 - x_3$ (Table S1) corresponds to the difference in position for the free energy minima of the $AM'D_H$ and AMD states. Further, $\delta G = \Delta G_{AM'D\text{-}AMD} + (k_s/2)(x_2^2 - x_3^2)/k_BT$, i.e. the difference in free energy between the $AM'D_H$ and the AMD states states at x=0 nm.

The next steps involve MgADP release and MgATP binding and the AMD, AM and AMT states are lumped together, giving the following rate function ($k_{off}(x)$; (8)) for the transition from the AMD to the MT state (assuming [MgADP] = 0 mM):

$$k'_{off}(x) = \frac{k_2(x)k_6[MgATP]}{\frac{k_6}{K_1} + (k_2(x)+k_6)[MgATP]} = \frac{k_2(x)[MgATP]}{\frac{1}{K_1} + \frac{k_2(x)}{k_6}[MgATP] + [MgATP]} \quad \text{(S11)}$$

where

$$k_2(x) = k_2(0)\exp\left(\frac{k_s\cdot|x|\cdot x_{crit}}{k_BT}\right) \quad \text{(S12)}$$

Here, $k_2(0)$ and k_6 govern ATP induced detachment from the AMT state at x=0 and strain-independent ADP-release from the AMD state, respectively. The constant K_1 is the equilibrium constant for MgATP binding to the AM state (Fig. 3A) and x_{crit} is a strain parameter (9) that defines strain-dependence of the MgATP induced detachment. An overall detachment rate function, from the $AM'D_L$ state to the M**T state, is given by:

$$k_{off}(x) = \frac{k'_{off}(x) k_5(x)}{k'_{off}(x) + k_5(x)} \quad \text{(S13)}$$

The latter rate function, together with Eqs. S11-S12 can be used for simplifying the current model, allowing detachment into the M**T state directly from the AM´D_L state (Fig. S8).

There has long been evidence that the myosin-actin interaction and its relation to ATP turnover differ appreciably between elongation and shortening of active muscle (10-13). The findings imply that a large fraction of the attachment-detachment cycles during elongation are very rapid and occur without consumption of ATP. This picture is captured by the present model: First, during stretch, a majority of the cross-bridges are forcibly detached from states (particularly the AM*D_L state) prior to the main force-generating transition without turnover of ATP (Eqs. S3 and S5). Second, cross-bridges detached from the AM*D_L state are assumed to instantaneously reattach into this state at a neighboring actin site 5.5 nm toward the pointed end of the actin filament (cf. similar model for shortening in (14)). The overall detachment-reattachment process is governed by the rate function:

$$k_{\text{off-str}}(x) = k_{off-str}(x_{11}) \cdot \exp\left(\frac{k_s \cdot |(x - x_{11}| \cdot \delta x_{str}}{k_B T}\right) \quad \text{(S14)}$$

where $\delta x_{str} = 2.7$ nm. Whereas the present model rather well approximates the early phase of the force response to stretch there are limitations in the fitting of later phases. This is attributed to the presence of elastic elements in parallel with the cross-bridges (15, 16) e.g. titin, and sarcomere non-uniformities (16-19) affecting the tension response in muscle cells. It may also reflect uncertainties on how the exact values of certain kinetic constants affect the stretch response. For instance, we have tentatively used parameter values for $k_{off-str}(x_{11})$ and δx_{str} identical to those for forcible detachment of rigor cross-bridges (20).

Contractile activation, filament compliance and target zones on actin filaments

Just as in the recent model (3) we assume maximum Ca^{2+} activation and, unless otherwise stated, that a single site on the actin filament is within reach of a given myosin cross-bridge. Furthermore, we assume that only one of the globular myosin units (heads) in a pair binds simultaneously to a given target zone and that neighboring zones are separated by 36 nm. Finally the actin filaments and the myosin attachment points are assumed infinitely stiff.

Comments on parameter values, relation to experimental data and model limitations

The numerical values of the cross-bridge stiffnesses for strongly and weakly bound cross-bridge states has been motivated previously as well as the location of the free-energy minima of a majority of the states (3). The free-energy minimum of the new pre-power-stroke state (AM*´DP) as well as the weakly bound AM**DP state was here assumed to occur at 8.7 nm, consistent with a small structural change (1) upon transition into the Pi-release state (AM*DP) with free energy minimum at x=7.7 nm.

The present model fits a range of data in the absence of blebbistatin. Thus, the maximum sliding velocity for long filaments is 9.4 µm/s similar to our experimental values of 8-11 µm/s in the temperature range from 28-31 °C. During steady-state isometric contraction the model predicts an average force per cross-bridge of 6 pN, similar to values from experiments on single molecules from fast mammalian muscle (21). Additionally, the predicted force enhancement during stretch, relative the isometric force, is similar to experiments (cf. this study). Also the Vmax and K_{ATPase} values (84 s^{-1} and 0.4 mM) of the actomyosin ATPase are

similar to appropriately temperature corrected (3) experimental data at close to physiological ionic strength used here (cf. (22)). The K_M^v value for the velocity vs [MgATP] is about 5-fold lower than in experiments. Whereas this could be amended by making the cross-bridge stiffness non-linear with low stiffness for cross-bridge strains that counteract sliding (3, 8) we did not introduce this complication because the details are poorly understood (cf. (8)). Finally, the relationship between [Pi] on the one hand and tension and velocity on the other hand are consistent with results in the litterature (Fig. S1).

Possible effects of uncertainties in parameter values

In our simulations we use parameters fixed at literature values only changing the parameter value of relevance for testing a particular hypothesis on basis of blebbistatin effects on the actomyosin ATP turnover rate. If the simulation results strongly depend on the exact parameter values that are used, erroneous conclusions may emerge as a result of uncertainties in these values. It is therefore desirable with an error propagation analysis where errors in each parameter value propagate through the analysis to investigate how these errors affect the conclusions. Unfortunately this approach suffers from two problems. First it is, for several reasons, difficult to obtain reliable and/or consistent error estimates of different parameters values from the litterature. For this reason, we assigned a reasonable lower and higher bound of the parameter value corresponding to 75 % and 125 % respectively of the mean value used. The second problem that prevents a full error propagation analysis is of combinatorial origin. Thus, if all combinations of lowest (mean value – error) and highest (mean + error) parameter value would be tested for each of the 25 parameters in Tables S1-S2, this would lead to 2^{25} 33 000 000 simulation runs. This is clearly incompletely unrealistic considering that some of the individual Monte-Carlo simulations (e.g. for length velocity plots) take several hours.

Nevertheless, in an effort to test the robustness of our conclusions by an approach related to a full investigation of the type considered above, we performed a limited error propagation analysis based on random sampling. First, we assumed (see above) that each parameter value is either 25 % higher or 25 % lower than the mean value currently used and then we andomly assigned either the lower value or the higher value to each parameter. If this assignment resulted in a priori unreasonable value of any parameter, e.g. giving unreasonable free energy profiles, the closest reasonable parameter value was used. "Unreasonable" free energy profiles are either inconsistent with the free energy of ATP-turnover or the x-values for minima of the free energy are increased rather than decreased for sequential states from attachment towards detachment. Following this corrective step, the selected combinations of low and high parameter values were used to simulate the physiological force-velocity relationship because such simulations and the associated analysis could be conducted in minutes. Next, the sets of selected parameter values that gave reasonable shapes of the force-velocity relationship and three other set where used as basis for testing the two major models (change of kp+(x) or change of k_{on}(x).

Simulations of contraction and actomyosin interactions at 5 ^oC

In our treatment we focused on the parameters $k_{3,}$ k_{on}(x), k_2 and ΔG_{LH} for which there is evidence for significant temperature effects. These parameter values were changed to those given in Table S3 for the simulation of isometric tension and the tension response to active stretch at 5 ^oC. Other parameter values were left at those in Tables S1-S2. The rate constants k_3 and k_{on}(x) were assumed to have Q_{10} values of ~4 in agreement with high temperature sensitivity of these processes found previously (23-27). The free energy difference ΔG_{LH} (see below) was reduced from 14 k_BT at 25-30°C to 6 k_BT at 5 ^{o}C in order to account for the >50 % reduction of the average cross-bridge strain during isometric contraction of rabbit psoas fibers under these conditions (27, 28). The temperature sensitivity of the cross-bridge

detachment rate after the end of the power-stroke is primarily conferred by the rate constant k_2 with Q_{10} ~2 (29). In our simulations we tentatively assumed Q_{10} =2 for kp+(x).

The above changes in parameter values gave an approximate Q_{10} value of 3-4 for the maximum actin-activated ATP turnover rate (in the range 5 to 25-30 ^{o}C) somewhat lower than the experimental value of 5 (22). In addition, the change in parameter values predicted 2-3x increase of the maximum isometric force for an increase in temperature from 5 to 30 ^{o}C (Q_{10} ~1.3-1.6) similar to experimentally observed effects (3-4x increase; Q_{10} ~1.5-1.9;(27, 30, 31)). The predicted maximum velocity of shortening was increased 9-fold (Q_{10} ~2.4) quite similar to experimental data with Q_{10} ~2.0 (32). The maximum force during stretch was little affected (increase by 8 %) by the change in parameter values corresponding to increase in temperature from 5 to 25-30 ^{o}C. This small effect of temperature on the force during stretch is consistent with experimental results (30).

Supplementary Results

Ionic strength effects

The lower effect of blebbistatin on the sliding velocity at reduced ionic strength may have different grounds. The possibility that the affinity of blebbistatin to myosin is reduced at lower ionic strength is contradicted by similar effect of ionic strength on the fractional inhibition at 1 and 30 µM blebbistatin. The remaining possibilities include 1. different local effects of blebbistatin on myosin structure at low and high ionic strength or 2. modulated blebbistatin effects by different ionic strengths due to different steady-state distributions between different actomyosin states under these conditions. However, Monte-Carlo simulations suggest that the blebbistatin effect on velocity by the favored mechanism in the main paper is negligibly attenuated by 12-fold increased affinity (23) for the weak-binding state (AM**DP) corresponding to a reduction in ionic strength from 130 mM to 60 mM. However, appreciably increased actin affinity in the Pi-release state (AM*DPi) upon lowered ionic strength would enhance the attenuating effect of this intervention so that velocity reduction in response to 1 µM blebbistatin would be 35-40 % at 60 mM ionic strength compared to 45 % at 130 mM strength. This accords with the idea (1) that the actin-binding of myosin in the Pi-release state (AM*DP state in the present model) is to a large extent mediated by ionic interactions. However, it is premature currently to consider this issue in detail due to lack of quantitative information about possible changes in actin affinity in the AM*DP state.

Possible effects of uncertainties in parameter values

Random change of all parameter values in Table S1 and S2 either up or down by 25 % (values used shown in Table S5) caused, in a majority of the cases (7/9), the force-velocity relationship to deviate appreciably from the experimental force velocity data (Fig. S9). In some cases (2/9) the force-velocity relationship was quite similar to that observed experimentally. For these cases as well as one case were the parameter values were changed away from the random selections to give better fit to the force-velocity data we performed a full set of simulations of the blebbistatin effects assuming that blebbistatin either reduced $k_{p+}(x)$ or $k_{on}(x)$. Similar simulations were also performed for three randomly selected sets of parameter values giving poor fits to the force-velocity data The results of these simulations are illustrated in Fig. S11 showing a comparison between the sum of squared deviations for all parameter values in Table S4 for the two different models. It is clear from Fig. S11 that, for all sets of parameter values tested the sum of the squared deviations between model and experiments were smallest for the model assuming that blebbistatin reduces $k_{p+}(x)$.

Supplementary Methods

Chemicals and Materials

Blebbistatin [(+/-)-1-Phenyl-1,2,3,4-tetrahydro-4-hydroxypyrrolo[2,3-b]-7-methylquinolin-4-one] was purchased from Toronto Research Chemical (cat. no.TRC-B592490-10) for the in vitro motility assays. Other chemicals were of analytical grade and purchased from Sigma Aldrich except Rhodamine Phalloidin that was from Thermo Fisher Scientific (cat. no. R415).

Protein preparations

Actin, myosin and HMM were prepared from fast skeletal muscle of New Zealand white rabbits (33, 34). To obtain myosin with phosphorylated RLCs (resulting in partial phosphorylation; pP-myosin),minced muscle was extracted for 20 min in 60 ml/20g ice cold Guba Straub solution (0.3 M KCl, 0.1 M KH_2PO_4, 0.05 M K_2HPO_4; pH 6.5). The extract was then centrifuged at 11000 g for 30 min at 4^0C. The supernatant was filtered through two layers of gauze followed by addition of 14 volumes of ice cold buffer A (5 mM potassium phosphate buffer, pH 7.0; with 0.1 mM DTT). After 2 hours incubation, the precipitated myosin filaments were collected by centrifugation at 11000 g for 18 min at 4^oC. The pellet was re-suspended in 2-3 ml of buffer B (20 mM potassium phosphate buffer, pH 8.0; with 0.5 M KCl, 5mM Na_2ATP, 12.5 mM $MgCl_2x6H_2O$ and 0.1 mM $CaCl_2x2H_2O$). The above procedure was repeated in obtaining dephosphorylated myosin (dP-myosin) but buffer A was exchanged for buffer C (1 mM EDTA and 0.1 mM DTT) and buffer B for buffer D (20 mM MOPS, 1 mM DTT and 0.5 M KCl; pH 7.0). In preparation of pP-myosin, the solution (pellet re-suspended in buffer B or D) was next kept for 30 minutes at room temperature followed by ultracentrifugation at 120 000 g for 2 hours and 30 minutes at 4^oC. The supernatant was then precipitated with 14 volumes of degassed ice-cold distilled water for 30 minutes at 4^oC followed by an additional centrifugation at 6500 g for 10 minutes at 4^oC. In the preparation of pP-Myosin, the precipitate in the final step was re-suspended in buffer B and ultracentrifugation was repeated with collection of the supernatant that was then used immediately for HMM preparation. In the preparation of dP-myosin, 1 ml of buffer D was added to the pellet followed by storage on ice overnight, to ensure that myosin is fully dephosphorylated. On the next day, the pellet was re-suspended in 1-2 ml of buffer D and ultracentrifugation was repeated followed by HMM preparation.

Gel electrophoresis

The purity and integrity of actin, myosin and heavy meromyosin were confirmed by SDS-PAGE (ThermoFisher Scientific; cat. no. NP0342BOX). The level of myosin RLC phosphorylation was analyzed by 8 M Urea PAGE slightly modified from previous work (35) by using 25 mM Tris-Glycine running buffer containing 6 M Urea.

In vitro motility assays

In vitro motility assays (IVMA) were performed (8) by adsorbing HMM to coverslips silanized with trimethylchlorosilane (TMCS). Assay solutions were prepared in buffer E (10 mM MOPS, 1 mM $MgCl_2$, 0.1 mM K_2EGTA; pH 7.4). Buffer F (10 mM MOPS, 50 mM KCl, 1 mM DTT, 1 mM $MgCl_2$, 0.1 mM K_2EGTA) was used to rinse the flow cells and for diluting HMM and F-Actin. In an in vitro motility assay, the flow cell was first incubated with HMM (30 µg/ml or 120 µg/ml) for 2-5 minutes followed by incubation and washing steps as follows: 1mg/ml BSA (2 min), buffer F (30 s), 1µM blocking actin (non-fluorescent actin filaments in buffer F; 1-2 min), 1mM MgATP in buffer F (30 s), 2 x buffer E (30 s each), 2-10 nM rhodamine-phalloidin labeled actin filaments, buffer E (30 s) prior to initation of the assay. Assay solution (10 mM DTT, 45-135 mM KCl, 3 mg/ml Glucose, 0.1 mg/ml glucose oxidase, 0.02 mg/ml catalase, 2.5 mM creatine phosphate, 0.2 mg/ml creatine phosphokinase,

0.01-1 mM MgATP) was prepared with methyl cellulose (0.64%) in buffer E if the ionic strength was ≥ 80 mM. In some experiments, the incubation step with blocking actin was not included, e.g. in cases when velocity was studied vs. filament length Blebbistatin (mixed enantiomer) was dissolved in N,N-Dimethylformamide (DMF; Sigma Aldrich; cat. no. 227056) to a final concentration of 16.27 mM whereas S-(-) blebbistatin was dissolved in dimethyl sulfoxide to a final concentration of 17.91 mM. Both were aliquoted and stored in the dark (-20^{0}C). As suggested by control experiments at the final concentrations used, DMF per se, did not affect the HMM induced actin filament sliding in the in vitro motility assay. The blebbistatin concentration was based on absorbance spectrophotometric analys using an exctinction coefficient of 7400 M^{-1} cm^{-1} at a wavelength of 422 nm. The blebbistatin aliquot was diluted in buffer E, 1 hour prior to the in vitro motility assays. The flow cells were incubated with blebbistatin for 15 minutes (36) before adding the assay solution with the same blebbistatin concentration. Prolonged illumination with the wavelength used for visualizing the rhodamine phalloidin labelled actin filaments did not affect the results.
Actin filament movements were recorded using an electron multiplying charge coupled device (EMCCD) camera (C9100-12, Hamamatsu Photonics) with a frame rate in the range 4-10 f/s. In the data analysis, actin filament sliding velocities were calculated as described (Månsson & Tågerud, 2003) earlier. At low velocities (either due to a high blebbistatin concentration or a low [MgATP]) every 3rd or 4th frames were considered until in total 15 frames were analyzed. The cut-off of the coefficient of variation (CV) (standard deviation of frame-to-frame velocity divided by average velocity in ten frames) (Månsson & Tågerud, 2003) for inclusion of data in velocity analysis varied between 0.2 and 0.4 with negligible effects on the results. For analysis of velocity vs length plots, all data was used independent of the CV value and the average velocity was calculated over 2.6-10.4 s, with the shortest time for the highest velocities (~10 µm/s) and the longest time for the lowest velocities (< 2 µm/s). This was important because simulations indicated that some models for the blebbistatin effect would reduce average velocity by producing pauses in filament sliding. Such effects would not be captured by analysis of the experimental data if CV based cut-offs had been used.

Muscle fiber experiments
Muscle bundles of rabbit psoas were dissected and permeabilized following standard procedures (37). Muscles were incubated in rigor solution (pH = 7.0) for ∼4 h, after which they were transferred to a rigor-glycerol (50:50) solution for ~15 h. The samples were subsequently placed in a fresh rigor-glycerol (50:50) solution with the addition of a cocktail of protease inhibitors (Roche Diagnostics) and stored in a freezer (−20°C) for at least 7 days. On the day of the experiment, a small section of the sample was cut (∼4 mm in length), and single fibers were dissected in relaxing solution (see below). The fibers were gripped at their ends with T-shaped clips made of aluminum foil and were transferred to a temperature-controlled chamber to be attached between a force transducer (resonant frequency 1 kHz) (model 403A, Aurora Scientific, Toronto, ON, Canada) and a length controller (model 312B, Aurora Scientific). The rigor solution (pH 7.0) was composed of (mM) 50 Tris, 100 NaCl, 2 KCl, 2 $MgCl_2$, and 10 EGTA. The relaxing solution used for muscle storage and dissection (pH 7.0) was composed of 100 KCl, 2 EGTA, 20 imidazole, 4 ATP, and 7 $MgCl_2$. The experimental solutions with pCa^{2+} of 4.5, 5.0, 5.5, and 6.0 (pH 7.0) contained 20 imidazole, 14.5 creatine phosphate, 7 EGTA, 4 MgATP, 1 free $Mg^{2+,}$ free Ca^{2+} ranging from 1 nM (pCa^{2+} 9.0) to 32 µM (pCa^{2+} 4.5), and KCl to adjust the ionic strength to 180 mM. A pre-activating solution: 68 KCl, 0.5 EGTA, 20 imidazole, 14.5 creatine phosphate, 4.83 ATP, 0.00137 $CaCl_2$, 5.41 $MgCl_2$ and 6.5 HDTA; pH 7.0, pCa^{2+} 9.0) with a reduced Ca^{2+} buffering capacity was used immediately before activation. Blebbistatin was dissolved in dimethylformamide (DMF) and was stored at −20°C before use. On the day of the

experiment, blebbistatin was diluted in 4 ml of activating (pCa^{2+} 4.5) or relaxing (pCa^{2+} 9.0) solution to reach final concentrations of 1, 2, 5 or 10μM. A red filter (650 nm) was placed on the light source of the microscope to avoid exposure of blebbistatin to light during the experiments, as it loses its effectiveness in wavelengths between 365 and 490 nm (38).

Fibers were activated in the presence or absence of blebbistatin (n = 16). All experiments were performed at 5°C. The initial SL was adjusted to ~2.5 μm (optimal length, *L*o) before fiber activation. The fibers were first activated at a pCa^{2+} of 4.5 and stretched by 5 or 10% *L*o, at difference velocities from 0.4-2 *L*o·SL·s−1. After that, the fibers were incubated in relaxing solution (pCa^{2+} = 9.0) containing blebbistatin. After blebbistatin incubation in relaxing solution (15 min), the fiber was immersed in activating solution also containing blebbistatin. After full force development, similar stretches as in the absence of blebbistatin were applied to the fibers. Control contractions at a pCa^{2+} of 4.5 were elicited through the experiments; at the end of the experiments the isometric forces never decreased by >10% (actual range: 5.2–8.3%) from the maximal force produced at the beginning of the experiment (Po). When the striation pattern of the muscle fibers became unclear such that it did not allow measurements of SL, the experiments were ended.

The transition between the two phases of force rise during a stretch was detected with a two-segment piecewise regression (37).When the piecewise regression did not detect the transition point based on these criteria, we extrapolated the two lines visually to detect te breakpoint. Visual inspection provided results similar to regression analyses when both methods could be compared. The intersection between the two slopes representing the fast and slow increases in force was used to define Pc, calculated as the relative increase in force obtained from the maximal isometric force developed before stretch (Po) in any given condition.

Data analysis for muscle fiber experiments. Force was measured just before stretch (isometric force, F_{iso}) and at a breakpoint between two phases of force increase during stretch defining the critical force (F_c). The transition between the two phases was evaluated by differentiation of force during the stretch phase [d(force)/d*t*)](39).

Statistical analysis

Data are presented as mean ± 95 % confidence limits unless otherwise stated. N represents number of actin filaments for in vitro motilitya assays and the number of muscle fibers tested for muscle fiber experiments. All statistical analyzes and curve fittings in relation to experiments on isolated proteins and modelling were performed using the GraphPad Prism software (Version 6.07; GraphPad software Inc, USA).

Monte Carlo simulations

For the purpose of our simulations we made the simplifying assumption that HMM motor fragments are adsorbed to motility assay surfaces with uniform density, ρ (5000 μm^{-2}) and that myosin heads in a band of d=30 nm width around the long axis of the filament are available for binding (40). Then it was assumed that all 36 nm intervals along an actin filament are identical. That is we assumed that the total number of available myosin heads (n=ρdl) along the entire filament of length, l, is distributed uniformly between 360 bins each of 0.1 nm width (3). The simulations started with all myosin heads in the M**DP and M**DP-bleb states at the appropriate equilibrium ratio assuming a blebbistatin affinity of 1 μM^{-1} (41). The time, Δt, until the first/next update event was calculated using the Gillespie algorithm (42) from the inverse rate summed over all possible chemical transitions at each discret value, $x=x_{bin}$, as described previously (3). The simplifying assumption was made that

blebbistatin binds to myosin in the MDP or MT states with a dissociation constant K_B, and then undergoes obligatory dissociation from myosin in the AM´D_H state. This is assumption is essential in order not to overwhelm the computational power. In order to maintain a roughly constant blebbistatin-bound fraction of myosin, despite the assumed obligatory dissociation from the AM´D_H state, we assumed that the rates associated with the blebbistatin binding-equilibrium are orders of magnitude faster than found in experiments. The consequences of these simplifying assumptions were minimal as suggested by simulations where the rate was changed 100-fold.

Numerical solution of differential equations

For simulation of actomyosin ATPase in solution and estimates of V_{max} and K_M, for this relationship we used Simnon (version 1.3; SSPA, Gothenburg, Sweden) to numerically solve the system of differential equations for the kinetic scheme in main Fig. 4A. The rate constants were those at the minima of the free energy diagrams in Fig. 4B.

Table S1. Parameter values[a] determining shape of free energy diagrams for simulation of contractile properties of fast mammalian muscle at 25-30 °C

**Parameter**	**Numerical value**	**Range from litterature**	**Value used in testing hypothesis for blebbistatin effect**[b]	**References and comments**
x_w (AM**DP)	8.7 nm	Set equal to x_1	-	(1) Details in (3)
x_1 (AM*´DP)	8.7 nm	~1 nm higher than x_{11}	-	Based on small structural change between prepowerstroke state and Pi-release state
x_{11} (AM*D_L)	7.7 nm	~8 nm	-	(43)[b] Details in (3)
x_2 (AM*D_H)	1.0 nm	0.9-1.1 nm	-	(6)[c] Details in (3)
x_3	0 nm		-	By definition
ΔG_w (M**DP-AM**DP)	0 k_BT; corresponds to K_w=1	~0 k_BT	-	(44) Details in (3)
$\Delta G_{AM^{**}DP-AM^{*\prime}DP} \equiv \Delta \mathbf{G_{on}}$	0.7 k_BT	Due to surface loops	Model I: (Table S4): -4k_BT (reduces $k_{on}(x)$; Eq. S2)	
$\Delta G_{AM^{*\prime}DP-AM^{*}DP} \equiv \Delta \mathbf{G_P}$ (AM*´DP – AM*DP)	1 k_BT	Due to surface loops	-	(22, 45) and Q_{10} = 2.7-3.7 in range 20-35°C. Details in (3)
$\Delta G_{AM^{*}DL-AM^{\prime}DH} \equiv \Delta \mathbf{G_{LH}}$ (AM*D_L-AM*D_H)	14 k_BT	$\Delta G_{AM^{*}DL-AM'DH}$ + $\Delta G_{AM^{\prime}DH-AMD}$ (10-20 k_BT)	Model III: 2.5 k_BT	(43, 46) Details in (3)
$\Delta G_{AM^{*}DH-AMD}$ (AM*D_H AMD)	2 k_BT	See previous row; 1-2 k_BT	-	(8, 43) Details in (3)
ΔG_{ATP}	13.1 + ln ([MgATP] / ([MgADP] [Pi]) k_BT	Free energy of ATP-hydrolysis	-	(47)
ks	2.8 pN/nm	2.5-2.8 pN/nm	-	(43) [b]
ksw	0.02 pN/nm	-	-	Details in (3)

Footnotes to Table S1

NA: Not applicable IS: ionic strength

[a] The parameter values were from two-headed myosin motor fragments from fast skeletal muscle of rabbit at 25-30°C, ionic strength 130-200 mM, pH 7-8 unless otherwise stated.

[b] "-" means that control value was always used

Table S2. Parameter values[a] defining rate functions and kinetic constants for simulation of contractile properties of fast mammalian muscle at 25-30 ^oC

Parameter	Numerical value used	Litterature value(s)	Value used in testing hypothesis for blebbistatin effect	References and comments
$k_{+3} + k_{-3}$ (Recovery stroke+hydrolysis)	220 s^{-1}	200-500 s^{-1} Assuming Q_{10} in range 3-4 (23)	-	(23, 25) and references therein
K_3	10	2-10	-	(23, 25) and references therein. Details in (3)
k_{on}'	130 s^{-1}		-	(25, 45). Fine-tuned to fit rate of rise of force, V_{max} of actomyosin ATPase and phosphate transient
k_{P+}'	1000 s^{-1}		Model II: 1.5 s^{-1}	(20) High to ensure high maximum velocity (see text)
$k_{off\text{-}str}(x_{11})$	0.016 s^{-1}		-	(20) Forcible detachment of cross-bridges during stretch (see further text)
δx_{str}	2.7 nm		-	(20) Forcible detachment of cross-bridges during stretch
k_{-5}	2000 s^{-1}		-	Details in (3)
Kc	10 mM	~1-10 mM (temp corrected)	Model IV: 0.1-1000 (not analyzed in detail)	(45) Details in (3)
x_{crit}	0.6 nm	< 0.2 nm (see further, Theory)	-	(21) Details in (3)
k_6	5000 s^{-1}	>3500 s^{-1}	-	(29) Details in (3)
k_{-6}	14 290 mM^{-1} s^{-1}	>10 000 mM^{-1} s^{-1}	-	(29) Details in (3)
Physio-logical [Pi]	0.5 mM	~ 0.5 mM		(48)
K_1	1.7 mM^{-1}	1.7 mM^{-1}	-	(29) Details in (3)
k_2	2000 s^{-1}	1400 s^{-1}	-	(29) Details in (3). Higher value used to account for high maximum shortening velocity

Footnotes to Table S2

[a] The parameter values were from two-headed myosin motor fragments from fast skeletal muscle of rabbit at 25-30°C, ionic strength 130-200 mM, pH 7-8 unless otherwise stated.

[b]Fit of force velocity relationship. See text.

[c]From the assumption of a diffusion limited rate constant > 10 000 mM^{-1} s^{-1} (14 000 mM^{-1} s^{-1})

Table S3. Parameter values[a] for simulation of contractile properties of fast mammalian muscle at 5 ^{o}C

**Parameter**	**Numerical value used for control simulations**	**Value used in testing hypothesis for blebbistatin effect**	**References and comments**
Δ **G$_{on}$**	0.7 k_BT	Model I: -4 k_BT	
$\Delta G_{AM^*DL-AM'DH} \equiv$ Δ **G$_{LH}$** (AM^*D_L-AM^*D_H)	6 k_BT	-	(28) Temperature induced decrease in average cross-bridge strain during isometric contraction in (28) requires decrease in the parameter value for effect to be accomodated by the present model
$k_{+3} + k_{-3}$ (Recovery stroke+hydrolysis)	12.5 s^{-1}	-	(24, 25) and references therein
K_3	4	-	(24, 25) and references therein
k_{on}'	25 s^{-1}	-	(24, 25) and references therein
k_2	413 s^{-1}	-	(29)
k_{P+}'	250 s^{-1}	Model II: 0.4 s^{-1}	

[a]Parameter values not given here are assumed identical to those given in Tables S1-S2

Table S4. Effects of critical changes in model parameters according to three key models, compared to experimentally observed blebbistatin effects[a]

**Variable**[b] (B: blebbistatin)	**I. ΔG_{on} from 0.7 to -4 k_BT (reduced $k_{on}(x)$)**[c]	**II. k_{p+}' down from 1000 to 1.5 s^{-1}**[d]	**III. k_{p+}' down from 1000 to 1.5 s^{-1} and ΔG_{LH} from 14 to 2.5 k_BT**[e]
Velocity > 2 µm long filaments (1 µM B)	***0.1842*** **(+)**	8.86 10^{-5} (+)	6.13 10^{-5} (+)
Velocity (0.5-1.5 µm filament; [B] with close to half maximum vel.)	***0.1062 (+)***	0.0005327 (+)	0.0003556 (+)
Velocity > 2 µm long filaments(10-30 µM B)	***0.1951*** **(+)**	0.04171 (+)	0.04227 (+)
Velocity (0.5-1 µm filament; [B] with half maximum velocity)	0.000897 (+)	0.03013 (+)	0.03548 (+)
Velocity (1 µM B; 0.01 mM MgATP)	0.08697 **(-)**	0.002567 (+)	0.001380 (+)
Velocity vs [MgATP], K_M (1 µM B)	*0.071289* (+)	0.0619 (+)	0.03378 (+)
Isometric force (2 µM B)[g]	0.004976 (+)	0.004112 (+)	0.01852 (+)
Isometric force (10 µM B)[g]	0.01233 (+)	0.003812 (+)	0.0002171 (+)
Force enhancement during stretch (2 µM B)[g]	***0.1501 (-)***	***0.09359 (-)***	0.02182 (-)
Force enhancement - stretch (10 µM B)[g]	***0.1054 (+)***	***0.01084 (+)***	9.831 10^{-5} (+)
V_{max}:Actomyosin – ATPase (saturating B)	0.000015 (+)	<1 10^{-6} (+)	<1 10^{-6} (+)
K_{ATPase} (saturating B)	***3.91*** **(-)**	***0.192811 (+)***	***0.192811 (+)***
Total SS[f]	**4.8284**	**0.4421**	**0.3468**

[a] Numerical values refer to squared difference between fractional blebbistatin/control values in model and experiments. Changes in the model are in same (+) or different (-) direction compared to experiments. Numbers in both bold and italics indicate particularly poor fits. One HMM preparation and one batch of blebbistatin used in the experiments to allow use of one value of the blebbistatin affinity (K_B=1 μM^{-1}) in the simulations. The velocity for short (0.5-1.5 µm) filaments was an exception where different blebbistatin batches were used. The blebbistatin concentration studied in this case was that which lowered the maximum velocity by 50 %.

[b] 1 mM MgATP, 130 mM ionic strength and long filaments (>5 µm) unless otherwise stated for in vitro motility assay data.

[c] Corresponds to blebbistatin-inhibition of normal rate limiting step of actomyosin ATPase.

[d] Corresponds to blebbistatin-induced inhibition of the transition from the AM*´DP state to the Pi-release state (AM*DP)

[e] Corresponds to blebbistatin-induced inhibition of the main force-generating transition.

[f] Statistical analysis using Friedman´s test for pairwise comparison of squared deviations, followed by Dunn´s post hoc tests, suggests that hypotheses 2 and 3 are better than hypothesis 1 ($p \approx 0.043$ and $p \approx 0.007$, respectively) whereas there is no significant difference between hypotheses 2 and 3.

[g] For both stretch and isometric force the simulated data that were employed in this analysis assumed high-temperature parameter values (Tables S1-S2) as only relative differences between blebbistatin data and control data are considered.

Table S5: Parameter values that were varied and used for the simulations in Fig. S9 testing effects of errors in literature parameter values[a]

	1	2	3	4	5	6	7	8	9	10[b]
K_{+3}	150	150	250	150	150	150	250	150	150	150
k_{-3}	15	15	15	15	25	25	25	15	15	25
k_{on}'	97.5	97.5	162.5	162.5	97.5	162.5	97.5	97.5	97.5	97.5*
k_{P+}'	1250	1250	750	1250	750	1250	1250	1250	1250	1250*
k_{-5}	1500	2500	1500	1500	1500	2500	2500	1500	2500	1500
Kc	12.5	7.5	12.5	12.5	7.5	7.5	12.5	7.5	12.5	7.5
x_{crit}	**0.6**	0.45	0.45	0.45	0.75	0.75	0.75	0.75	0.75	0.75
k_6	5000	5000	3500	3500	5000	3500	3500	5000	3500	5000
K_1	2.125	1.275	1.275	2.125	2.125	1.275	2.125	1.275	1.275	2.125
k_2	1500	1500	2500	1500	2500	2500	1500	1500	2500	2500
x_1	0.75	1.25	0.75	0.75	0.75	0.75	1.25	1.25	0.75	0.75
x_{11}	0	0	0	0	0	0	0	0	0	0
x_2	-8.375	-8.375	-5.025	-8.375	-8.375	-8.375	-5.025	-8.375	-5.775	-8.375
x_3	-9.625	-9.625	-5.775	-8.375	-9.625	-9.625	-5.775	-9.625	-5.775	-9.625
ΔG_{on}	0	0	2	2	0	0	0	2	2	0
ΔG_P	0	2	2	2	0	2	2	2	2	0
ΔG_{LH}	10.5	18	18	18	10.5	18	18	18	10.5	14*
$\Delta G_{AM*DH-AMD}$	2.5	2.5	1.5	1.5	2.5	1.5	1.5	2.5	2.5	2.5
ks	2.1	3.5	3.5	3.5	2.1	2.1	3.5	3.5	3.5	2.1

[a]Parameter values in red fonts gave bad fits to experimental force-velocity data (see Fig. S9)

[b]Numerical values in this column (10) not obtained by random selection. Instead, parameter values in column 10 are identical to those in column 5 except for the fact that the parameters labelled with asterisk have been modified to improve the fit of the force-velocity relation in the absence of blebbistatin. This approach was taken in order to obtain three sets of parameter values with good fit.

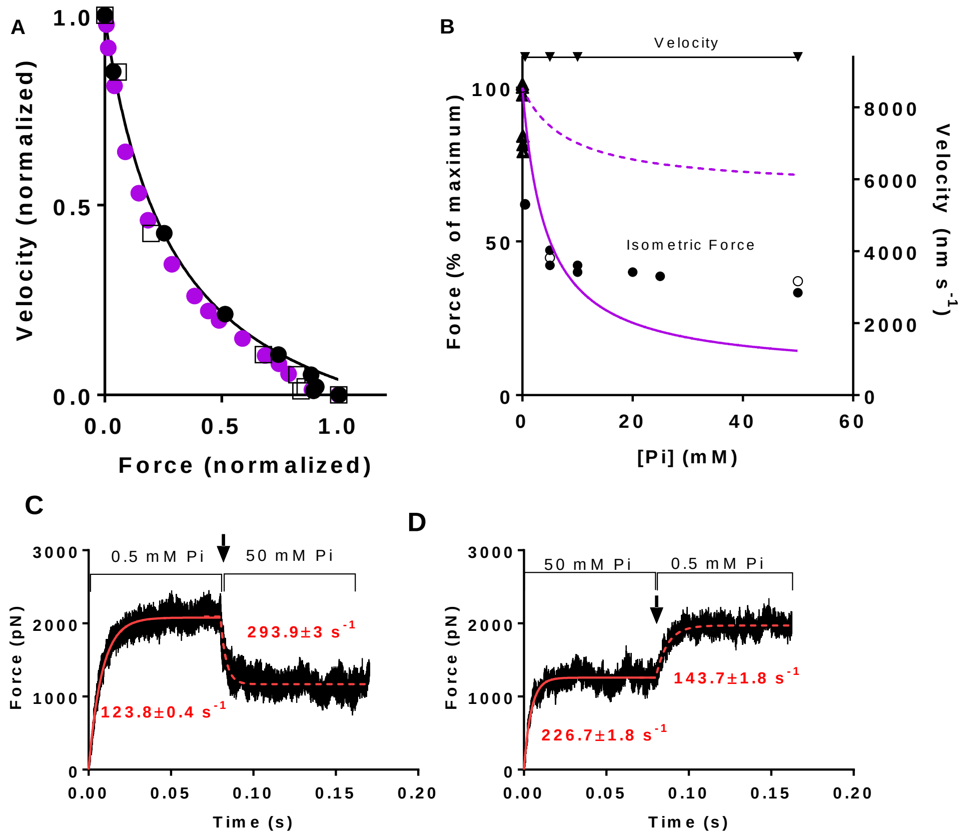

Figure S1. Comparison between model predictions and experimental data (purple symbols) A. Force-velocity data normalized to maximum isometric force and maximum velocity. Absolute velocity values given in B. Experimental data (purple symbols) as in (3) from intact mouse skeletal muscle (49) fitted by the Hill (50) hyperbolic equation (line). Model predictions: black symbols (filled symbols: Monte-Carlo simulations; open symbols: obtained by solving differential equations). Note, both simulated data and experimental data deviate from the hyperbolic relation at high force (c.f. (3, 4, 51)). **B.** Model simulations of the relationship between isometric force and velocity (triangles) on the one hand and concentration of inorganic phosphate on the other, compared to experimental force data (purple) from rabbit psoas myofibril (full line) at 15°C (52) and muscle fiber (dashed line) at 30 ^{o}C (53). The model predictis negligible changes in sliding velocity with altered [Pi] in accordance with experiments (14, 48, 54). Filled symbols: Monte-Carlo simulations; open symbols: obtained by solving differential equations. **C.** Transient tension changes in response to an instantaneous change in [Pi] from 0.5 mM to 50 mM after 0.08 s during an isometric contraction initiated at 0 s. Monte-Carlo simulations assuming a 30 µm long filament and at myosin head density on the surface of 5000 µm^{2}. Rate constants (± 95 % CIs) given in red text in the figures refer to single exponential fits to data by non-linear regression. **D.** Transient tension changes in response to an instantaneous change in [Pi] from 50 mM to 0.5 mM after 0.08 s during an isometric contraction initiated at 0 s. Other conditions similar to those in C. Note similar rates of Pi-transient and initial rate of rise of tension at a given Pi-concenctration. These rates are somewhat higher than the V_{max} of the actomyosin ATPase (83 s^{-1}) in solution because the latter is also influenced by the rate of the ATP hydrolysis/recovery stroke on the myosin active site.

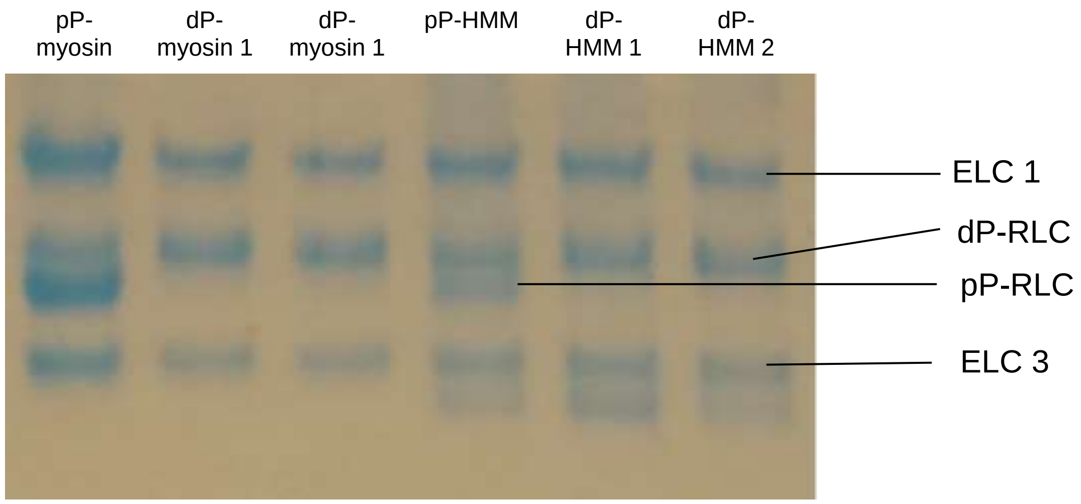

Figure S2. Polyacrylamide gel (12 %) electrophoresis of myosin and HMM in 8M urea with partly phosphorylated (pP-myosin and pP-HMM) and fully dephosphorylated (dP-myosin and dP-HMM) regulatory light chains. ELC 1 – long isoform of myosin essential light chain, dP-RLC – dephosphorylated regulatory light chain, pP-RLC – phosphorylated regulatory chain, ELC 3 – short isoform of myosin essential light chain. Band below ELC 3 with HMM due to 17 kDa fragment of RLC (55) that is co-migrating with the RLC in SDS PAGE. The myosin and HMM preparations were those (labelled 1 and 2, respectively) used for the present experiments, including all data in main Fig. 1 and Fig. S3.

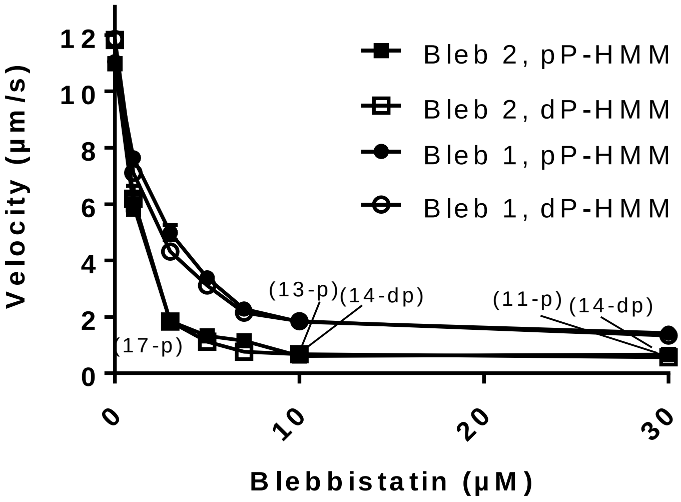

Figure S3. Concentration-response curves for effects of blebbistatin on sliding velocities in the in vitro motility assay. Data obtained using HMM with partially phosphorylated (filled symbols) or fully dephosphorylated (open symbols) regulatory light chains with two batches of blebbistatin (Bleb1 and Bleb 2). Data,given as mean ± 95 % CI, were obtained at130 mM ionic strength (1 mM MgATP). Results from > 20 filaments if not stated otherwise in parentheses (with pP or dP to indicate pP-HMM and dP-HMM, respectively) The data for bleb 2 and dP-HMM are reproduced from Fig. 1 in main paper.

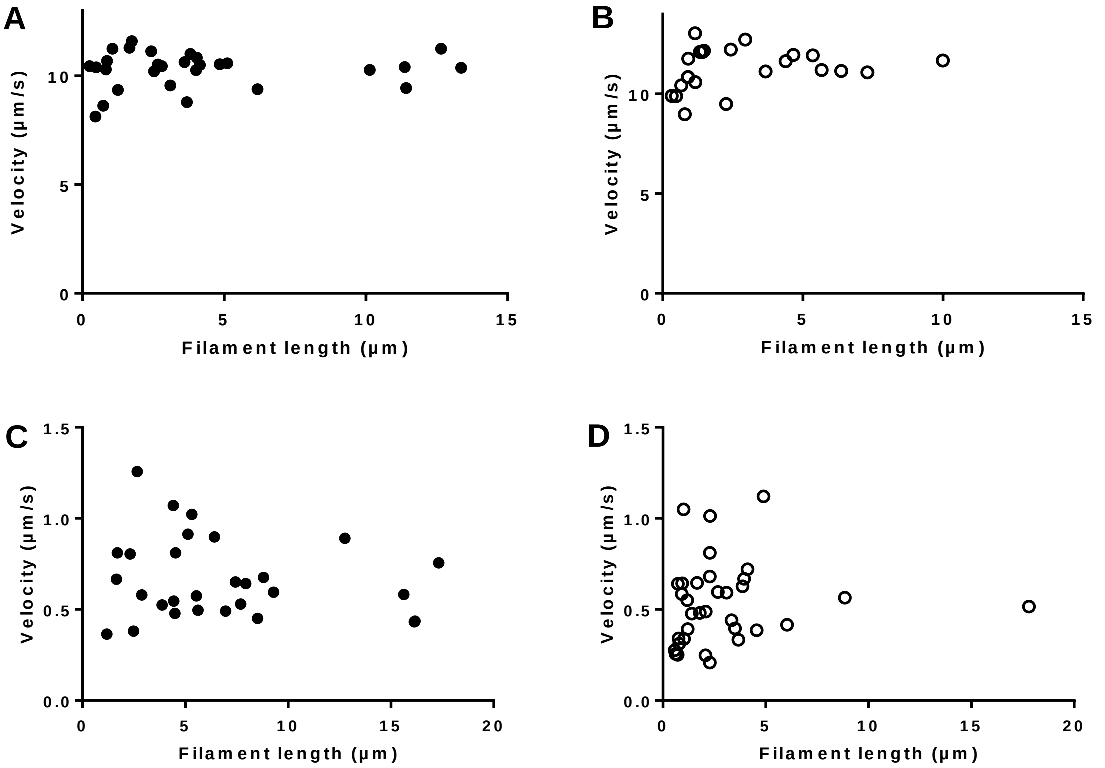

Figure S4. Velocity vs length plots using both partially phosphorylated (pP-HMM (left panels; A, C) and fully dephosphorylated (dP-HMM; right panels; B, D). A and **B,** No blebbistatin. **C** and **D,** 7 µM blebbistatin. An incubation step with blocking actin (non-fluorescent sheared actin filaments; see Methods) was used in the experiments to block dead heads from interfering with the observed fluorescence labelled filaments. The blebbistatin batch with highest activity was used for these experiments.

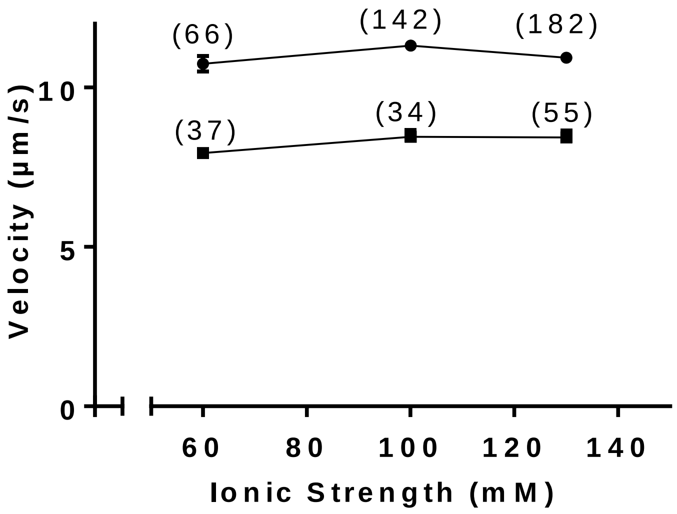

Figure S5. Sliding velocity vs ionic strength of the assay solution using dP-HMM in the absence of blebbistatin. Two different experimental occasions using one HMM preparation with the temperature either 29.8-30.5^{o}C (circles) or 27.2-28.6^{o}C (squares). Data given as mean ± 95 % CI. The numbers in parentheses is the number of filaments studied.

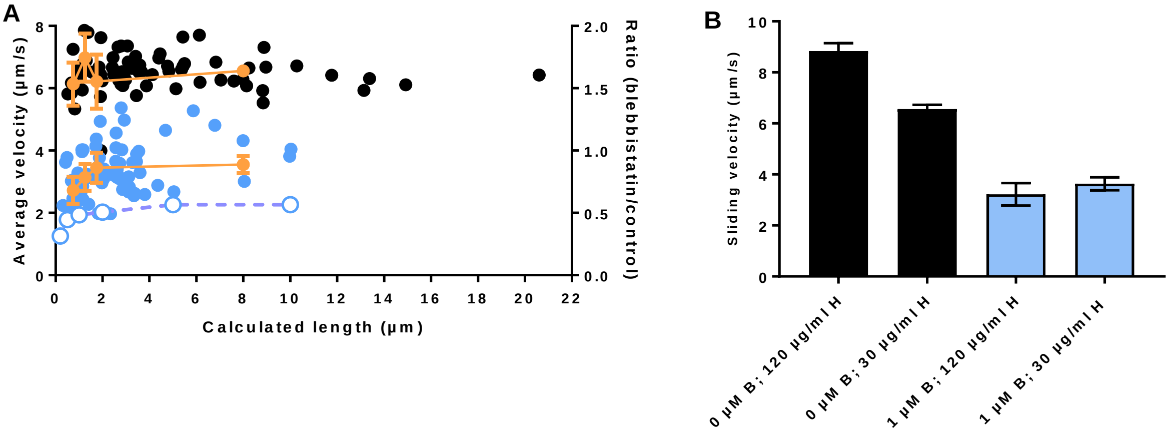

Figure S6. Effect of 1 µM blebbistatin on sliding velocity in the in vitro motility assay – modulation by HMM surface density. A, Velocity vs length plots in an in vitro motility assay after incubation with 30 µg/ml HMM, expected (56) to lower the HMM density on the surface to less than 50 % of that seen after incubation with HMM at 120 µg/ml**.** Data in the absence (black) and presence (blue) of 1 µM S(-) blebbistatin. Orange data points represent mean values ± 95 % CI for ranges of lengths from left to right: 0.5-1.0µm; 1.0-1.5 µm, 1.5-2.0 µm and > 2 µm. Note, lower velocity ratio between blebbistatin and control data for the range 0.5-1 µm compared to lengths > 2 µm. The open blue symbols and dashed line (right axis) gives the ratio between simulated data in the presence and absence of 1 µM blebbistatin (open symbols in main Fig. 2) assuming that the compound lowers k_{P+}'. **B**, Sliding velocity independent of filament length given as mean ± 95 % CI in the absence (black) and presence (blue) of 1 µM S(-) blebbistatin blebbistatin at two different HMM incubation concentration. Note, that reduced HMM incubation concentration lowered sliding velocity in the absence but not in the presence of blebbistatin giving attenuated blebbistatin induced reduction in velocity at the lower HMM incubation concentration. Different myosin preparation than any of those used in main Fig. 1.

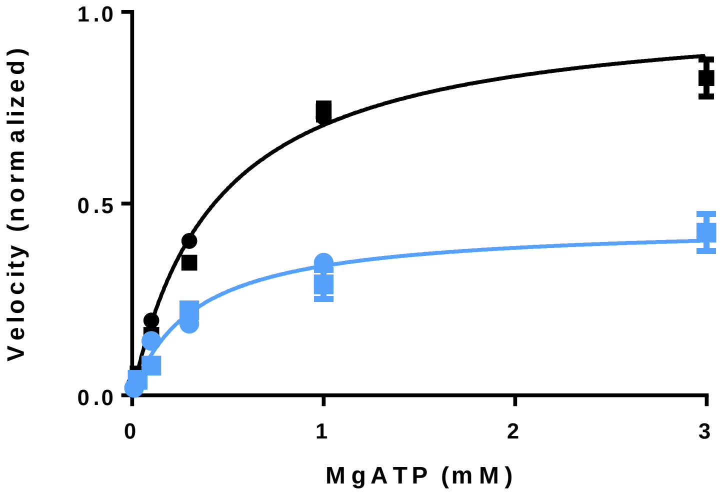

Fig. S7. Actin sliding velocity vs [MgATP] in the presence (blue) and absence (black) of blebbistatin. Data from the same experiments as in main Fig. 2D.Velocity in each experiment was normalized to the velocity at infinite [MgATP] in the absence of blebbistatin as estimated from rectangular hyperbolic fits (Michaelis-Menten type). The absolute value of that velocity value was 12.75 ± 0.35 µm/s (mean ± 95 % CI) in one of the experiments (circles) and 11.66 ± 0.45 µm/s in the other (squares). Curves represent fits of hyperbolic functions to all normalized data with Michaelis-Menten constants V_{Max}= 1.017 ± 0.024 and K_M^v=0.442 ± 0.030 mM under control conditions (black) and V_{Max}= 0.447 ± 0.025 and K_M^v =0.324 ± 0.054 mM in the presence of 1 µM blebbistatin. Temperature: 27.6-30.5 ^{o}C (constant to within 1.0 ^{o}C in a given experiment).

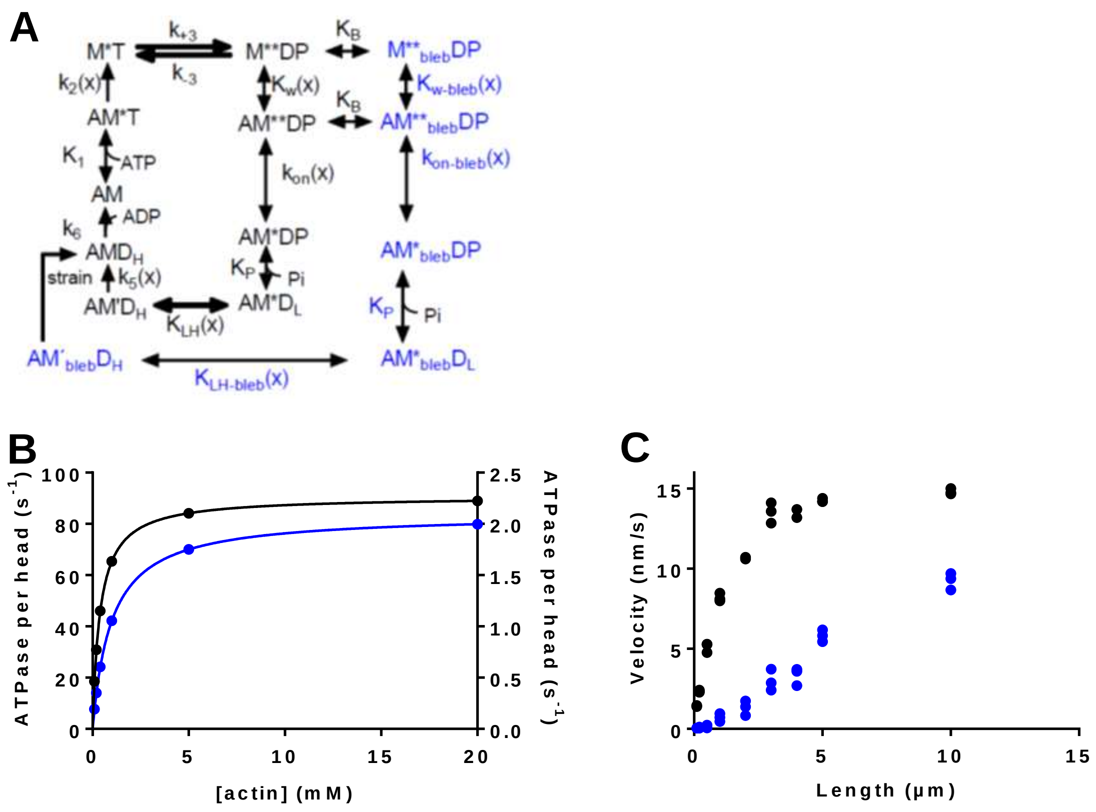

Fig. S8. Simulations using model of Månsson (3). **A**. Kinetic scheme of model adapted with blebbistatin bound states in blue. **B.** Actomyosin ATPase vs actin concentration in solution simulated without blebbistatin (black; left vertical axis) or under the assumption that blebbistatin (saturating concentrations; blue; right axis) reduces the kon(x) rate function 100-fold (to kon-bleb(x)) and the equilibrium constant for the force-generating transition (K_{LH}(x)) more than 5 orders of magnitude. Curves represent fits of hyperbolic functions to the data with Michaelis-Menten constants V_{max}= 90.69 ± 0.01 s^{-1} (mean ± 95 % CI) and K_{ATPase}=0.388 ± 0.0002 mM under control conditions (black; left axis) and V_{max}= 2.096 ± 0.005 s^{-1} and K_{ATPase} =0.987 ± 0.007 mM (blue; right axis) for saturating blebbistatin conditions. **C.** Velocity vs filament length plots simulated without blebbistatin (black) or under the assumption that blebbistatin (blue) reduces both kon(x) and (K_{LH}(x)) as in B. Note appreciable length dependence of the simulated blebbistatin effects on velocity. Blebbistatin concentration assumed to be 2 µM and blebbistatin affinity 5 μM^{-1}. The latter value is five-fold higher than assumed in main Fig. 4 using the expanded version of the model

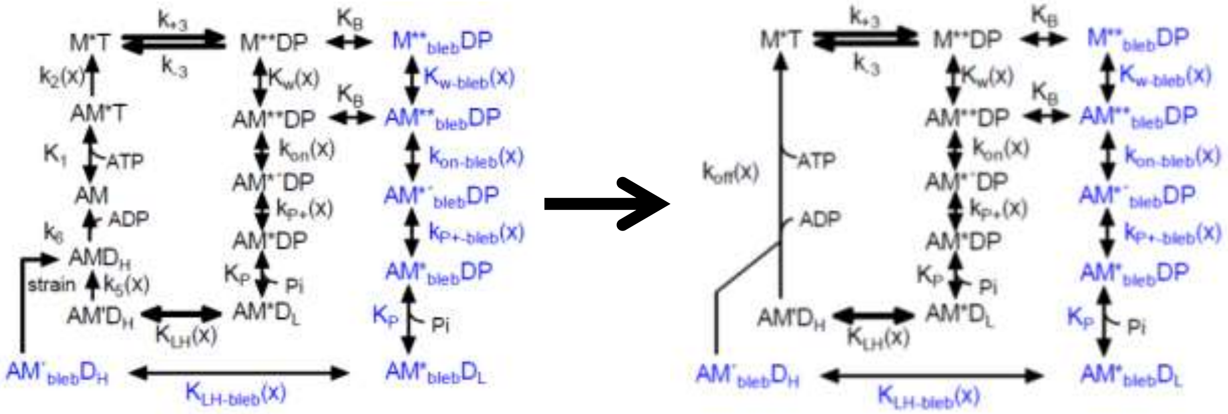

Fig. S9. Reduction of final model (model in Fig. S7 + ideas in (1)) by lumping all rate functions between the AM´D_H and the M*T states into one detachment rate function ($k_{off}(x)$). The reduction is possible because biochemical evidence(41) suggests negligible effect of blebbistatin on that part of the cycle. See further main Fig. 4 for analyses using this model.

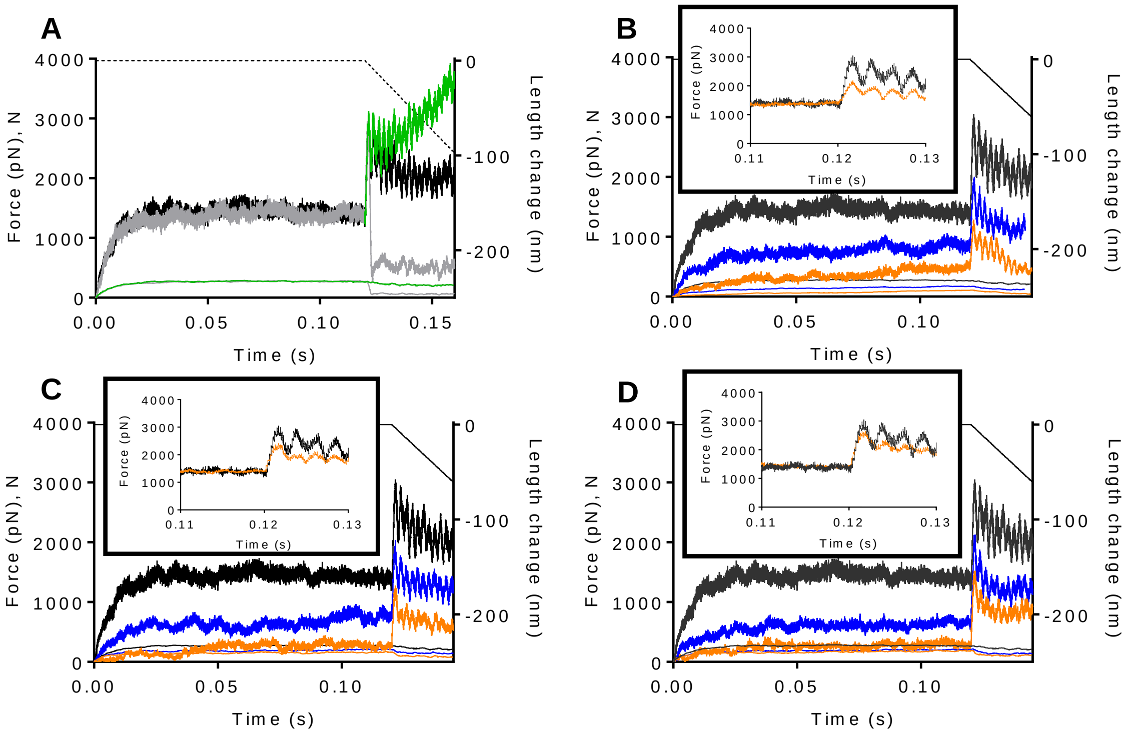

Figure S10. Development of isometric force, number (N) of attached myosin heads and tension responses to stretch during steady-state isometric contraction modeled by Monte-Carlo simulations. Simulated tension responses (thick full lines; left vertical axis) and number of attached cross-bridges (thin full lines; left axis) are shown in response to stretch (dashed black line; right axis). **A.** Control conditions under different assumptions. Grey: no "slippage" between sites (no rapid detachment-reattachment events; Eq. S14). Black: Same parameter values as for grey trace but slippage assumed to occur; Green: Same parameter values as for black trace, but effect of a linear parallel elastic element, simulating titin effects, also included. In the rest of the panels the parallel elastic element is omitted but slippage is included in the model. **B.** Simulated tension responses at 0 (black), 2 µM blebbistatin (blue) and 10 µM blebbistatin (orange) on the assumption that blebbistatin reduces $k_{on+}(x)$ to a degree, sufficient to account for reduced V_{max} of the actomyosin ATPase. **C.** Simulated tension responses at 0 (black), 2 µM blebbistatin (blue) and 10 µM blebbistatin (orange) on the assumption that blebbistatin reduces k_{P+} 650-fold, sufficient to account for reduced V_{max} of the actomyosin ATPase. **D.** Simulated tension responses as in C on the assumption that blebbistatin reduces k_{P+} 650-fold but that it also reduces the difference between the free energy minima of the AM*D_L and the AM´D_H states from 14 to 2.5 k_BT. The insets in B-D show the tension responses to stretch on an expanded time scale for the control condition and in the presence of 10 µM blebbistatin where the latter trace is shifted upwards to make the isometric force level coincide with that under control conditions. The Monte-Carlo simulations were implemented for the model in main Fig. 3A assuming that a 20 µm long actin filament interacts with 5000 myosin heads μm^{-2} on an in vitro motility assay surface. The filament is first assumed to be held isometrically and then (at time 0.05 s) being stretched at 2500 nm/s. Simulations were performed assuming parameter values as at 25-30 ^oC (Tables S1-S2).

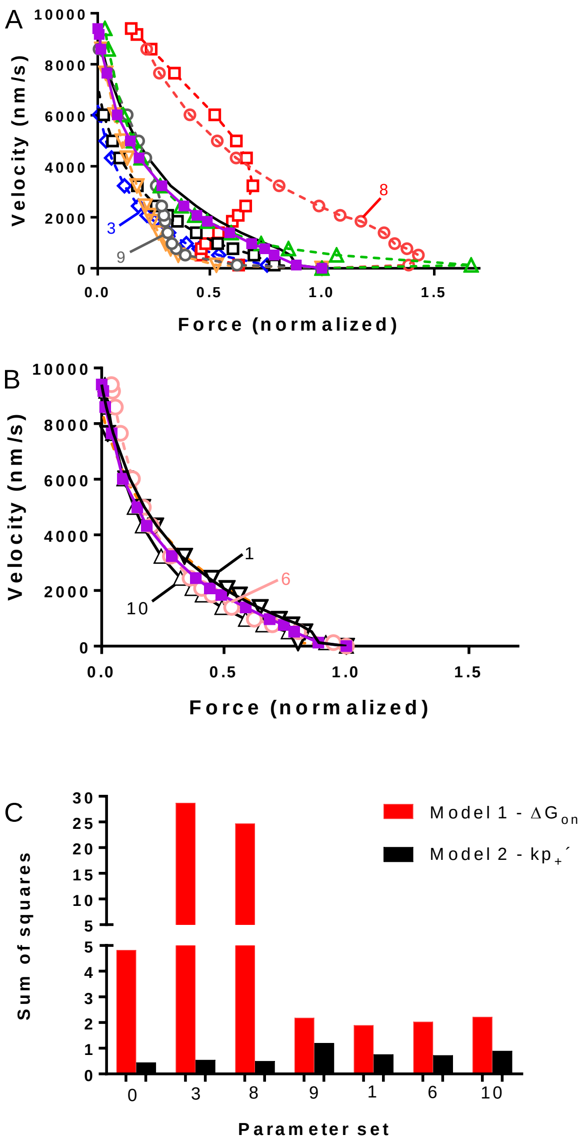

Figure S11. Simulation results obtained by random 25 % increase or 25 % decrease of parameter values (Table S5) compared to parameter values from the literature (Tables S1 and S2). A. Simulated force-velocity data in the absence of blebbistatin using parameter values that give bad fits (open symbols and dashed line) to the experimental data (purple). Full black line without symbols represent simulated results using the parameter values in Tables S1 and S2. The simulations correspond to the parameter values which are given in red font in Table S5. The numbers correspond to parameter set in Table S5. **B.** Simulated force velocity data as in A but using parameter values (black font in Table S5) that give good fits. Corresponding color coding as in A for purple symbols and line and black line without symbols. The numbers correspond to parameter set in C. **C**. Sum of squared deviations between experimental data and simulated data for variables shown in Table S4. The simulations were performed for all sets of parameter values (sets 1,6,10) from Table S5 that give good fits to force-velocity data (Fig. S11A) and for a random selection of three sets of parameter values (sets 3,8,9 in Table S5) giving poor fits to the force velocity data (Fig. S11B). In these sum of squares values contributions for the variable K_M^V was not included. The sum of squares for the standard parameter values are also shown (parameter set 0), including contributions for the variable K_M^V.

Supplementary References

1. Llinas, P., T. Isabet, L. Song, V. Ropars, B. Zong, H. Benisty, S. Sirigu, C. Morris, C. Kikuti, D. Safer, H. L. Sweeney, and A. Houdusse. 2015. How actin initiates the motor activity of Myosin. Dev Cell 33:401-412.
2. Eisenberg, E., T. L. Hill, and Y. Chen. 1980. Cross-bridge model of muscle contraction. Quantitative analysis. Biophys. J. 29:195-227.
3. Mansson, A. 2016. Actomyosin based contraction: one mechanokinetic model from single molecules to muscle? J. Muscle Res. Cell Motil. 37:181-194.
4. Mansson, A. 2010. Actomyosin-ADP states, inter-head cooperativity and the force-velocity relation of skeletal muscle. Biophys. J. 98:1237-1246.
5. Nyitrai, M., and M. A. Geeves. 2004. Adenosine diphosphate and strain sensitivity in myosin motors. Philos. Trans. R. Soc. Lond. B. Biol. Sci. 359:1867-1877.
6. Capitanio, M., M. Canepari, P. Cacciafesta, V. Lombardi, R. Cicchi, M. Maffei, F. S. Pavone, and R. Bottinelli. 2006. Two independent mechanical events in the interaction cycle of skeletal muscle myosin with actin. Proc. Natl. Acad. Sci. U. S. A. 103:87-92.
7. Albet-Torres, N., M. J. Bloemink, T. Barman, R. Candau, K. Frölander, M. A. Geeves, K. Golker, C. Herrmann, C. Lionne, C. Piperio, S. Schmitz, C. Veigel, and A. Månsson. 2009. Drug effect unveils inter-head cooperativity and strain-dependent ADP release in fast skeletal actomyosin. J. Biol. Chem. 284:22926–22937.
8. Persson, M., E. Bengtsson, L. ten Siethoff, and A. Mansson. 2013. Nonlinear cross-bridge elasticity and post-power-stroke events in fast skeletal muscle actomyosin. Biophys. J. 105:1871-1881.
9. Bell, G. I. 1978. Models for the specific adhesion of cells to cells. Science 200:618-627.
10. Curtin, N. A., and R. E. Davies. 1975. Very high tension with very little ATP breakdown by active skeletal muscle. J. Mechanochem. Cell Motil. 3:147-154.
11. Lombardi, V., and G. Piazzesi. 1990. The contractile response during steady lengthening of stimulated frog muscle fibres. J. Physiol. (Lond). 431:141-171.
12. Mansson, A. 1994. The tension response to stretch of intact skeletal muscle fibres of the frog at varied tonicity of the extracellular medium. J. Muscle Res. Cell Motil. 15:145-157.
13. Brunello, E., M. Reconditi, R. Elangovan, M. Linari, Y. B. Sun, T. Narayanan, P. Panine, G. Piazzesi, M. Irving, and V. Lombardi. 2007. Skeletal muscle resists stretch by rapid binding of the second motor domain of myosin to actin. Proc. Natl. Acad. Sci. U. S. A. 104:20114-20119.
14. Caremani, M., L. Melli, M. Dolfi, V. Lombardi, and M. Linari. 2013. The working stroke of the myosin II motor in muscle is not tightly coupled to release of orthophosphate from its active site. J Physiol 591:5187-5205.
15. Joumaa, V., and W. Herzog. 2014. Calcium sensitivity of residual force enhancement in rabbit skinned fibers. Am. J. Physiol. Cell Physiol. 307:C395-401.
16. Rassier, D. E., and I. Pavlov. 2012. Force produced by isolated sarcomeres and half-sarcomeres after an imposed stretch. Am. J. Physiol. Cell Physiol. 302:C240-248.
17. Edman, K. A. 2012. Residual force enhancement after stretch in striated muscle. A consequence of increased myofilament overlap? J Physiol 590:1339-1345.
18. Campbell, S. G., P. C. Hatfield, and K. S. Campbell. 2011. A mathematical model of muscle containing heterogeneous half-sarcomeres exhibits residual force enhancement. PLoS Comp Biol. 7:e1002156.
19. Duke, T. A. 1999. Molecular model of muscle contraction. Proc. Natl. Acad. Sci. U. S. A. 96:2770-2775.

20. Nishizaka, T., R. Seo, H. Tadakuma, K. Kinosita, Jr., and S. Ishiwata. 2000. Characterization of single actomyosin rigor bonds: load dependence of lifetime and mechanical properties. Biophys. J. 79:962-974.
21. Capitanio, M., M. Canepari, M. Maffei, D. Beneventi, C. Monico, F. Vanzi, R. Bottinelli, and F. S. Pavone. 2012. Ultrafast force-clamp spectroscopy of single molecules reveals load dependence of myosin working stroke. Nature methods 9:1013-1019.
22. Brenner, B., and E. Eisenberg. 1986. Rate of force generation in muscle: correlation with actomyosin ATPase activity in solution. Proc. Natl. Acad. Sci. U. S. A. 83:3542-3546.
23. Woledge, R. C., N. A. Curtin, and E. Homsher. 1985. Energetic aspects of muscle contraction. Academic Press, London.
24. Offer, G., and K. W. Ranatunga. 2015. The endothermic ATP hydrolysis and crossbridge attachment steps drive the increase of force with temperature in isometric and shortening muscle. J Physiol 593:1997-2016.
25. Sleep, J., M. Irving, and K. Burton. 2005. The ATP hydrolysis and phosphate release steps control the time course of force development in rabbit skeletal muscle. J Physiol 563:671-687.
26. Ranatunga, K. W. 2010. Force and power generating mechanism(s) in active muscle as revealed from temperature perturbation studies. J Physiol 588:3657-3670.
27. Zhao, Y., and M. Kawai. 1994. Kinetic and thermodynamic studies of the cross-bridge cycle in rabbit psoas muscle fibers. Biophys. J. 67:1655-1668.
28. Linari, M., M. Caremani, C. Piperio, P. Brandt, and V. Lombardi. 2007. Stiffness and fraction of Myosin motors responsible for active force in permeabilized muscle fibers from rabbit psoas. Biophys. J. 92:2476-2490.
29. Nyitrai, M., R. Rossi, N. Adamek, M. A. Pellegrino, R. Bottinelli, and M. A. Geeves. 2006. What limits the velocity of fast-skeletal muscle contraction in mammals? J. Mol. Biol. 355:432-442.
30. Roots, H., G. J. Pinniger, G. W. Offer, and K. W. Ranatunga. 2012. Mechanism of force enhancement during and after lengthening of active muscle: a temperature dependence study. J. Muscle Res. Cell Motil. 33:313-325.
31. Ranatunga, K. W., and M. E. Coupland. 2010. Crossbridge mechanism(s) examined by temperature perturbation studies on muscle. Adv. Exp. Med. Biol. 682:247-266.
32. Hook, P., and L. Larsson. 2000. Actomyosin interactions in a novel single muscle fiber in vitro motility assay. J. Muscle Res. Cell Motil. 21:357-365.
33. Pardee, J. D., and J. A. Spudich. 1982. Purification of muscle actin. Methods Cell Biol. 24:271-289.
34. Kron, S. J., Y. Y. Toyoshima, T. Q. Uyeda, and J. A. Spudich. 1991. Assays for actin sliding movement over myosin-coated surfaces. Methods Enzymol. 196:399-416.
35. Perrie, W. T., and S. V. Perry. 1970. An electrophoretic study of the low-molecular-weight components of myosin. Biochem. J. 119:31-38.
36. Wilson, C., N. Naber, E. Pate, and R. Cooke. 2014. The myosin inhibitor blebbistatin stabilizes the super-relaxed state in skeletal muscle. Biophys. J. 107:1637-1646.
37. Minozzo, F. C., and D. E. Rassier. 2010. Effects of blebbistatin and Ca2+ concentration on force produced during stretch of skeletal muscle fibers. Am. J. Physiol. Cell Physiol. 299:C1127-1135.
38. Sakamoto, T., J. Limouze, C. A. Combs, A. F. Straight, and J. R. Sellers. 2005. Blebbistatin, a myosin II inhibitor, is photoinactivated by blue light. Biochemistry. 44:584-588.

39. Getz, E. B., R. Cooke, and S. L. Lehman. 1998. Phase transition in force during ramp stretches of skeletal muscle. Biophys.J. 75:2971-2983.
40. Lard, M., L. ten Siethoff, J. Generosi, M. Persson, H. Linke, and A. Mansson. 2015. Nanowire-imposed geometrical control in studies of actomyosin motor function. IEEE trans. Nanobiosci. 14:289-297.
41. Kovacs, M., J. Toth, C. Hetenyi, A. Malnasi-Csizmadia, and J. R. Sellers. 2004. Mechanism of blebbistatin inhibition of myosin II. J. Biol. Chem. 279:35557-35563.
42. Gillespie, D. T. 1976. A general method for numerically simulating the stochastic time evolution of coupled chemical reactions. J. Comp. Phys. 22:403-434.
43. Kaya, M., and H. Higuchi. 2010. Nonlinear elasticity and an 8-nm working stroke of single myosin molecules in myofilaments. Science 329:686-689.
44. Brenner, B., L. C. Yu, L. E. Greene, E. Eisenberg, and M. Schoenberg. 1986. Ca2+-sensitive cross-bridge dissociation in the presence of magnesium pyrophosphate in skinned rabbit psoas fibers. Biophys. J. 50:1101-1108.
45. Dantzig, J. A., Y. E. Goldman, N. C. Millar, J. Lacktis, and E. Homsher. 1992. Reversal of the cross-bridge force-generating transition by photogeneration of phosphate in rabbit psoas muscle fibres. J Physiol 451:247-278.
46. Karatzaferi, C., M. K. Chinn, and R. Cooke. 2004. The force exerted by a muscle cross-bridge depends directly on the strength of the actomyosin bond. Biophys. J. 87:2532-2544.
47. Pate, E., and R. Cooke. 1989. A model of crossbridge action: the effects of ATP, ADP and Pi. J. Muscle Res. Cell Motil. 10:181-196.
48. Debold, E. P., M. A. Turner, J. C. Stout, and S. Walcott. 2011. Phosphate enhances myosin-powered actin filament velocity under acidic conditions in a motility assay. Am J Physiol Regul Integr Comp Physiol 300:R1401-1408.
49. Mansson, A., J. Morner, and K. A. Edman. 1989. Effects of amrinone on twitch, tetanus and shortening kinetics in mammalian skeletal muscle. Acta Physiol. Scand. 136:37-45.
50. Hill, A. V. 1938. The heat of shortening and the dynamic constants of muscle. Proc. Royal Soc. B 136–195 126:136-195.
51. Edman, K. A., A. Mansson, and C. Caputo. 1997. The biphasic force-velocity relationship in frog muscle fibres and its evaluation in terms of cross-bridge function J. Physiol. (Lond). 503:141-156.
52. Tesi, C., F. Colomo, N. Piroddi, and C. Poggesi. 2002. Characterization of the cross-bridge force-generating step using inorganic phosphate and BDM in myofibrils from rabbit skeletal muscles. J Physiol 541:187-199.
53. Coupland, M. E., E. Puchert, and K. W. Ranatunga. 2001. Temperature dependence of active tension in mammalian (rabbit psoas) muscle fibres: effect of inorganic phosphate. J Physiol 536:879-891.
54. Cooke, R., and E. Pate. 1985. The effects of ADP and phosphate on the contraction of muscle fibers. Biophys. J. 48:789-798.
55. Wagner, P. D. 1984. Effect of skeletal muscle myosin light chain 2 on the Ca2+-sensitive interaction of myosin and heavy meromyosin with regulated actin. Biochemistry. 23:5950-5956.
56. Bengtsson, E., M. Persson, M. A. Rahman, S. Kumar, H. Takatsuki, and A. Mansson. 2016. Myosin-Induced Gliding Patterns at Varied [MgATP] Unveil a Dynamic Actin Filament. Biophys. J. 111:1465-1477.
